# Towards a sustainable high-quality materials data ecosystem: frameworks and strategies

**DOI:** 10.1093/nsr/nwag108

**Published:** 2026-02-14

**Authors:** Bing He, Kaixuan Wang, Zhou Jiang, Yueyu Zhang, Maxim Avdeev, Siqi Shi

**Affiliations:** State Key Laboratory of Materials for Advanced Nuclear Energy & School of Computer Engineering and Science, Shanghai University, Shanghai 200444, China; State Key Laboratory of Materials for Advanced Nuclear Energy & School of Computer Engineering and Science, Shanghai University, Shanghai 200444, China; State Key Laboratory of Materials for Advanced Nuclear Energy & School of Computer Engineering and Science, Shanghai University, Shanghai 200444, China; State Key Laboratory of Materials for Advanced Nuclear Energy & School of Materials Science and Engineering, Shanghai University, Shanghai 200444, China; School of Chemistry, University of Sydney, Sydney, NSW 2006, Australia; State Key Laboratory of Materials for Advanced Nuclear Energy & School of Materials Science and Engineering, Shanghai University, Shanghai 200444, China; Materials Genome Institute, Shanghai University, Shanghai 200444, China

**Keywords:** high-quality materials data ecosystem, materials science, synchronous data quality optimization

## Abstract

High-quality data have increasingly been recognized as a critical catalyst in advancing artificial intelligence-driven materials discovery and design. However, the current data generation and management systems in materials science are often deficient, resulting in an abundance of low-quality data characterized by heterogeneous formats, incomplete entries and semantic inconsistencies. Such a fragmented data landscape limits direct access to reliable datasets, making it inevitable to allocate substantial resources toward data consolidation and governing existing data. Here, we propose a comprehensive quality-controlled framework encompassing all three principal stages of the research lifecycle—acquisition, management and utilization. By establishing stage-specific requirements, materials data quality can be concurrently optimized and enhanced throughout the materials research and development process. Furthermore, we propose five strategic initiatives to operationalize this framework, including an integrated computational/experimental ‘data factory’ for data acquisition and an automated toolkit for data quality assessment. This work aims to embed quality-governed production directly into scientific inquiry, fostering a sustainable and robust materials data ecosystem.

## Teaser

A full-process framework covering all research data stages for quality control and improvement is introduced, with community implementation measures to integrate data quality management into routine research, build a high-quality materials data ecosystem and enable data-to-knowledge conversion.

## INTRODUCTION

Artificial intelligence (AI) is disrupting materials science by autonomously discovering high-dimensional, theory-transcendent patterns and enabling probabilistic extrapolation beyond trial-and-error approaches, catalyzing the emerging interdisciplinary domain known as ‘AI for Materials’ [1–5]. However, the extraordinary capabilities exhibited by AI are fundamentally dependent on the quality of underlying data. Data, particularly high-quality data, not only furnish an efficient and robust substrate for model training and tuning, but also constitute the foundational guarantee of predictive accuracy and generalizability [6,7].

The investigation of data quality has evolved alongside information systems since the late 20th century [8,9]. Initially, data quality was commonly equated with information quality, defined as the degree to which data collectively meet the requirements of potential application contexts [10]. Given its heterogeneous and multifaceted nature, data quality is frequently conceptualized within a multidimensional framework that captures both contextual relevance and utility [11]. In the 21st century, this focus has permeated diverse disciplines, including biomedicine [12,13], geospatial science [14] and electronic device engineering [15]. More recently, with the ascendance of AI-related intelligent technologies, the scientific community has articulated more rigorous quality standards for exploitable datasets. For instance, our previous work established that data quality governance should prioritize the quantity of samples, the diversity of features and the critical balance between them [16]. Subsequently, to address the issue from the perspective of training reliable machine learning (ML) models, we categorized data quality into three principal dimensions: inherent, contextual and quantitativeness, encompassing a total of 12 quality indicators [17]. Grounded in an ‘AI-ready data’ viewpoint, Lu *et al*. [18] summarized the attributes that confer readiness for AI applications, from both individual sample data and entire dataset perspectives.

While these contributions elucidate the concept of high-quality data for researchers at the forefront of scientific inquiry, and offer guidance for auditing and refining existing data quality, concrete directives for the generation and identification of high-quality data remain undeveloped. Due to the absence of widely accepted data standards [19,20] and robust data quality management frameworks, the materials science community has long contended with a fragmented environment characterized by isolated data silos and heterogeneous data formats. Rapid access to high-quality materials data remains challenging.

To navigate this complexity, researchers must expend considerable effort employing disparate cleaning techniques—ranging from statistical analysis and visualization [21] to specialized ML algorithms [22]—to rectify fundamental quality issues such as outliers, formatting inconsistencies and redundancies. In an effort to standardize these practices across the ML pipeline, Liu *et al*. [23] proposed a domain-knowledge-embedded data quality governance framework—generalizable yet tailored to the full lifecycle of ML workflows in materials science. This framework defines *what* to evaluate through nine quality dimensions, guides *when* to conduct governance using lifecycle models, and specifies *how* to detect and resolve quality issues through processing models. Extending this, the authors narrow their lens to data accuracy, embedding materials heuristics into anomaly-detection workflow that accelerates ML-driven design and discovery [24]. Nevertheless, significant structural deficiencies remain in current materials data quality governance practices: (i) the *absence of upstream governance and escalated downstream burdens*: ineffective upstream quality management mechanism shifts data governance burdens to end-users, who, lacking insight into data provenance, incur technical debt through costly governance and forensic validation efforts; (ii) *questionable governance results*: end-users cannot effectively discern the causes of discrepancies in multisource data. Excessive data cleaning risks erasing critical physical correlation features or typical data points within datasets. Domain knowledge helps alleviate this issue, but the effectiveness remains uncertain because of the insufficient data-related descriptive information; and (iii) *project-bound governance and the reusability trap*: project-bound data-quality governance create non-transferable artifacts, locking governance endeavors into endless rework.

Synthesizing current perspectives on data quality, researchers share a singular vision: the creation of standardized datasets that, through rigorous curation and verification, exhibit structural consistency, high reliability, interpretability and seamless interoperability. Achieving this ideal is not a discrete task but a continuous requirement. Data quality issues can manifest at any stage of the research lifecycle, from generation and storage to management and application. Consequently, ensuring data integrity is a systematic engineering endeavor that necessitates synergistic collaboration among stakeholders and iterative refinement across all stages. Currently, the materials science community lacks such a holistic approach.

To bridge this gap, we propose a comprehensive, quality-controlled framework spanning the three core pillars of the materials research and development lifecycle: acquisition, management and utilization. By defining stage-specific quality requirements, this framework enables governance activities to be embedded into daily research routines rather than being deferred as an afterthought. This proactive approach prevents the accumulation of technical debt and quality degradation. Furthermore, to operationalize this framework, we introduce five actionable strategic initiatives tailored to each stage’s unique data activities. Our goal is to shift the paradigm of materials research: transforming high-quality data from an arduous, intentional deliverable into an inherent output of scientific inquiry. This transition will foster a robust materials data ecosystem, ultimately accelerating the conversion of raw data into actionable scientific knowledge.

## DATA QUALITY CONTROL REQUIREMENTS ACROSS DIFFERENT MATERIALS RESEARCH AND DEVELOPMENT STAGES

**Figure 1.**
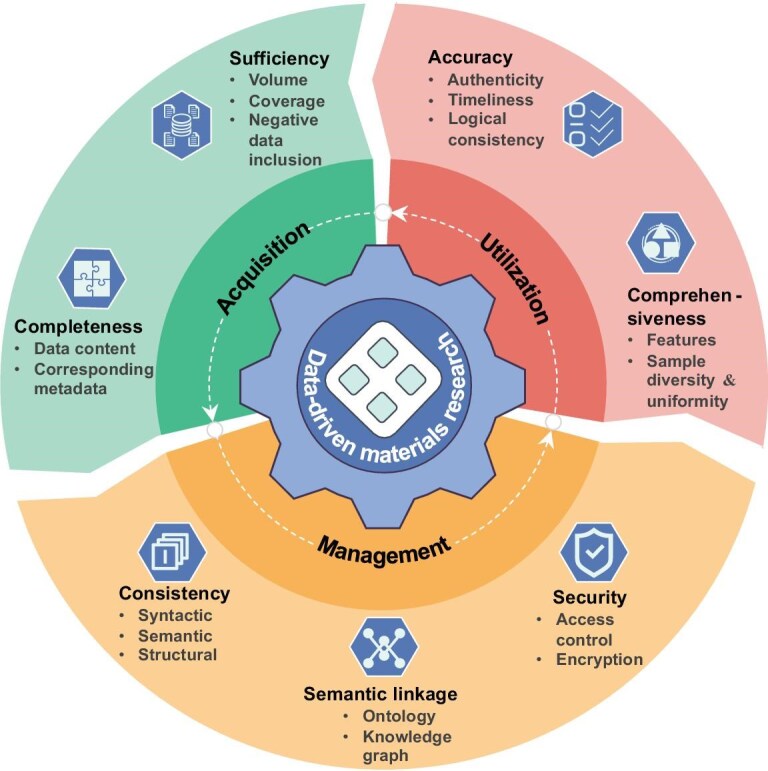
Data quality control requirements across different data lifecycle stages.

The overall reliability of industrial artifacts is fundamentally contingent upon the rigorous quality control across the entire production chain of their constituent components. Similarly, the continuous generation, supply and delivery of high-quality materials data are inextricably predicated upon the meticulous quality governance exercised at every data-handling stage of the research workflow. In this section, in accordance with the inherent progressive refinement of scientific value throughout the research journey, we divide materials data lifecycle into three major stages: acquisition, management and utilization. Stage-specific data quality control requirements and their necessities are then defined and detailed. A stepwise data quality control framework with ‘front-loaded quality checkpoint’ is thus constructed (Fig. 1). By specifying data-quality targets for each stage, researchers are facilitated to proactively inspect and immediately correct low-quality data issues, mitigating the progressive accumulation and downstream propagation of data quality-related technical debts.

### Data acquisition stage

Data acquisition is the starting point not only for data-driven scientific research, but also for data quality governance. In the materials domain, there are mainly two ways for acquiring data: (i) generating massive reliable data through high-throughput (HT) experiments and/or computations; and (ii) extracting valuable information from existing scholar documentation, including peer-viewed literatures, patents and reports. In both cases, two fundamental requirements should be satisfied: sufficient data and complete data.

#### Sufficient data

In an idealized ‘data-rich’ world, complex materials systems can be directly analyzed via data-driven algorithms and ML models, eliminating the necessity for system decomposition, a *priori* simplification, or detailed mechanistic insights into how each constituent subcomponent works [25]. However, in virtually all cases, the *de facto* situation remains far from ideal. Due to inherently complex generation processes and relatively high economic costs, the materials data landscape has long been characterized by two remarkable features: data scarcity and distribution disparity [26,27].

From a holistic standpoint, the aggregate quantity of recorded materials data is limited to the order of tens of millions, significantly below the theoretical estimates. For quinary-element compounds, the estimated figure exceeds 65 million, escalating to several hundred million when accounting for diverse physicochemical properties and synthesis conditions. Besides this, marked data scale disparities exist in different materials categories. Computational datasets dwarf experimental observations by orders of magnitude (Materials Project [28]: >130 000 inorganic compounds; AFLOW [29,30]: ~3 550 330 compounds versus HTEM [31]: ~80 000 distinct material samples). The disproportionate coverage across material classes is more pronounced. While the inorganic crystal structure database (ICSD) [32,33] has amassed over 300 000 crystal structure data, typical catalyst datasets remain constrained to several thousand entries at maximum [34]. Due to the small available data amount and high-dimensional features space, several problems arise when applying data-driven research techniques, specifically ML models for addressing category-specific tasks [35,36]: (i) difficulty in regulating to minimize overfitting or underfitting risks; (ii) poor predictive capabilities of resulting models; and (iii) potential deviations between prediction results and factual observations. Moreover, the data scarcity complicates domain adaptation for general-purpose large language models (LLMs), exacerbating their susceptibility to catastrophic forgetting [37], and hindering the iterative optimization of specialized ML models.

Beyond merely pursing data volume, sufficiency in materials science fundamentally constitutes an intelligent sampling optimization problem. The scientific goal is to efficiently reduce epistemic uncertainty within high-dimensional feature spaces of materials while minimizing cost. This necessitates a physical-informed, co-designed sampling paradigm. Purely data-driven strategies like active learning iteratively select samples that maximize model improvement, whereas integrating physical priors, such as phase diagram rules or thermodynamic constraints, guides the search away from physically implausible regions. For instance, when exploring novel high-entropy alloys, prescreening compositions using criteria like mixing enthalpy and structural stability can dramatically reduce the space requiring first-principle calculations. Nevertheless, determining the precise data volume required for sufficiency remains challenging, as it depends on multiple factors. A relatively critical role is the scope of the materials research object. For narrowly focused studies (e.g. the tensile strength of titanium alloy Ti-6Al-4V), a few hundred well-controlled experimental records are usually sufficient. But for broadly generalizable models (e.g. for predicting the mechanical properties of all titanium-based alloys), tens to hundreds of thousands of data points are needed to capture full variability and ensure generalizability.

At the same time, keeping neutrality toward all materials data during the data acquisition stage is crucial. Negative or non-positive materials data points, such as failed syntheses, phases with mediocre properties, or experiments yielding no targeted product under rigorously controlled conditions, are frequently discarded due to perceived lack of immediate value [38]. But they may occupy underrepresented regions of the compositional and processing parameter space. Operationally, two requisites should be met when dealing with negative data: (i) ensuring full capture of each sample’s contextual generation conditions; and (ii) each data point should be accompanied by explicit flags (e.g. ‘synthesis_failed’, ‘property_below_threshold’) or more universal flags (e.g. success, failure, anomalous) rather than exclusion. Providing specific reasons or codes for classification as non-positive (e.g. no phase formed, property < threshold) is also recommended and recording them together into descriptive, provenance, technical metadata in the metadata filling framework (see Section S1 of the Supplementary data). Such labeled negative samples not only delineate sharp decision boundaries for ML models [39] but also enable counterfactual validation [40,41] and failure-mode analysis in meta-learning frameworks, thereby elevating both generality and credibility of data-driven materials discoveries.

#### Complete data

The imperative for data completeness is inextricably linked to the foundational scientific tenets of reproducibility and verifiability. Such completeness transforms documentation from a procedural task into the indispensable cornerstone of an interoperable and cumulative knowledge infrastructure. It is this foundation that enables robust data integration and transformative knowledge discovery across disparate research teams and temporal scales, while simultaneously underpinning effective data quality governance. Historically, exorbitant storage costs and technical bottlenecks rendered selective archiving economically rational, yet eroded verifiability and reliability of scientific discoveries. Despite remarkable increases in storage density and plummeting cost in the 21st century, complete data documentation has not yet become normative behavior. One critical factor perpetuating such deficient practice is the ambiguous comprehension of completeness principles among data producers, leading to unsystematic planning and compromised integrity in data collection and documentary operations.

In this context, we contend that two fundamental criteria must be fulfilled to meet the completeness requirements: the data content itself and its corresponding metadata. The former emphasizes the exhaustive preservation of all obtainable result information, eliminating missing entries and preventing unnecessary procedural repetitions caused by inadvertent omissions. For instance, it is suggested that the comprehensive and systematic collection of multidimensional materials properties related to crystal structures should be conducted, instead of merely concentrating on measuring typical properties like formation energy, band gap, elastic tensor etc. The latter places greater emphasis on the extensive capturing of data-related ‘environmental information’ and various raw processing files. Metadata is broadly regarded as ‘the data about data’ [42,43]. With structured information of both external attributes and internal features of data objects, it provides a panoramic navigation framework, empowering human scientists and even automated agents to precisely locate, efficiently parse and repurpose the data resources of interest [44]. Additionally, the presence of metadata maximizes the adherence to the findable, accessible, interoperable and reusable (FAIR) scientific data principles [45,46]. Without the contextual explanation provided by metadata, data might only be regarded as discrete character collections gathered for certain purpose, the value of which diminishes due to ineffective information transmission.

During the data acquisition stage, complete metadata documentation is like detailing the ‘instruction manual’ for the data. To foster clear understanding of the complete metadata documentation, we establish a metadata filling framework that encompasses five fundamental metadata types: descriptive, provenance, technical, structural and preservation metadata. Detailed information of these metadata is presented in Section S1.

Given the intrinsic diversity of studied materials, variations in data generation methods and tools, as well as differences in subjective perceptions among researchers, establishing a general data recording model is quite a challenging task. The formulation and effectiveness of domain-specific data documentation protocols require contextual alignment with certain research scenarios. Accordingly, this study focuses merely on identifying the core elements required for complete scientific data documentation. In addition, entries additions, modifications or deletions of data specification will be required to adapt the evolving changes as the continuous emergence of novel tools and application scenarios.

Technically, it is recommended to adopt a full-process metadata collection and registration mechanism driven by automation technology [47]. Through standardized definition of data collection protocols, robust deployment of automated acquisition pipelines, and rigorous application of completeness validation checks, the risk of data omissions resulting from human oversight can be mitigated. For laboratories currently lacking full automation, a lightweight interim practice is suggested: formulate and, in a timely way, update the complete record specification agreed within the group based on specific research scenarios and the metadata filling framework mentioned in Section S1. High-volume raw files (e.g. full trajectories or spectra) should be archived in compressed form and/or offline storage, with only their access paths recorded. This ensures core reproducibility at negligible extra cost until automated systems become available.

### Data management stage

The data management stage serves as the critical bridge between data acquisition and user-friendly utilization. Without immediate intervention, the high-velocity expansion of materials data would overwhelm researchers and entrench the ‘data silo’ effect, impeding cross-system integration and interdisciplinary knowledge transfer. As the pivotal stage for enhancing data quality and ensuring standardization and broad usability, three quality aspects require prioritized consideration: consistent data; semantic linkage; and data security.

#### Consistent data

Data consistency denotes a type of data state, i.e. being logically coherent and free from contradictions among multisource datasets, while without violating completeness constraints, data editing rules and other formalisms. The materials data with a unified format can significantly reduce time expenditure on data preprocessing steps like format conversion, semantic interpretation and alignment, enhancing research efficiency and preventing potential scientific cognitive biases introduced by data heterogeneity.

However, in materials science, the broad spectrum of research subjects, coupled with diverse disciplinary conventions for knowledge representation and unique data cultures, leads to the persistent issue of data inconsistency. Moreover, the strong encouragement for first-time innovation from scientific community contributes to the proliferation of tools for solving diverse tasks. These tools often have their own protocols, requiring input in a rigid, self-defined form and generating output in non-standard, even proprietary forms. The interplay of these factors sustains a detrimental cycle, whereby the challenges associated with integrating data from multiple sources intensify the divergence of research methodologies among various subdisciplines.

Consequently, addressing data inconsistency issues is an urgently imperative, yet tough and potentially time-consuming task. It requires researchers to combine data generation ‘context’ to conduct meticulous semantic interpretation, and consistency checks of each data item. In practice, three principal inconsistency dimensions should be resolved: (i) *Semantic heterogeneity*: this refers to the divergence in interpretation or meaning of data terminological expression, which typically stems from variances in domain-specific knowledge representation schemas, cognitive frameworks or contextual associations. Specifically, semantic heterogeneity can be categorized into two main types: (a) different terms or symbols refer to the same entity (e.g. one certain molecule can be identified in various ways such as IUPAC name [48], conventional name or SMILES [49] notation); and (b) the same data representation acquires different meanings in different contexts (e.g. zinc oxide nanoparticles may be classified as catalyst in catalysis science, yet zinc oxide or nanoparticles alone do not inherently possess catalytic properties). Currently, there exists no natural approach to handle this semantic fuzziness. It may be achieved through multisource data cross-validation and discrepancy resolution with comparative analysis. (ii) *Syntactic heterogeneity*: this denotes syntactic differences in data models, often evident in disparities in word-formation rules, morphological forms and granularity of data representation. For example, the same physical quantity may have multiple numerical representations due to varying measurement systems, as exemplified by density, which could be recorded in kg/m^3^, kg/mm^3^ or g/cm^3^. A pragmatic solution may require the development of dedicated conversion algorithms or standardized processing modules. (iii) *Structural heterogeneity*: this manifests in data storage formats, schema architectures, encoding protocols and structural approaches (structured, semi-structured and unstructured data) for organizing data content. For instance, crystallographic unit cell parameters may be stored as either an integrated entity (e.g. a, b, c, α, β, γ) or discrete components with separate data fields.

Notably, data consistency is not an absolute virtue but context-dependent. It should be tightened or relaxed according to the intended research scenario.

#### Semantic-linked data

Viewed holistically, the macroscopic behavior of materials is a complex function of chemical composition, crystal structure, intrinsic properties, processing parameters and characterization conditions. The non-linear coupling mechanism between these variables dictates the complexity across the entire innovation chain of advanced materials. Establishing the materials data correlation system essentially represents the reconstruction of the ‘genetic expression atlas’ of materials in digital space. This system helps researchers overcome limitations of univariate analysis in traditional materials research, enabling in-depth understanding of multifactor synergistic mechanism. It also functions as a transformative tool for intelligent materials development and precise performance tuning.

Although of paramount importance, the systematic development of data relevance networks remains notably underdeveloped and even often neglected within contemporary materials data management systems. One common method for such networks is to integrate geographically dispersed heterogeneous data into a consolidated platform via multiple extract–transform–load (ETL) pipelines, with data linkages enforced by foreign key constraints [50]. For example, experimental characterization data, e.g. crystal structure parameters from X-ray diffraction (XRD) and computational simulation data (e.g. electronic structure and thermodynamic properties from first-principles calculations) of a material can be integrated under the same material identifier. This approach creates a unified view that links diverse datasets. Nevertheless, these data associations are predominantly implicit connections, lacking explicit semantic representation, which hinders precise interpretation and the dependable propagation of logical relationships across datasets. Furthermore, in complex distributed storage architectures, maintaining referential integrity constraints is a daunting task. Any structural modification may trigger cascading update issues, while system migrations or schema transformation frequently disrupt linkages, resulting in enduring data fragmentation and the formation of isolated data silos.

Semantic web technology offers an alternative and effective strategy for establishing semantically rich associations, especially in scenarios requiring distributed storage, cross-institutional data sharing or large-scale complex semantic correlations [51]. By employing logical-level semantic mappings instead of physical storage-level integrations, it avoids the substantial overhead of physical data consolidation in distributed systems. At the same time, it allows for more flexible characterization of data relationships and explicitly encodes abundant semantic information to support consistent understanding across cross-institutional collaborations. That, in turn, facilitates a more thorough investigation of material mechanisms. It pushes researchers to treat each individual data point as a coordinate in multidimensional phase space, instead of an isolated research output, which is particularly valuable for disentangling large-scale complex semantic correlations. Consequently, researchers’ capability for holistic analysis of material behavior is significantly enhanced [52].

#### Data security

Data featuring complete and detailed contextual descriptions, standardized formats and unambiguous semantic annotations are of paramount significance in accelerating materials discovery and innovation. Accordingly, in a data-driven materials research paradigm, data security transcends a mere technological safeguard. It has evolved into a vital defensive ‘bulwark’ that guarantees data integrity, fidelity and consistency, and secures the trustworthiness of scientific conclusions.

A robust data storage infrastructure, incorporating distributed redundant backup plans, provenance logging and tracking mechanisms, and version control, could prevent devastating high-quality data loss. Furthermore, stringent security measures, such as encrypted archiving, multilayered access control lists and distributed ledger technology, enable scientific databases to effectively counter illegal data operations, including unauthorized access, data tampering and theft. In addition, the deployment of user authentication and data validation mechanisms ensure uploaded data are reliable and authoritative. This prevents the infiltration of unverified or low-credibility data into research ecosystems which may otherwise induce ‘data contamination’ risks.

### Data utilization stage

Throughout the entire scientific journey, data utilization is the core stage for fully unleashing data values. Researchers identify and extract hidden patterns in complex multidimensional datasets and formalize them into verifiable scientific principles and cognitive models for similar task-solving. Through systematic processing at the data management stage, the quality of multisource heterogeneous data achieves remarkable improvements. Nevertheless, they may still harbor potential low-quality data issues, such as completeness loss and questionable accuracy. Rigorous data quality assessment prior to actual data utilization is an essential prerequisite for ensuring the effective realization of data value. Beyond examining the quality characteristics of completeness, formatting consistency and semantic linkage required in previous stages, two additional and equally important dimensions should also be checked: accuracy and comprehensiveness.

#### Accuracy

Data accuracy, the extent to which real-world material properties are faithfully reflected and adhere to domain-specific theoretical rules, constitutes a central pillar in upholding the credibility of scientific discoveries [53]. Highly accurate, reliable data not only sustain persistent academic citation over extended periods, forming the cornerstone of credible research conclusions, but also accrue scientific value over time. In the era of data sharing and interdisciplinary collaboration, they foster effective communication among different teams and organizations, minimizing misinterpretations and redundant efforts stemming from data issues, and thereby enhancing mutual trust.

However, erroneous data records are ubiquitous. Even carefully compiled data may still contain hidden inaccuracies and outliers [54]. Incorrect data may pose risks of destabilizing the theoretical framework of any discipline fundamentally predicated upon such data. Moreover, data inaccuracy potentially affects functional performance and safety of the final material products. Erroneous materials data carry a unique cascading amplification effect. Initial measurement errors at the microscopic scale can be amplified by non-linear mechanisms in cross-scale modeling, ultimately leading to systematic errors in macroscopic property predictions of material components or products. Therefore, evaluating materials data accuracy before its actual usage is necessary, which guarantees the data are free from noise and can serve as reliable signposts of scientific discovery.

Specifically, three dimensions need to be carefully checked: authenticity, timeliness and logical consistency. The verification of data authenticity relies on the provenance information recorded in the associated metadata. This provenance indicates whether the data come from reliable sources and reflect objective physical/chemical facts in a truthful and accurate manner, rather than being artificially crafted. As a proxy for assessing reliability and accuracy, timeliness, synonymous with data freshness, quantifies the temporal validity of data entities through currency and update frequency metrics. Rigorous monitoring of timeliness is critical for ensuring synchronization with the latest scientific understanding. Outdated data may induce systematic bias in scientific inferences, attributable to insufficient measurement precision. Lastly, the evaluation of logical consistency assesses whether data conform to the constraints imposed by theoretical principles. For instance, recorded temperature measurements should not be lower than absolute zero, which is the theoretical minimum temperature in physics. In practical applications, however, this requirement may conflict with the data authenticity. Therefore, it is advisable to conduct a simultaneous evaluation of both aspects during the validation process.

#### Comprehensiveness

This checks whether the dataset covers all anticipated sample categories and the integrity of feature fields. A dataset deficient in comprehensiveness is analogous to an incomplete puzzle; even if each piece is highly precise, it cannot reconstruct the complete cognitive framework in materials science. Generally, the systematic examination of data comprehensiveness should be conducted through dual-dimensional assessment, encompassing feature integrity (horizontally) and sample diversity and uniform distribution (longitudinally), which ensure scientific discoveries are both mechanistically explanatory and broadly applicable.

*The comprehensiveness of data features*. Data features, also called fingerprints or descriptors when constructing ML models, commonly refer to the description of intrinsic characteristics or quantifiable attributes of the observed object [5]. Each feature embodies a specific physical/chemical property that, to varying degrees, dictates underlying functional behavior of materials. Ideally, comprehensive materials data features should encompass all aspects of the materials tetrahedron framework, i.e. composition–structure–property–processing. However, data focusing on one or two facets can still be deemed relatively comprehensive if they meet the following conditions: (i) they fully cover all key influencing factors and variables of the target facet that are closely related to the research objective; (ii) they capture the quantitative dependence relationships between these variables and the material functions/mechanisms concerned; and (iii) they achieve sufficient data precision, consistency and reproducibility to support reliable mechanistic analysis or model construction. Crucially, for research pursuing the establishment of cross-scale mechanistic correlations, comprehensive descriptors that span the materials tetrahedron remain indispensable. Incomplete and non-continuous adoption of data features may induce disruptions in cross-scale mechanistic correlation, undermining the interpretability of multiscale physical mechanisms and consequently the efficacy of material design strategies. Typically, effective checks of data feature comprehensiveness require domain-specific expertise and mechanistic insights into hidden structure–property relationships.

*The diversity and uniform distribution of data samples*. Applicability scopes and boundaries of research conclusions are largely determined by multispecies and evenly distributed data samples, yet most materials scientific datasets inherently carry biases, which may originate from human factors (e.g. researchers may preferentially select amine reactants in hydrothermal synthesis of organically templated metal oxides due to experiential familiarity and cognitive anchoring [55]), or experimental and/or computational resource constraints (e.g. computational power limitations skewing simulated property datasets disproportionately towards crystal structures with small unit cells). Also, negative data, mentioned in Section S2.1.1, represent a particularly insidious source of bias and demand specific scrutiny. While not mandated to be quantitively equivalent to the average of total data, they must constitute a proportion sufficient for statistical significance testing. Operationally, they can be detected through disproportionate clustering in high-performance regions, or abnormally narrow variance in target properties under similar experimental conditions, and determined by research objective and material system. If evidence of selective omission of failed or mediocre results is found, the dataset should be flagged as success-biased, even if the retained samples are individually accurate. Only class-diverse, uniformly distributed materials data prevent the interpretation of complex interplay of composition–structure–property interplays from being manifested as accidental phenomena in constrained scenarios. For a ML model specifically, such a dataset guarantees that the models are trained and validated on a distribution that faithfully reflects the true underlying materials space rather than an artificially sweetened subset.

## STRATEGIES FOR ACCELERATING THE DELIVERY OF HIGH-QUALITY DATA PRODUCTS

Stage-specific data quality requirements outlined in Section S2 serve as a foundational guide for delivering high-quality materials data. But undoubtedly, transformative impact relies on advanced tools and well-established infrastructures, which assist researchers to efficiently generate and manage materials data that meet the aforementioned criteria. They also enable the effective and sustainable implementation of full-cycle data quality control and optimization, which in turn, facilitates the translation of abstract conceptualizations into practical, sustainable research practices.

In this section, the three stages of the materials data lifecycle are further deconstructed into five fundamental activities, and tailored engineered strategies are proposed. Specifically, the globally integrated smart factory and the open and automatic pipeline correspond to the generation of new data and the collection of existing data, both ensuring data sufficiency and recording completeness requirements at the data acquisition stage. At the data management stage, the materials big data federation provides a unified and secure environment for multisource heterogeneous data, and ontology-based materials data governance enhances consistency and interoperability through semantic alignment and linkage construction. Finally, rather than elaborating on general strategies for materials data learning (readers may refer to the literature [56,57]), the rule-configurable automated tool for materials data quality assessment serves the data utilization stage. It could assist data users in rapidly identifying intrinsic dataset flaws while simultaneously providing quality feedback upstream to data producers, thereby fostering iterative improvement and elevation of materials data quality.

### Globally integrated smart factory for materials data production

Obtaining comprehensive, all-round descriptions of materials under rigorously unified environmental conditions represents the fundamental quality requirements at the acquisition stage and the shared aspiration of every materials scientist. Despite numerous HT infrastructures being established to accelerate the large-scale generation of materials data, the ‘cottage-industry’ production paradigm contributes to the absence of effective interaction and coordination mechanisms among these infrastructures, perpetuating structural deficiencies: (i) *Fragmented data production landscape*: attributed to different research priorities and functional limitations of laboratory equipment, data production units affiliated with distinct governmental, academic and industrial entities typically focus exclusively on specific length scales and materials systems or performance metrics, conducting experiments and computations independently. Despite being beneficial to parallel investigations on diverse materials dimensions and categories, such a siloed approach results in pronounced disparities in data coverage, creating an imbalanced data ecosystem where ‘data deserts’ co-exist with ‘data-rich zones’. (ii) *Inconsistent data documentation standards*: non-standardized environmental setups, inconsistencies in experimental design and data recording protocols, and variable emphasis on essential information collectively result in disparate levels of data documentation completeness and detail. While multiple research teams generate extensive property and performance data for the same materials across various scales, incomplete recording of environmental parameters confines these data to fragmented segments, precluding robust cross-scale modeling. (iii) *Disconnected computational–experimental workflows*: voluminous data from theoretical modeling and simulations remain inadequately utilized due to insufficient experimental validation, despite the investment of substantial computational resources. This failure thwarts the swift conversion of computational insights into experimental guidance, perpetuating unnecessary resource waste and low exploration efficiency, and undermining, rather than facilitating, rapid acquisition and standardized accumulation of property data.

**Figure 2.**
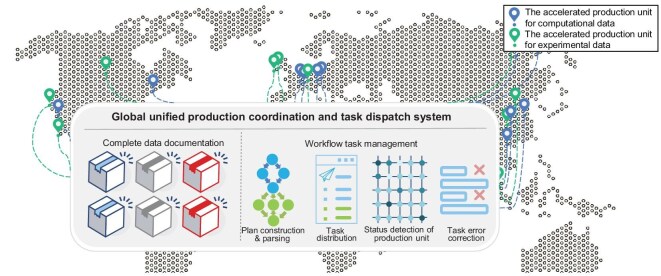
A globally integrated smart factory for materials data production, interlinking distributed accelerated production units via a smart production control system, facilitating coordinated execution and management of multi/cross-scale workflows, as well as the complete capture of data records.

To compensate for the functional deficits of individual laboratories, materials experimentalists have recently begun cloud-based collaboration with geographically distributed self-driving laboratories (SDLs) across multiple time zones. A representative example is the asynchronous cloud-based delocalized closed-loop strategy proposed by Strieth-Kalthoff *et al*. [58]. Such actions enable in-depth experimental interrogation of target material systems, and rapid and holistic acquisition of their property datasets. Meanwhile, this also inspires us: why not scale such an experimental collaboration network to encompass all experimental and computational nodes? This offers a potential remedy for the above summarized deficiencies in current materials data production practice. Accordingly, a globally integrated smart materials data factory (GI-SMDF), as illustrated in Fig. 2, naturally comes out.

By interconnecting materials data production infrastructures that are dispersed across various locations and designed for different materials categories and classes, a unified and coordinated data production factory emerges. Essentially, it transcends the inherent limitations of conventional single-institution approaches, enabling systematic exploration of multiple material types, and scalable fabrication of standardized data with comprehensive and complete parameter documentation. It also promotes the paradigm shift from isolated, protracted and localized research to a globally interconnected knowledge ecosystem. Geographically distributed, multidisciplinary teams collectively engage in rapid-iteration materials design, multiscale optimization and generation of high-value data. Another advantage of the GI-SMDF is that it optimizes worldwide configuration of academic resources, balancing the economic interests and scientific requirements for both infrastructure providers and users. For resource providers, the implementation of a reasonable pay-for-use mechanism effectively alleviates the financial burden of constructing and maintaining advanced computing clusters and automated labs. Meanwhile, without substantial upfront equipment investment, resource-constrained users can agilely pursue innovative studies on multiscale engineering and perform synthesis and characterization for complex multicomponent materials. The global-scale inclusive collaboration is therefore mutually beneficial for addressing complex challenges and advancing scientific and technological innovation in today’s interconnected world.

#### The actual data-fabrication workshop of the factory

HT computing platforms (e.g. Materials Project [28], AFLOW [29], EESMDP [59]) and automated laboratories (e.g. A-Lab [60], Ada [61], AMANDA [62]) are the important prerequisite for establishing data production factories. The ongoing digital transformation makes them increasingly remote-operable. Researchers are allowed to manipulate and deploy computational and/or experimental tasks via cloud-based tools (web browsers, apps and micro-services), without physical proximity to computing servers and/or laboratory equipment [25]. Such technical advantages also make it easier to connect each data production infrastructure.

As outlined in Section S2.1.2, exhaustively capturing and recording all parameters and outputs constitutes the basic requirements for these practical data production units. For fully digital simulations, it can be readily implemented. A representative example is AiiDA [63,64]. Via its unique provenance graph, complete computational history and details are systematically and automatically preserved, eliminating irrecoverable data loss during the simulation process and lack of traceability. In contrast, traditional physical materials laboratories struggle with complete collection and error-free documentation. Heterogeneous instrument interfaces and reliance on manual operations for some key steps often leave experimental records scattered across computers and paper notebooks. Excitingly, ESCALATE [65] presents a promising solution to this long-standing issue. It is a software package developed to standardize experimental protocols, automate data acquisition and streaming the reporting process, thereby simplifying primary data collection. Its efficacy has been preliminarily confirmed in metal-halide perovskite crystallization experiments.

Integrating ML techniques into these ‘data production workshops’ can further boost data generation efficiency [66,67]. However, the substantial efficiency gains must not come at the cost of physical plausibility or epistemic integrity. AI-generated data may carry intrinsic risks of producing outputs that violate fundamental physical laws or thermodynamic constraints, introducing systematic errors into downstream knowledge bases. Several strategies to mitigate these risks include: (i) selecting physically constrained ML models (e.g. those enforcing conservation laws, symmetry requirements, or discovered via symbolic regression) during the generation process; (ii) explicitly labelling the AI-generated data based on the degree of AI involvement (‘non-AI’, ‘partially AI’ or ‘fully AI-generated’); and (iii) subjecting AI-generated data to rigorous revalidation on linked computational and experimental platforms. Extended discussions on AI-accelerated materials simulations and experiments are presented in Sections S2.1 and S2.2.

#### Global unified production coordination and task dispatch system

To achieve collaborative control and efficient scheduling of computational and/or experimental tasks across distributed data production units, an intelligent production management system is indispensable. As both computational simulations and experimental procedures are sequential and repeatable, scientific workflows, formally described by directed acyclic graphs where vertices represent operational activities and edges denotes control and data dependencies, are ideal for planning and organizing these tasks [68,69]. The cross-domain experimental/computational workflow engine naturally becomes the central component of such an intelligent system.

Based on digital twin technologies, digital replicas of physical materials laboratories can be created. These digital counterparts enable electronic instruments to be perceived as workflow execution components by end users, facilitating experimental process reconfiguration and real-time interaction. Moreover, experimentalists can encapsulate routine operations into standardized, reusable modules, supporting flexible experimental workflows assembly. Likewise, theorists can package property-screening and prediction programs as modular components, with different configurable levels, ranging from fully disclosing algorithms and parameters to black-box, depending on project requirements and intellectual property considerations. The integration of computational and experimental components not only facilitates synergistic convergence between theoretical modeling and experimental approaches but, more importantly, provides the materials community with a platform where domain expertise and research protocols can be documented, transmitted and shared. Researchers doing follow-up studies can verify the accuracy and reliability of existing experimental/computational workflows and derive novel datasets through systematic replication of established protocols within equivalent computational environments or methodological frameworks. Moreover, they can focus on extending application boundaries of existing workflows by reusing and combining modular elements into innovative solutions for complex and challenging scientific problems, without being burdened by technical complexities of maintaining computing servers and large sophisticated instruments.

The incorporation of AI techniques further empowers such a system. Specifically, the reinforcement-learning-based task scheduler enables the precise orchestration and coordination of both computational and experimental workflows, optimizing the execution process and ensuring seamless simulation–experiment coupling. It could also intelligently allocate computing resources and appropriate experimental equipment according to task features and objectives. The deployment of AI agents even advances the factory towards a fully automated ‘unmanned data factory’. Ultimately, the reservation of standardized data communication interfaces facilitate both vertical and horizontal scalability of infrastructures, meeting the demands of emerging materials system development and improving the data production efficiency.

### The open and automatic pipeline for material data collection and extraction

Extracting data from publicly accessible scholarly documents and aggregating them into domain-specific thematic datasets with locally consistent structures represent another pathway for acquiring materials data.

The sufficiency requirements in the data acquisition stage necessitates the exhaustive aggregation and extraction of all relevant data, but in practice, this is not an easy task. Multisource documents exhibit heterogeneous styles, formats and grammar, precluding uniform data representation. Comprehensive consideration of physical format attributes and contextual semantic environment is required to guarantee the integrity and accuracy of extracted results. Traditionally, this task predominantly relies on manual operations.

However, the prohibitive labor costs and temporal bottlenecks of such a human-dependent approach impair efficiency when confronted with voluminous academic documents. Cognitive bias and fatigue also erode completeness and accuracy under data deluge. In the drive for expediency, researchers habitually prioritize harvesting the target data while neglecting to fully document metadata (including data source, extraction tool, parameter setting), which compromises the verifiability and reproducibility of extraction outputs.

Recent advancements like natural language processing (NLP) techniques and tools that are dedicated to materials text extraction [70–72] and image recognition [73–75] offer promising solutions for overcoming efficiency limitations, but most of them remain confined to single-modal data processing, incapable of holistic data extraction from compound documents. Extraction of associated metadata remains entirely user-dependent and conscious, with no effective hint or assistance. Crucially, the data extraction rules, designed for augmenting adaptability of those tools to materials domain-specific texts, are commonly scattered across research papers and institutional code repositories. This fragmentation hinders effective integration and formation of transferable and reusable rule systems, thus impeding the scalable extraction of homogeneous data categories.

**Figure 3.**
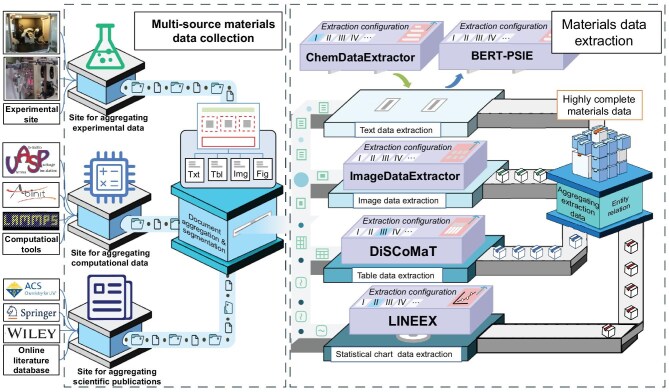
Architecture of the open and automatic pipeline for materials data collection and extraction. It comprises two main layers: the multisource materials data collection layer gathers unstructured data files and associated metadata from diverse heterogeneous sources (either actively pulled or passively received), then segments and categorizes different types of extraction tasks based on data representations; the materials data extraction layer executes these tasks via flexibly configured specialized extraction modules, and consolidates all results into a structured format.

To facilitate the diverse multimodal data-extraction requirements and reuse of extraction rules, while ensuring the automated and exhaustive capture of associated metadata, we put forward an open and extraction-rules-configurable pipeline. As illustrated in Fig. 3, there are two main layers in this pipeline: one for multisource materials data collection and another for data extraction.

#### Multisource materials document collection and segmentation

Autonomously harvesting raw data files from diverse sources in a timely fashion is the critical enabler that continuously triggers this extraction pipeline for parsing and extracting materials-specific information. Establishing robust connections to accessible data-source APIs (or via web-crawling tools and lightweight scripts) and proactively pulling raw data files thus constitute vital preconditions for effective pipeline operation. As raw simulation outputs (e.g. OUTCAR for VASP) or experimental records (e.g. XRD raw files) often demand additional parsing and structuring, direct integration with data ports of computational platforms or electronic experimental instruments is retained as a flexible, on-demand capability. During the collection process, both complete acquisition of raw data files and systematic recording of associated metadata are required. In the case of scientific publications, it entails not only the main text but also supporting information (if available), where, due to manuscript length constraints, essential methodological details and full output critical for research reproducibility are commonly archived. Metadata, including title, authors, source website and DOI, should also be captured and recorded as descriptive information by the data collection engine. These metadata are beneficial to enhancing credibility and interpretability of extracted results, and concurrently, enable registry-based caching of optimized services that further accelerate materials data processing and extraction process.

Another function of this layer is to identify data file types, generating data extraction tasks and routing them to corresponding processing subpipelines. Given the integration of various data sources, an intelligent agent with the capability of material-specific file type and category recognition is required. Generally, it should recognize common experimental outputs [e.g. transmission electron microscopy (TEM) images] and computational simulation results (e.g. log.lammps from LAMMPS). Specifically, LLM-based models (e.g. BatteryBERT [76]) can be leveraged for annotating each task with appropriate disciplinary-specific labels. An additional intelligent agent is needed for classification and distribution of extraction tasks. According to the differences among data representation methods, the raw data files could be deconstructed into four standard document entities (text, image, table, statistical charts) with segmentation techniques [77,78], thus generating different data extraction tasks. Finally, these tasks are dispatched to the specialized extraction pipelines based on their designated types.

Notably, although LLM-based agents and automation technologies enable full process automation in this layer, retaining the interfaces for human–machine interaction remains important. It ensures the power of automation is harnessed while researchers retain necessary oversight and control, refining and verifying results or filtering out non-compliant data files.

#### Flexibly extensible data extraction pipelines

In a typical PDF-based scholarly document, authors may employ diverse data representation methods, including textual narratives, graphs and tables, to articulate and substantiate the rigor of scientific claims. Comprehensively parsing and extracting all information is therefore essential, which would help others fully apprehend its scientific arguments and optimally reutilize the underlying data. However, to the best of our knowledge, there is currently no such powerful multimodal data extraction tool in the materials field. Combining multiple specialized single-modal data extraction tools accordingly becomes an alternative solution. At the data extraction layer, multiple parallel pipelines are deployed to handle different types of extraction tasks in synchronous or asynchronous ways, fulfilling the requirements for complete and efficient data extraction from complex raw data documents.

Usually, primarily due to the unique vernacular, syntactic structures, technical terminology and domain-specific semantics, general-purpose extraction tools fail to perform material-specific tasks well without customization. Thoroughly refactoring the extraction algorithmic framework and developing a dedicated extractor for precise content capture emerges as an intuitive engineering solution. For instance, the fundamental differences of physical characteristics between XRD diffractograms and scanning electron microscopy (SEM) images require differentiated feature extraction algorithms and dedicated tools. For detailed discussion on the classification, strengths and limitations of data extraction approaches tailored to different task types, please refer to Sections S3.1 and S3.2. Equipping each pipeline with standardized interfaces for ‘plug-and-play replacement’ of specialized extraction modules become necessary. The extractor can be viewed as a black box. Only by exposing the necessary interfaces, including inputting documents and extraction rules and outputting results, can it be effortlessly integrated into the corresponding pipeline. Retaining an extraction-rule input interface enables researchers to migrate the tool to new targets simply by modifying the rules. Commonly, these rules encompass not only the string matching patterns, but also the engineered prompts and instruction sets for guiding language models in target data extraction [79].

However, one persistent issue, as mentioned before, is that these rules are often tightly bound to research papers or even hidden. A shared, community-maintained extraction-rule repository represents the key solution to this problem. Rather than dispersing rules refinements across individual research groups, it enables global collaboration to optimize parsing methodologies and extraction algorithms. Validated improvements (e.g. overcoming boundary condition oversight when recognizing specific crystal structure images) can be immediately updated and synchronized repository-wide. Extraction rules would be universally applicable and precise, potentially outperforming human-conducted manual extraction in accuracy.

A centralized hub is connected to the end of all processing subpipelines. It organizes and consolidates the output generated by these pipelines into a structured format while aggregating all metadata from the collection and extraction processes, including names, version numbers and functional descriptions of the used tools and rules to improve completeness and repeatability of the final output. Regarding multiple extraction tasks derived from the same document, it is responsible for coordinated aggregation via an intelligent association and mapping engine or intelligent agent.

### Materials big data federation for promoting storage and circulation of multisource heterogeneous data

Data-quality enhancement is inherently iterative and long-term, necessitating repositories capable of securely hosting partially refined datasets. In the data-intensive era, a trusted data storage environment not only allows data owners to disclose their valuable data within the permitted scope, enhancing their willingness for sharing data, but also lays foundations for collaborative data quality optimization among multiple parties. Serving as a central nexus connecting data producers and consumers, it could even function as a ‘data exchange market’ when implemented with a data billing mechanism. This would continuously enrich the presently nascent high-quality materials data ecosystem, driving it towards robust abundance.

#### Heterogeneous material data storage

Data repositories and storage platforms are the essential infrastructure for incorporating materials data into research cycles and enabling progressive quality enhancement. Consequently, aggregating and archiving multisource heterogeneous materials data, regardless of quality status, constitute their core mission. Mainly driven by the Materials Genome Initiative [80] and similar engineering projects [81,82], numerous domain-specific data platforms [83–91] have emerged in materials science, and different storage strategies are adopted to facilitate systematic aggregation and unified management of multisource heterogenous data, thereby improving efficiency in data interaction and utilization. Three typical storage strategies, as well as their representative databases, pros and cons, are listed in Table 1. Essentially, these strategies reflect varying extreme degrees of trade-offs between data storage flexibility and platform management controllability; the former prioritizes flexible adaptation to dynamic evolution of data formats, while the latter emphasizes regulatory constraints to ensure data retrieval efficiency.

**Table 1.** Comparison of different heterogeneous data storage strategies.

Storage strategy	Representative databases	Advantages	Disadvantages
Schema-agnostic storage	MDF [83], Materials Commons [84,85], NIST Materials Data Repository [86]	(1) High compatibility: supports multiformat data submission, with no content modification (2) Low maintenance costs: simplified storage architecture, not needed for predefined data models	(1) Structural heterogeneity: difficulty in parsing across datasets, unable to harmonize governance in platform (2) Coarse data granularity: no direct access to fine-grained materials information within the dataset (3) Inefficient retrieval: datasets must be entirely downloaded to locate specific data points of interest
Platform-predefined storage schema	NOMAD [87], Citrination [88]	(1) High data consistency: unified storage model enforces normalization and improves structural compatibility (2) Efficient retrieval: structured storage supports fine-grained queries	(1) Low adaptability: rigid model is difficult to be compatible with new characterization techniques or non-standard data formats. (2) High maintenance costs: requiring continuous development/updating of parsing tools to accommodate data source changes (3) Integrity risk: information loss occurs ubiquitously when transforming context-dependent text (e.g. chemical reaction procedure) into structured form
User-defined storage schema	MDCS (now renamed as CDCS) [89], MGED [90], NMDMS [91]	(1) Flexibility: user-customizable templates to accommodate diverse heterogeneous data (2) Autonomy: decentralized schema definition rights and support dynamic data dimension extensions	(1) User burden: prestructured data required, increasing submission complexity (2) Alignment risk: vulnerability to metadata–data content mismatch in iterative updates (3) Version conflict: lacking explicit logging mechanism during static template updates, incurring inconsistencies between schema and archived data

**Figure 4.**
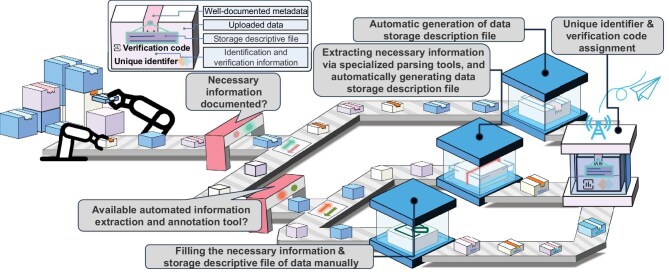
Illustration of the ingestion process for heterogeneous materials data.

Here, we propose a metadata-driven data storage and management framework (Fig. 4), a balanced approach for synergistic optimization of heterogeneous data compatibility and management efficiency. Its core component is the storage descriptive file (mainly encompasses general information, such as data schema and file type, and scientific information, such as data categories and comments). It could be generated by scanning and extracting multidimensional labels of dataset features with automatic tools like LLM-based agents according to accompanying complete metadata (stated in Section S2.1.2). In the case of insufficient or even absent associated metadata records, a corresponding metadata extractor (if available) and human intervention mechanism will be activated for supplementing essential storage descriptive data elements. Additional critical descriptive information, such as unique identifier, contributor profiles and tamper-proof digital fingerprints, will be dynamically allocated and automatically filled out. Following this process, the enriched storage descriptive file and its connected uploaded datasets will be encapsulated into an integrated package and then transmitted by secure data transfer tools, such as globus [92], to remote storage servers or platforms. More detailed description is presented in Section S4.1.

Throughout the whole process, all operations adhere to a non-invasive orchestration principle, ensuring that raw datasets remain immutable and bit-for-bit identical to their source. By employing external encapsulation—leveraging metadata sidecars rather than direct data transformation—the architecture preserves the native format and structural integrity of all uploaded assets. This format-agnostic approach provides plug-and-play compatibility, effortlessly accommodating diverse data schemas regardless of their scale, type or origin. Instead of developing brittle, dedicated parsers for every new format, system adaptability is achieved through the dynamic extension of descriptor manifests. This significantly minimizes technical overhead and reduces long-term maintenance costs associated with heterogeneous data environments. Furthermore, this strategy eliminates the risk of data corruption or information loss typically triggered by mandatory format conversions during ingestion, guaranteeing end-to-end consistency between the source and the archive. On the management side, the platform could decouple underlying storage from high-level administrative logic. By utilizing these storage descriptors, they can dynamically construct optimal archiving schemas and retrieval strategies. This architectural decoupling effectively shields upper-layer applications from raw data heterogeneity, enabling seamless, virtualized management of complex materials data.

Materials data platforms are the destination of these uploaded datasets. Various highly specialized platforms and comprehensive repositories, as listed in Section S4.2, have been established for categorized storage and shared access of diverse materials data [27]. While aiding in aggregation of trivial data locally, these highly fragmented databases, along with ambiguous profiling and potential long-term maintenance issues from unstable funding, substantially complicate cognitive and decision-making burdens on data contributors. Some materials data, due to the difficulty in identifying optimally suited databases, are frequently deposited either in (i) well-known generalist repositories that offer broad inclusivity but limited domain-specific alignment, or (ii) institutionally developed and maintained databases with high professional fit but constrained visibility. Worse still, some are released as journal paper supplements in CSV or other structured tabular files, failing to be wielded into standardized archiving systems, severely compromising data discoverability and reusability.

Here, we call for establishment of a community-wide interconnected materials big data federation. By integrating various domain-specific data platforms scattered across different geographical regions and organizations, nearly unlimited storage capacity and high fault tolerance for storing heterogeneous materials data can be achieved. Requirements that member databases must satisfy to join the federation are detailed in Section S4.3.

On top of the federation, intelligent routing decision algorithms can be implemented and integrated. By matching the categorical characteristics of uploaded data packages with the attributes of potential candidate databases, the system can autonomously determine the most suitable storage destination, eliminating manual decision-making complexity while improving the rationality of data storage. From the broader perspective, the federation could alleviate storage pressure on comprehensive databases. The implementation of unified integration protocols enables continuous and dynamic expansion of new storage nodes (including storage servers, databases, platforms), thereby enhancing the infrastructure capacity of the materials community to accommodate growing heterogeneous data. Logically, it could also support virtual data storage meshes partitioned by disciplinary dimensions, which achieves autonomous data management for specialized subfields within materials science and thus, ensures field-specific stewardship and cross-federation interoperability.

#### Blockchain-based material data transaction network

Ensuring secure data circulation both within and across communities is another responsibility of the materials big data federation. The generation procedure of materials data, strikingly unlike that of social data, demonstrates highly professional specificity, endowing itself with dual attributes: knowledge-intensiveness and technological exclusivity. Each experimental data point typically involves complex preparation process parameters, sophisticated equipment configurations and multiscale structure–property correlation analyses, encapsulating high-density intellectual investment and technical expertise. Nevertheless, the near-zero marginal cost of data replication and sharing practices poses challenges to the definition and protection of data intellectual property rights, with data plagiarism becoming increasingly prevalent. Furthermore, unsafe data access and acquisition mechanisms in data platforms substantially undermine the data contributors’ enthusiasm, resulting in the monopolization of high-fidelity datasets by a small number of research institutions.

Conventional data security strategies, including identity authentication and access rights assignment, seemingly meet the need of individual databases. However, excessive emphasis on external threats may lead to neglect of insider risks. Data are vulnerable to inadvertent or unintentional manipulations by internal authorized users, which might compromise data integrity and correctness, and lead to quality degradation. Granular control demands from diverse data contributors also fail to be satisfied. In cross-institutional academic collaborations, scholars require flexible, time-limited granting and revocation of access, retrieval and modification permissions. Conversely, industrial enterprises must preserve their commercially valuable findings as trade secrets to keep their competitive advantages. Besides this, hosting data in third-party databases means transferring fully security stewardship to platform administrators. While relieving custodial burdens for data owners, such practices may weaken their privacy rights like informed consent and autonomous control over data usage, sparking serious concerns over the erosion of data sovereignty.

From our point of view, blockchain technology, owing to its unique distributed ledger architecture, cryptographic security mechanisms and consensus algorithms, provides a promising solution for handling the critical issues in materials data circulation. An attribute-based encryption (ABE)-integrated dynamic access control model allows data contributors to flexibly configure multidimensional data access policies according to project needs [93,94]. Smart contract techniques in blockchain enable the encoding of data utilization rules into self-executing agreements, supporting threshold-triggered billing and citation-based dynamic pricing. The immutable recording of data-modification histories and ownership-transfer trajectories also provides critical certificates for preserving legal data rights. Adoption of the Practical Byzantine Fault Tolerance (PBFT) protocol [95] and its derivative algorithms [96] establishes the essential basis for secure and reliable data transactions within materials big-data federations. Detailed discussion is presented in Section S4.4.

Pioneering studies have preliminarily validated the technical feasibility of blockchain-based solutions in secure data storage and controlled sharing [97–100]. Notably, Wang *et al*. [98,99] proposed an ‘on-chain transaction, off-chain storage’ architecture that innovatively strikes a balance between blockchain storage efficiency and data integrity. By anchoring metadata hashes and access controls on the blockchain while storing large-scale materials datasets in a distributed file system, this approach inherits blockchain auditability while avoiding the performance bottlenecks associated with on-chain storage.

Operationally, the deployment of a blockchain-based materials data network represents a transition toward a decentralized data governance model. By providing data contributors with granular and verifiable control over their intellectual property, this infrastructure establishes a trustworthy circulation environment across geographically and institutionally diverse research communities. Such a framework provides the necessary ‘trust layer’ to operationalize a sustainable data economy, following the value chain of ‘data assetization → secure circulation → value realization.’ This model incentivizes high-quality data production by ensuring that every contribution is traceable, citable, and properly credited within the global materials science ecosystem.

### Ontology-based material data governance: boosting integrated data retrieval and interoperability

Structurally uniform data with semantic consistency and relevance are not only the fundamental prerequisites for individual studies, but also the driving force for retrieving cross-platform data efficiently and strengthening the community willingness to reuse open-access data resources. Given disparate domain-knowledge levels and inconsistent terminological conventions across research groups, accomplishing this with satisfactory quality is a non-trivial task. Several large-scale attempts in materials science [20,101,102] have sought to organize data indirectly via predefined storage schemas; their practical impact remains limited to local scopes. These schemas often result in implementation conflicts, with some even becoming obsolete over time, due to the lack of effective domain-wide support. It was investigated that currently, most data practitioners expend over 80% of their efforts in retrieving, accessing and curating task-relevant data [103], which severely impedes scientific productivity.

A primary reason for the limited adoption of these storage schemas and curated datasets is the absence of consensus-based governance paradigms, which could be addressed by ontological methodologies. Essentially, an ontology is a formalized expression for domain knowledge, which explicitly defines concepts, attributes, relationships and their axiomatic constraints that delineate permissible configurations and logical dependencies [104]. Specifically in materials science, ontologies could serve as a standardized and widely recognized terminology system that formally describes constituent material elements, including chemical composition, structure, properties and process, along with their hierarchical interdependencies. Functioning as normative framework, it can be helpful to systematically address semantic, syntactic and structural heterogeneity during data curation [51], transforming raw heterogeneous data into normalized, semantically coherent, highly interoperable assets. Technically, its hierarchical architecture and modular extensibility ensure exceptional adaptability, readily accommodating emerging concepts and curating data through incremental addition of classes, properties and axioms. Formal representation in logical languages (e.g. OWL [105] and Description Logics [106]) also empowers machines with comprehensive understanding capability of strictly defined disciplinary concepts and their associations. This enables automated integration of cross-platform heterogeneous data and the systematic construction of semantically interlinked materials data networks.

Here, the ontology-based data curation approach we propose mainly comprises three sequential steps, which progressively elevate both data quality and its scientific value. The currently available materials-related ontologies and discussion on their prospects are presented in Section S5.

#### Semantic alignment and data unification

Maybe divergent from common cognition, the unification of multisource heterogeneous data requires more than simple format normalization (e.g. normalizing ‘Li^+^’ and ‘Li ion’ to ‘Lithium ion’) and error spelling correction (e.g. rectifying ‘silcion’ to ‘silicon’). Deeper semantic analysis is needed. As previously stated, methodological and instrumental disparities in data production, combined with researchers’ subjective preferences in data documentation, give rise to two pervasive inconsistency forms in the materials data ecosystem: one single materials concept may be interpreted and characterized differently by divergent conceptual frameworks (e.g. graphite is considered from very different points of view in battery research and nuclear energy sectors); the identical terminological descriptors might carry different connotations when operationalized in different disciplinary contexts (e.g. ‘insulator’ has different meanings in the fields of research focusing on electronic conductivity and thermal conductivity). These implicit inconsistencies compel researchers to perform in-depth semantic parsing, leveraging ancillary metadata to achieve profound semantic harmonization and structural reorganization.

Materials-specific ontologies are uniquely effective at resolving this issue. By enforcing community-approved data definitions, representations and constraints, they eliminate semantic ambiguities in heterogeneous data while ensuring both accuracy and global semantic consistency with domain-specific terminological systems. For example, all density measurements can be uniformly defined as a float-type property with standardized unit kg/m^3^.

Generally, each terminological entry within the formal ontology is uniquely identified by the Internationalized Resource Identifier (IRI). This provides opportunities for virtual standardization. Regardless of their originating platforms or structural formats, the heterogeneous data, if semantically equivalent and all aligned with the same ontological term definition, can be dynamically bound with the same IRI. Standardized conversion protocols can be triggered when external data provision is required. This preserves platform-specific data labels, schema and formats in their native form, achieving maximum compatibility with existing data systems.

Further improvement lies in the integration of NLP techniques. By setting fuzzy similarity thresholds and performing semantic equivalence calculations based on various string similarity algorithms and metrics (e.g. cosine similarity, Levenstein edit distance, Jaccard similarity coefficient), it achieves accurate and efficient mapping of data entities to ontological concepts. Subsequent data homogenization and format transformation operations, when conducted on this basis, gain improved rationality. Furthermore, this approach could reduce the need for intense external interventions and the probability of human-induced errors during annotation, while keeping efficient processing capabilities for exponentially increasing volumes of heterogeneous data.

#### Semantic linkage construction

Rather than relying on ETL middleware for physical data replication and location migration, an ontology-based approach enables systematic integration and semantic inter-linking across decentralized materials data resources to be operated at the logical level. This obviates content modifications or structural changes to original datasets, effectively decoupling data semantics from detailed storage implementation.

In practice, the construction of ontology-guided data logical associations is often based on the global semantically aligned data frameworks and typically implemented in the form of subject–predicate–object triples. Within this representational approach, the subject and object refer to entity data (platform-specific unique identifiers or targeted links can be used to avoid altering data labels and content), and the predicate usually denotes the semantic inter-entity relationships, such as hasComposition and exhibitProperty. To enhance the understanding of relationship representation for both human and machine, URL links are advised to be introduced for supplementary description. For example, the microstructure characterization data of an alloy (hosted on Platform A) and corresponding mechanical performance test records (stored on Platform B) are logically related in both directions by the triples <Alloy X, hasMicrostructure, Structure Y> and <Structure Y, affectsProperty, Performance Z>.

Fundamentally, the ontology-guided logical association approach allows for flexible representation of intricate and multifaceted data relationships, exceeding the limitations of basic containment hierarchies in file storage systems and the implicit equivalence and reference connections characteristic of relational databases. Viewed through a different lens, the ontology can be engineered into a global graph-structured data network, alleviating researchers’ pains of locating relevant data in a chaotic swamp. The expanded searching scope of available data could also be realized by a unified semantic interface, providing a robust foundation for revealing the hidden associations among material genes.

#### Semantic reasoning and extension

Semantic reasoning, as a higher-level ontological application for data curation, aims to derive new data and, simultaneously, to seek and identify new relationships between data resources through axiomatic systems or logical rules. In ontologies, some concepts can be defined by other concepts using first-order description logic or a set of rules. Their values can be automatically inferred by the reasoning engine (including Pellet [107], FaCT++ [108]). For example, an ontology can automatically classify high-entropy alloys based on logical rules such as ‘material composition includes ≥5 principal elements with each element’s molar ratio between 5% and 35%’ [109], and simultaneously trigger associations with related concepts like ‘solid solution strengthening effect’ and ‘lattice distortion.’ This rule-based reasoning capability enables causal connection construction between previously isolated thermodynamic data (e.g. mixing entropy), structural parameters (e.g. XRD patterns) and property data (e.g. hardness).

With Semantic Web Rule Language (SWRL) rules, ontologies can also enable automatic computation of derived data or imputation of missing data. For instance, in the case where the electron mobility of a material is unavailable, the ontology-based system can estimate its value leveraging ontology-defined relationships between band gap, carrier concentration and mobility. This domain knowledge-based imputation approaches yields more physically plausible data completion results than those produced by traditional statistical imputation methods [110].

In addition, combined with graph algorithms (e.g. community detection [111], subgraph isomorphism and matching [112]), duplicate records or complementary data across datasets can be further identified. By systematically comparing the graph structure formed by materials compositions and properties, functionally analogous materials classes can be identified and then tagged, consequently expanding high-quality candidate space for materials design.

### The rule-configurable automated tool for material data quality assessment

The ontology-based curation approach effectively addresses structural inconsistencies and semantic conflicts, and establishes logical associations in multisource material datasets, thus generating strictly standard-compliant data collections and alleviating preprocessing workloads for data users. Yet it remains powerless against deeper quality issues, including incomplete features, imbalanced sample distributions and outliers. These issues still exist in data assets and may introduce reliability risks into data analysis or mathematical modeling. Accordingly, conducting multidimensional quality assessments before practical data utilization is necessary.

Given the continuously evolving nature of the field, as well as the significant reliance on domain-specific expertise, such practice is predominantly a labor-intensive, expert-driven process. Using statistical methodologies and visualization techniques, researchers make subjective judgments regarding data reliability drawing upon project specifications and empirical insight. Whilst enabling precise defect identification and maybe achieving optimal congruence between datasets and research objectives, it suffers from efficiency bottlenecks. More critically, opaque and non-uniform assessment metrics and algorithms frequently cast persistent doubt on assessment credibility.

The pressing demand for substantial high-quality data in the materials community renders the automated data quality assessment tool as a technological necessity. The complicated and varying application scenarios and quality assessment requirements place higher demands on it, requiring it to be adaptable and compatible with divergent evaluation standards and metrics.

**Figure 5.**
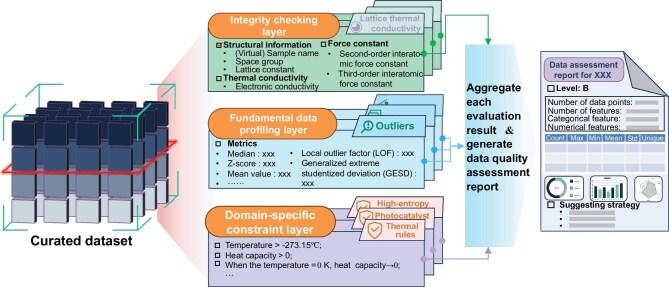
Overall schematic of the configurable and automated tool for materials data quality assessment. It employs a three-layer architecture: the integrity detection layer examines schema, column and population completeness of the dataset; the comprehensive feature assessment layer quantifies dataset quality through multiple dimensions (e.g. outlier, duplication and distribution uniformity); the domain-specific constraint violation layer identifies anomalous entries violating physical laws and chemical rules. A structured data quality assessment report is generated as its final output.

Here, we preliminarily devise a configurable and automated data quality assessment tool, the overall architecture of which is presented in Fig. 5.

*Integrity checking layer*. Checking the availability of (meta-)data entries against materials data documentation standards. In practice, it can be performed through three granular dimensions: schema, column and population completeness, with quantitative metrics, such as missing data ratio (missing data ratio: absent entries/total samples to present the results.

*Fundamental data profiling layer*. Detecting the global characteristics of datasets. Common assessment dimensions include outliers (specific quantitative metrics include standard deviation, interquartile range, Z-score etc.), duplicity (specific quantitative metrics include Levenshtein distance, affine gap distance [113], Jaro’s distance [114] etc.), class imbalance (specific quantitative metrics include imbalance ratio [115], imbalanced degree [116], likelihood ratio balanced degree [117]) and features comprehensiveness. Critically, these metrics can be extended to capture domain-specific patterns, such as compositional homogeneity or characteristic regions in phase diagrams, thereby ensuring relevance to materials contexts. Assessment of partial dimensions of data accuracy is also performed within this layer. Specifically, timeliness can be measured by examining metadata attributes (e.g. capture time, publication time, update time, validity period), and data authenticity can be assessed through various methods, including comparing data hash values with associated source signature, performing cross-validation of data points within the dataset against one or more independent, verifiably reliable sources, and confirming the identity, credentials and reputations of the data provider, along with data production processes.

*Domain-specific constraints layer*. Performing theoretical validation of data accuracy under the guidance of codified domain knowledge and mathematical expressions includes logical rationality (e.g. the bandgap energy value of promising solar cell materials should be in the range of 0.9–1.7 eV), numerical precision and validity of data labels. It addresses materials-data specificity by incorporating physics-informed rules and stoichiometric or thermodynamic checks, thus tailoring the assessment to materials-centric benchmarks.

The final output of this is a structured quality assessment report [118], which delivers both holistic quality-level rating [119] of the materials data and a detailed, multidimensional quality profile, including data integrity scores, missing-value heatmaps and a list of domain-specific rule violations. The overall quality level is computed based on the principle of equally weighted contributions from the aforementioned three layers. Such a balanced aggregation strategy ensures that the final rating reflects both standard compliance and intrinsic scientific merit equally. A dataset with sparse metadata but exceptionally high domain-specific accuracy and unique coverage would be flagged in the report as a high-potential candidate for metadata enrichment, not as a low-quality asset, and explicitly label metadata gaps as ‘documentation gaps’ rather than ‘data defects’. Appropriate suggestions for data usage (e.g. suitable for classification) and further data quality improvements can also be generated by powerful LLMs and provided alongside the report. With enriched diagnostic information, data practitioners are enabled to discover and comprehend potential quality issues in advance, reducing iterative data exploration.

Technically, quality assessment criteria within each architectural layer can be implemented in two ways: machine-readable, standardized configuration files (typically JSON, YAML, XML) encoding inspection rules, and domain-specific language (DSL)-based executable quality assessment models [120]. Both are eventually encapsulated as modular components specific to quality dimensions. Notably, the numerical quality metrics across these three layers are implemented as soft, percentile-based bounds, enabling flexible adaptation between strict physical limits and acceptable tolerances. Benefiting from these components, the tool can automatically conduct data quality assessment, while eliminating subjective bias introduced by human intervention. Quality assessment criteria could also be version-controlled and easily refined and expanded as knowledge evolves. Capitalizing on this, an open, community-maintained and -driven repository of configuration files that supports curated releases and pull-request-based contribution of newly validated domain knowledge could be established. Updated rules can be immediately validated by rerunning the tool on benchmark datasets, establishing a practical feedback loop for iterative refinement. More importantly, the transparent and uniform definition of data quality assessment metrics and rules guarantee reproducibility and comparability of the results, enhancing their credibility and fostering broad acceptance by the materials-research community. Furthermore, the emergence of such a tool enables quality supervisors to focus on designing quality inspection metrics, rather than on complete tool implementation. By means of configurable adaptation and scalable upgrades of the assessment components, the tools can meet diverse quality assessment demands across different material fields.

Nonetheless, four critical points call for special attention. (i) This tool is designed solely for data quality assessment and performs no modification to the original dataset, as alterations typically require deliberate consideration of intended application and domain-specific expertise. Hasty or uninformed changes may inadvertently degrade data quality. For instance, certain regions identified as anomalies may merely reflect insufficient sampling in the dataset. With additional data or an alternative analytical perspective, these data points could prove valuable, even yielding key insights in follow-up research. (ii) The data quality assessment report should be regarded not as a singular, static snapshot but as a dynamic and continuously evolving document. As the dataset changes, an up-to-date version of the assessment report should be correspondingly generated, maintaining continuous alignment with the evolving data landscape. (iii) The assessment report generated by this tool is intended to provide a standardized, baseline-comparable reference for data quality. In exceptional or complex cases, in-depth and meticulous evaluation by domain experts remains an indispensable step to ensure the reliability of the final judgment. (iv) The assessment report is designed as a decision-support tool, not a decision-making substitute. Its purpose is to provide an objective, multidimensional quality profile and give generalized applicability notes. Researchers could then use it to apply their domain knowledge and autonomously determine whether and how the dataset aligns with their scientific objectives. Specialized usage recommendations are not provided, as the actual use cases of the data cannot be predetermined.

To briefly summarize, the automated data quality assessment tool constitutes more than an important infrastructural part for materials informatics, but also acts as a catalyst for advancing quality metric formalization and, in the long term, the establishment of a data quality library across materials subfields. It aids researchers in identifying early indicators of prediction uncertainties caused by inadequate data quality and drives targeted quality-enhancement measures.

## CONCLUDING DISCUSSION

Well-curated, reliable and interoperable datasets preserve hard-won knowledge as enduring scientific assets. They form trustworthy foundations for theory development, supporting research both through direct data-driven inquiry and ML-augmented discovery. In this study, focusing on the materials data journey throughout the research lifecycle, we identify and define stage-specific quality control requirements that determine its intrinsic scientific value and readiness for diverse purposes. A global quality control framework is thus established, and potential data quality issues can thus be detected and rectified at the earliest stages by preventive governance strategies. On top of that, five community-level strategic initiatives are outlined to render the framework operational and to foster sustained and efficient production of high-quality materials data. It must be noted that these strategies are intended as adaptable guides rather than rigid prescriptions, as they may require further refinement through practice.

Looking ahead, this framework is expected to evolve into a community-maintained specification that integrates seamlessly with emerging AI-driven research pipelines. Such evolution will institutionalize end-to-end data quality governance and accelerate the construction of a sustainable high-quality materials data ecosystem. The establishment of domain-specific standards would be the catalyst for this vision, maximizing the synergies between the proposed initiatives.

As an essential part of materials informatics, data standards do more than ensure the capture of critical information during production; they provide the scaffold for structured, cross-disciplinary exchange and establish robust criteria for quality evaluation. Despite these advantages, key subdisciplines within materials science still lack standardized practices.

In our opinion, drafting and implementing materials data standards should follow the sequential workflow of ‘controlled-vocabulary construction, domain-ontology modelling and community-repository registration’, while conforming to the ‘core constraints with domain extension’ principle. Atoms, molecules and crystal-structure information, together with intrinsic properties, represent the fundamental determinants of macroscopic behavior and the central thread throughout the entire materials research lifecycle. They must therefore receive top-priority standardization to ensure cross-laboratory comparability and integrability. Domain-specific extensions, on the other hand, should remain flexible, allowing each subdiscipline to augment those core elements according to its characteristic parameters and performance metrics. Such a hierarchical principle preserves disciplinary knowledge integrity while avoiding over-standardization, thereby maximizing reuse of existing standards and high-value datasets.

We remain convinced that collaborative efforts across research groups will yield an influx of domain-specific standards and, consequently, a wealth of high-quality data. In the future, nearly all scientific content will be universally standardized, enabling scientific operations to be streamlined into automated, highly efficient pipelines, and that will empower human scientists to fully harness their imagination and creativity, focusing on decoding the ultimate mysteries of materials while leaving the labor-intensive data orchestration to automated systems.

## Supplementary Material

nwag108_Supplemental_File
