# Supplementary material for: Towards a sustainable high-quality materials data ecosystem: frameworks and strategies
Source: Natl Sci Rev. 2026 Feb 14;13(7):nwag108. doi: 10.1093/nsr/nwag108 (PMC13105177; doi:10.1093/nsr/nwag108)
Supplement: nwag108_Supplemental_File [file nwag108_supplemental_file.pdf]

Supplementary Information

Comprehensive Requirements and Strategies toward Constructing High-quality Materials Data Ecosystem

Bing He[1], Kaixuan Wang[1], Zhou Jiang[1], Yueyu Zhang[2],

Maxim Avdeev[3], Siqi Shi[2,4,*]

[1]*State Key Laboratory of Materials for Advanced Nuclear Energy & School of Computer Engineering and Science, Shanghai University, Shanghai 200444, China*

[2]*State Key Laboratory of Materials for Advanced Nuclear Energy & School of Materials Science and Engineering, Shanghai University, Shanghai 200444, China*

[3]*School of Chemistry, University of Sydney, Sydney, NSW 2006, Australia*

[4]*Materials Genome Institute, Shanghai University, Shanghai 200444, China*

[*]E-mail: sqshi@shu.edu.cn (Siqi Shi)

content

S1 Metadata framework for complete metadata documentation ... 3

S2 Critical components of globally integrated smart factory for materials data production ... 3

2.1 Accelerated production unit for materials computations ... 3

2.2 Accelerated production unit for materials experiments ... 9

2.3 Intelligent system for production management ... 18

S3 Constructing the open and automatic pipeline for materials data collection and extraction ... 23

3.1 Methods overview for developing materials data extraction modules ... 23

3.2 Future development direction ... 29

S4 Constructing materials big data federation ... 30

4.1 Detailed process for ingesting heterogenous materials data ... 30

4.2 Existing materials databases ... 32

4.3 Entry Requirements for Materials Big Data Federations ... 42

4.4 Blockchain-based Data Security ... 43

S5 Benefits, barriers and potential solutions for promoting ontology-driven data curation 44

5.1 Scarcity of domain-specific ontology 45

5.2 Insufficient ontology application for data curation practices 47

5.3 Deficient ontological axiom formulation 48

S6 Current practices in promoting data standardization in materials science 50

References 59

S1 Metadata framework for complete metadata documentation

- ***Descriptive metadata***: structured information that provides a comprehensive overview of data content, features and attributes. Core elements of this metadata typically encompass basic information (e.g., unique identifier, data categories, annotations), temporal and spatial information (e.g., time and geospatial coordinates of data generation), and responsible parties (e.g., owner, affiliated institution). Similar to abstracts in scholarly publications, the concise and well-organized descriptive metadata could enable efficient identification and comprehension of data content, thus allowing researchers to swiftly determine the relevance to

their research topic. In practice, it is advised to adopt the standardized schemas, such as Dublin Core [1], to realize the normative documentation of this metadata type.

- ***Provenance metadata***: Brief records about the entities, activities, and personnels involved in the production, modification, transformation of the resultant data [2,3]. It can be visually expressed through concise directed graphs [4] and Sankey diagram [5]. It involves the chronological sequences of data generation, modification and other critical processing activities, alongside executing agents and operational purpose of each activity. Documenting the provenance metadata has various advantages: (1) supporting re-production and authenticity validation [6], thus enhancing the credibility of data; (2) providing foundations for building data lineages; (3) facilitating diagnostic tracing of data anomalies and experimental failures while enabling procedural optimization to enhance production efficiency; (4) beneficial to establishing preservation mechanism of data ownership and copyright. Currently, several provenance metadata specifications have emerged, including Open Provenance Model (OPM) [7] and the PROV Family of Documents [8].
- ***Technical metadata***: Supplementary information of provenance metadata, detailing all parameters of each data processing activity. As an example, in materials computational simulations, technical metadata may include the utilized tool's name, parameters settings, and environmental configurations. Since single data processing activity may be performed by multiple steps, one piece of provenance metadata could correspond to multiple technical metadata records. Systematically integrating of provenance metadata with parameterized technical details enables precise reconstruction of data processing workflow and obtain data products closely match the original [9], thereby responding effectively to reproducibility crisis of scientific discoveries. The separation of technical metadata from provenance metadata has two main advantages: (1) reducing the cognitive load of scientific practitioners when dealing with complex provenance chain; (2) enabling fine-grained access control to protect core parameters related to commercial secrets or patented technologies, while transparently disclosing provenance metadata to be in line with open science principles, thus achieving an "organic balance" between safeguarding scientific research integrity and maintaining commercial interests.
- ***Structural metadata***: Information describing how data is organized, structured and represented, such as the meaning, type, range of values, representation method of each data column and revealing the hierarchies, relationships and linkages between data entries. Structural metadata can aid both internal and external researchers in accurately comprehending and utilizing data, while also enabling data platforms to deploy ef-

ficient physical storage strategies and logical navigation capabilities. For tabular data, the CSVW specification provides a W3C-standardized framework that formalizes structural metadata entries.

- ***Preservative metadata***: Constituting important information for long-term preservation and accessing policies of digital data resources to ensure longevity, accessibility and integrity. Specifically, it includes records of storage approaches (e.g., file formats, encoding methods, and storage locations) and data access policies (e.g., recommended data access software, access permissions, and embargo periods). The meticulously recording of preservative metadata facilitates the rapid formulation of effective preservation strategies for digital objects within repositories, ensuring compliant data utilization. Both Preservation Metadata Maintenance (PREMIS) [10], a reference specification for preservation metadata, and Creative Commons Rights Expression Language (CCREL) [11], a formal framework for representing permissions, could be helpful to normatively recording this metadata.

S2 Critical components of globally integrated smart factory for materials data production

2.1 Accelerated production unit for materials computations

2.1.1 Role of computational simulations in advancing materials research

Materials simulation constitutes a computational approach that, based on and integratively combining fundamental physical principles, chemical theories, and numerical analysis methods, mathematically represents the microscopic mechanisms and macroscopic behaviors of materials. This methodology enables the prediction of functional properties and the virtual screening of candidate materials. Practically, the simulation approaches require judicious selection and systematical modification as the distinct physical mechanisms dictating materials properties vary fundamentally across different temporal and spatial scales. For instance, quantum or classical mechanical approaches, such as Density Functional Theory [12–14] (DFT), Molecular Dynamics [15,16] (MD), and sampling methods like Monte Carlo [17,18] (MC), are commonly employed for analyzing the atomic-level materials problems, including electronic structure evolution and interatomic interactions. In contrast, phase-field simulation techniques [19,20] are utilized to investigate mesoscale phenomena, encompassing micro-structural evolution and formation, phase interface migration process.

Diverging from the empirically constrained methodologies of conventional material research character-

ized by labor-intensive and pain-staking iterative experimental cycles, computational simulation methodologies, by virtual of their fully numerical nature, enable efficient high-throughput properties prediction and virtual screening of thermodynamically stable materials configurations with desired functional attributes through automated parallelization of algorithmic workflows across high-performance computing (HPC) clusters. Computational simulations, characterized by their efficiency, accuracy, and interpretability of results, have proven to be a reliable approach for the rapid generation and expansion of material-gene databases as well as the construction of high-dimensional phase diagrams. Furthermore, they offer well-established complementary insights that guide experimental research in the design and development of novel materials, thereby minimizing the unnecessary expenditure of experimental resources. Recently, computational simulation methods have shown unparalleled capabilities in high-throughput screening and property prediction across diverse materials systems, including nanoporous materials [21,22] , alloys [23,24], ceramics [25,26].

While transformative, material simulations are not panaceas. The persistent constraint of precision-efficiency tradeoffs in conventional simulations techniques, as well as unresolved multi-scale data integration, continues to impede in-depth materials investigations. Fortunately, the advent of artificial intelligence and its integration with computational frameworks offer an promising solution to these issues, fundamentally reshaping the underlying logic of computational modeling and catalyzing transformative innovation in computational materials science [27,28].

2.1.2 Explicit acceleration of computational simulations via artificial intelligence

Re-engineering computational workflows in complex multi-scale simulation system stands for a pivotal frontier for AI-assisted and accelerated computation. In the field of computational materials science, it is widely recognized that classical molecular dynamics simulations provide computational feasibility for macroscopic studies involving ensemble sizes surpassing 10^5 atomic scales and temporal resolutions on the order of microseconds. However, these simulations are inherently limited by the reliance on empirically parameterized interatomic potentials, which restrict their predictive accuracy when applied to complex material systems. Conversely, DFT-based first-principles simulations benefit from well-established pseudopotential libraries for quantum mechanical accuracy. However, their computational demands inherent in eigenvalue calculations restrict practical applications to picosecond temporal regimes and materials system below 10^3 atoms, precluding practical investigations of extended temporal and spatial scales in complex systems. Prohibitive computational costs and time demands substantially make it difficult to efficiently perform property prediction and

screening of materials with large-scale atomic systems. Under this circumstance, introducing domain-specific ML models with pattern recognition and inference capabilities into computational frameworks might be beneficial to achieve concurrent optimization of quantum-level accuracy, computational efficiency and multi-scale resolution in materials modeling [29,30]. ML models can be used to, on one hand, replace computationally intensive steps in material simulations [31–34], such as solving the eigenvalues of many-body Schrödinger equation, thereby significantly improving efficiency and maintaining or even augmenting precision. For instance, Das [33] *et al*. ingeniously utilize multilayer neural networks that trained on the precise output data of quantum many-body calculation to fit the exchange-correlation functional part within DFT calculations. This approach enables high-precision determination of ground-state energies for magnesium-based alloys, substantially reducing computational time while preserving the accuracy characteristic of quantum many-body methods. On the other hand, ML models can be directly employed to predict materials properties, such as atomic density and forces, molecular energies, thereby bypassing the computationally intensive process in DFT calculations (including basis set expansion, Hamiltonian matrix construction, and self-consistent field iterations) [35–37]. Notably, although the development of these ML models is not an easy task, which demands sophisticated hyperparameter optimization and iterative refinements of algorithmic architecture, alone with substantial consumption of computational resources, such process is essentially a one-time investment with remarkable long-term benefits. Several studies have shown that well-trained ML models could achieve millisecond-level computation and materials properties prediction, greatly expediting generation of multi-scale materials datasets [35].

In addition to improving internal computational procedures, the accelerated impact of materials simulation driven by machine learning models is evident in the intelligent exploration of multi-parameter spaces to identify optimal configurations. Concurrently, this approach enhances simulation accuracy and expands the scope of candidate material exploration. Traditional computational simulation methods rely heavily on hand-crafted parameterization and heuristics approximation to construct surrogate models of the physical world, which could be constrained by insufficient understanding of coupling mechanisms of multi-physical fields in complex systems. Alternatively, ML models can, based on historical parameter sets and reasonable convergence criteria or performance thresholds, autonomously and rapidly achieve systematic refinement of key parameters, such as interatomic potential energy surfaces and electronic structures, significantly enhancing computational efficiency while maintaining the data accuracy comparable to or surpassing that of convention-

al simulations. Moreover, the integration of generative ML models in computational material science [38,39], such as MatterGen [40], MatterGPT [41] and CGWGAN [42], liberates these computational simulation methods, particularly the first-principles simulations, from its historical dependence on experimentally observed structures, thereby enabling systematic exploration of diverse complex and novel materials systems and extending the simulation boundaries beyond conventional materials space [38].

Despite these advantages, it cannot be ignored that the inherently opaque decision-making process of ML models precludes systematic elucidation of constituent input variables' causal contributions for the output. The non-transparency generation mechanisms often engenders skepticism within the scientific community regarding methodological rigor of research findings and resultant data, which in turn sparking critical debates about concerns about the looming reproducibility crisis in AI-driven scientific inquiry [43,44]. More critically, the ML-generated materials datasets pose risk of epistemic contamination or even degradation of established knowledge systems when utilized without uncritical consideration. Yet the proliferation of pseudo-physical output data, which often fails short of strict adherence to physical laws or thermodynamic principles, necessitates resource-intensive cross-verification against simulation and experimental benchmarks, a formidable challenge in the verification process. Response to this issue, embedding domain-knowledge into ML powered data generation process become imperative. Symbolic regression [45–47] present a viable technique for achieving this. Stringent verification methods are also needed, as they are helpful to improve the understandability and interpretability of AI-generated data [48], thereby fully unleashing the power of ML models in successful materials discoveries. Furthermore, it is strongly recommended that differentially labeling to the materials data according to the degree of AI involvement in its generation process. For instance, data obtained through traditional computational methods could be tagged as "non-AI-engagement", while "partially-AI-engagement" and "totally-AI-engagement" could denote new data produced through intermediate and comprehensive AI participation, respectively.

Whilst AI-generated materials data may not be endowed with fully epistemic certainty, it retains heuristic potential to stimulate conceptual exploration in materials research [49].

2.1.3 Implicit acceleration of computational simulations via artificial intelligence

The impact of AI on accelerating computational materials science is comprehensive and multi-faceted, revolutionizing both methodological approaches and infrastructural frameworks. Artificial intelligence can explicitly improve simulation efficiency through the reconstruction of simulation architectures, optimization

of parameter selection, and extension of the scope of simulated material systems. Furthermore, AI can be integrally incorporated into high-throughput computational infrastructures. Such practice could improve utilization rate of computational resources and boost overall computational efficiency, thereby accelerating the generation of material simulation data through intelligent task orchestration and dynamic resource allocation within distributed computation environments.

The high-throughput computing infrastructures, including Atomate [50], Fireworks [51], AiiDA [52,53], MatCloud [54], has been empirically demonstrated to serve as a robust enabler for swift advancement of computational materials science. They effectively eliminate the reliance on human intervention for configuring the underlying computing servers, scheduling tasks, tracking and recording workflow provenance, enabling computational scientists to concentrate on designing project-specific workflows through integrated modular simulation programs and software. Not merely propels the role change of materials scientists from full-time computational operators to architect of simulation process, they, more crucially, provide the technical support for in-depth exploration of unknown materials systems and their emergent properties. However, severe dual bottlenecks in computing power and efficiency emerge when performing high-fidelity multi-scale simulations of ever-expanding atomic systems.

Horizontal scaling of computing servers, coupled with optimization of existing computational resources including deployment of GPU-accelerated tensor processing units and even integration of edge computing nodes, represents a straightforward and intuitive strategic approach to address these challenges. Nevertheless, the heterogeneous nature of these computing resources may introduce considerable disparities in architectural systems, task compatibility and inter-node communication latencies. For instance, in heterogeneous computing environments, architectural discrepancies in floating-point processing units across compute nodes may lead to numerical precision mismatches when aggregating computational results across nodes. Moreover, as scientific workflows gain increasing adoption across research communities, their growing complexity necessitates advanced workflow management mechanisms within high-throughput computing infrastructures to efficiently orchestrate workloads comprising tens of thousands of computational tasks, the execution times of which span from milliseconds to hours. However, existing high-throughput computational infrastructures predominantly employ predefined rule-based task scheduling strategies, where computing resource allocations mechanisms simply rely on task priority queues, resource requirement template matching, or submission order. Owing to the inability to accurately assess the compatibility between task characteristics and resource attrib-

utes during the task allocation process, these systems struggle to dynamically assigning computing tasks to appropriate computing nodes. This limitation may lead to secondary issues, such as unanticipated task termination or errors related to architectural incompatibility.

Excitingly, the emergent AI-powered dynamic task scheduling systems holds the promise of solving these problems [55]. By autonomously exploring state-action-reward landscapes and subsequently dynamically optimizing variable resource availability alongside fluctuating energy-efficiency constraints through policy gradient iterations, reinforcement learning-based scheduling frameworks [56] can achieve workflow equilibrium states. These states maximize computational throughput while sustaining near-optimal resource utilization ratios within stochastic computing environments. Graph Neural Network (GNN)-based dependency-aware analytical frameworks could automatically resolve the topology of tasks in complex workflows, thereby enabling in-situ adaptive reconfiguration of execution pathways via gradient-driven structural optimization. Besides, AI techniques enable anticipatory computational trajectory analysis, preemptively detecting abnormal execution branches in the workflow to mitigate redundant computational expenditures. Furthermore, the systematic archiving of computational datasets coupled with cross-domain knowledge transfer techniques enables efficient re-purposing of historical simulation records. Advancing this, the simulation cycle times could be accelerated by establishing feature-temporal cost correlation models within materials property hyperspaces.

2.1.4 Prospects for future development

In the future, the strategic convergence and synergic innovation of AI techniques and high-throughput computing architectures are poised to drive the emergence of next-generation intelligent computation platforms. These advanced platforms are anticipated to integrate multiple functions, including autonomous physical-aware modelling, intelligent computing and data mining and analysis. Basically, the implementation of iterable workflow architectures that follow modular design principles endow computational platforms with the ability to flexibly adapt various computing tasks, thus better meeting the ever-changing demands during materials discovery. There, fundamental computing software (e.g., VASP for first-principle calculations and LAMMPS for molecular dynamics) and materials informatics tools (e.g., PyMatgen [57], Matminer [58]) are packaged and provided as containerized components with normalized inputs and outputs. Workflow engines are employed to execute workflows assembled by these components according to specific topological dependencies. This approach enables the automated integration of simulations across multiple scales, from electronic to macroscale levels, through the implementation of event-driven scheduling protocols. Advancing this,

the systematic integration of large language models (LLMs) within computational platforms enables researchers to define simulation parameters—such as processing protocols, parametric constraints, data tracking criteria, and boundary conditions—via natural language conversational interfaces. These inputs are then systematically converted into computational instructions through semantic parsing methods, subsequently transformed into executable computational graphs, and ultimately autonomously compiled into workflows. This process effectively eliminates the necessity for manual scripting and configuration.

A more pioneering frontier might be the self-evolving computing system. By integrating context-aware decision mechanisms with autonomous feedback loops, the computational workflows and associating computing parameters of each activity could be continuously refined and optimized, and a self-evolving 'modeling-computing-analyzing-optimizing' cycle is thus effectively established. The autonomous iterative search capability in high-dimensional materials design space brought by such system overcomes the dimensional reduction constraints inherent in human-centric experimental design paradigm, enabling efficient navigation of high-dimensional material design spaces and facilitating the discovery of previously unidentified high-performance material candidates alongside their corresponding multi-parametric datasets. Additionally, the development of open standardized workflow protocols [59,60], along with specialized technical infrastructure [61,62], such as curated workflow registries and repositories [63], could enable computational simulation processes to be shared and reused within the wider materials community, rather than being restricted to specific research groups, thereby facilitating the unprecedented emergence of data capable of explaining complex material phenomena.

2.2 Accelerated production unit for materials experiments

2.2.1 Role of experiments in advancing materials research

Materials simulations, which involve the formulation of the complex mechanisms underlying material systems into mathematical models with key tunable and adjustable parameters (such as lattice constants and electronic correlation energies), enable materials scientists to pre-screen and exclude virtual materials that contravene fundamental physical laws and chemical principles. This process substantially shrinks the search space of candidate materials and concurrently facilitates a more focused exploration of optimal materials with the potential to exhibit desired functional properties, thereby improving the efficiency of materials design and

development.

Nonetheless, this approach exhibits inherent constraints in clarifying structure-property correlations for emerging material systems with underdeveloped theoretical frameworks. Apart from this, the oversimplified materials models, idealized configurational assumptions (e.g., the micro-structural defect concentrations) and insufficiently practical experimental constraints [64], lead to cognitive gap between theoretical predictions and empirical validation. It means that the material properties are not typically directly equivalent to the final engineering property of interest. For example, the ground state approximation, commonly used in computational models, is often predicted on 0 k temperature assumptions, perfect crystal structures, and neglect of surface defects. Consequently, this approximation may exhibit systematic discrepancies when compared to actual synthesis conditions, which are characterized by elevated temperatures/pressures, non-equilibrium kinetic processes, complex interfacial phenomena. This misalignment creates a serious mismatch between experimental and calculated data. It has been reported that 80% of the convex hull formation energies in inorganic crystal structure database (ICSD) [65–67] are higher than those predicted by DFT [68].

Accordingly, experimentation has long served as the cornerstone of materials science and holds unique dual position in materials innovation. It is the culminating stage in forward materials design, determining whether the theoretically designed materials can be successfully synthesized and whether their experimentally measured properties align with computational predictions. At the same time, it is the starting point in materials reverse-engineering, where the performance refinement objectives of existing materials are proposed.

2.2.2 Automated experimental laboratory

Unlike simulation methods that can be fully automated in virtual environments with precisely tunable parameters, material experimentations typically involve greater complexity and variability, encompassing numerous uncertainties and difficult-to-control factors throughout their execution. When computational errors or output results that deviated from expectations occur in a certain step of computational simulation workflow, researchers could attempt to achieve process rectification by rolling back to preceding cached nodes and re-executing the corresponding step. But this does not apply well to materials experimentation. The materials sample is experimentally synthesized through multi-step procedures, followed by multi-dimensional physicochemical characterization process to comprehensively measure its properties. Given that irreversible structural transformation of the original samples during experimentation, any experimental failure means restarting the entire process from scratch. No intermediate checkpoint exists. These inherent natural characterized render

materials experimentation as a scientifically rigorous yet resource-intensive methodology, marked by high economic investment, substantial time consumption and comparatively limited productive output. For example, the traditional "heat and beat" methods for synthesizing solid-state organic materials requires extended periods from days to months to reach thermodynamic equilibrium before conducting crystallographic microprobe analysis for structural and property characterization [69]. More crucially, the experimental procedure is subject to various potentially stochastic perturbations, such as inadvertent human operational errors, subtle environmental fluctuations, and instrument degradation due to wear and tear. All of these factors make materials experiments, particularly sample preparation, not only difficult to be strictly replicated but also prone to data distortion and even invalidation. Additionally, paper-based experimental documentation is susceptible to both transcriptional omissions and cognitive biases of researchers, potentially leading to the exclusion or misrepresentation of critical experimental parameters and results. These systematic shortcomings of traditional experiments call for technological innovations in materials laboratories, driving the implementation of automated materials experimental research infrastructure.

Benefiting from the decreasing procurement cost of hardware components (including industrial robots and sensors), as well as concurrent advancements in supporting technological frameworks [70] (including remote communications, automated programming, and the Internet of Things networks), modern material experimental laboratories are integrating various automated equipment and devices, aiming to assisting and progressively substituting laborious operations in sophisticated sample synthesis and performance characterization process [71–74]. The continuous improvement of automation within physical material laboratories plays a crucial role in advancing materials research. Firstly, the implementation of automated laboratory has demonstrated transformative potential in expediting the experimental cycles, achieving unprecedented temporal efficiency gains for empirical data (2–5-fold temporal efficiency gains compared to traditional experimental workflows [75,76]) and enabling systematic validation of theoretically designed materials across diverse processing and synthesis conditions [77]. The automated experimental workflow developed by Li *et al*. [78] is capable of completing the synthesization of 8,172 metal halide perovskite single crystals via inverse temperature crystallization method in 400 hours, ~93% time reduction (from 6,000 hours to 400 hours) compared to the traditional manual operation (10 reactions per day). Additionally, the introduction of automated experimental instrument into materials laboratory empowers researchers with precise regulation capabilities of experimental parameters, minimizing inference from operator-dependent anthropogenic variability and envi-

ronmental fluctuations [73] , thereby providing a foundational support for the construction of reproducible, standardized, and automated experimental workflows. Besides, the entire automation of experimental process, encompassing all operations from sample preparation to advanced characterization, will facilitate the generation of high-fidelity, well-documented datasets with exceptional consistency and methodological rigor, while guaranteeing the process traceability, results data reliability, and the cross-platform experimental reproducibility through strict experimental protocols.

2.2.3 Autonomous experimental laboratory

While the automated laboratories hold the potential for improving the production rate of large quantities of high-fidelity data by substituting labor-intensive and highly repetitive experimental operations, substantial human intervention remains indispensable for coordinating interactions between heterogeneous equipment and managing the experimental data flow transmission. What's more, each materials experiment mandates researchers to preset parameters and adjust them promptly based on experimental feedback. These shortcomings render automated laboratories fundamentally mere extensions of human-operation processes, with only a minimal reduction in the duration of experimental research cycles. When confronted with the combinatorial explosion challenges inherent in high-dimensional composition spaces comprising multiple chemical elements and physicochemical property parameters, it still relies on grid-based searching strategies guided by human intuition and empirical knowledge [79,80], unable to achieve rapid sampling of specific material systems and exhaustive multi-objective in-depth analysis with synergistic optimization [76]. It is particularly true when target properties are interdependent or even conflicting. This can be exemplified by the design of alloy materials with both high-temperature resistance and high strength. The quintessential multivariate optimization dilemma lies in achieving thermodynamic equilibrium between lattice distortion energy and dislocation migration barriers, while maintaining crystallographic stability under extreme operational conditions. These limitations leave evolutionary room for the further advancement and upgrading of automated laboratories.

Excitingly, big data-driven AI technologies are supplanting human scientists as the control center for monitoring and coordinating all experimental workflows, triggering a revolutionary transition from automated laboratories to intelligent ones.

The self-driving Laboratory (SDL) [81], also termed materials acceleration platforms [82], intelligent laboratory [83], or autonomous experimentation systems [84] , is broadly recognized as the cutting-edge mani-

festation of next-generation materials research infrastructure [75,81,82,84–88]. It is not the simply embedding AI models into discrete experimental steps for local functional enhancement, such as optimal chemical composition screening, experimental procedure and processing parameter optimization [89–91], and enhanced interpretability of characterization results. The most salient characteristic of SDL lies in its incorporation of an intelligent control center (typically centered around domain-specific LLMs as the core agent), which could autonomously design experimental plans according to the experimental objectives, effectively orchestrate various experimental instruments to perform targeted subtasks, and dynamically adjust and refine the optional workflows and parameters based on the results feedback. Several advanced SDLs even integrate and online academic literature retrieval-extraction module. By extracting and distilling implicit material knowledge, chemical formulations and processing protocols from external scientific literatures, a domain-specific experimental knowledge base is established. It can both extend researchers' exploration ideas and assist AI agents in swiftly generating innovative yet rationally feasible experimental workflows [92–94]. Readers could gain more detailed information of SDLs from these articles [81,83,84,88,95]. Notably, from two key dimensions encompassing experimental workflow automation (hardware) and data-driven decision autonomy (software), Tom *et al*. introduced a hierarchical classification framework for SDLs [81], with levels ranging from L0 (entirely manual) to L5 (fully autonomous multi-round experimentation).

Currently, SDLs are being established all over the world, such as Ada [96], A-Lab [97], AI-chemist [98], and have initially demonstrated substantial potential for accelerating materials research across diverse materials classes, including thin film materials [96], nanoparticles [99], solid-state materials [100], catalysts [101], ferroelectric materials [102], battery materials [103], and also the systematic generation of high-fidelity experimental data. The integration of artificial intelligence decision-making models with mobile robotics and automated laboratory instruments undoubtedly provides SDL with a substantial technological advantage. Operating within a controlled and enclosed environment—although not entirely autonomous due to occasional human interventions such as the replenishment of experimental materials—SDL is capable of executing highly complex and precision-dependent operational sequences continuously and without fatigue. This is achieved through intelligent, algorithm-driven precision control modules in accordance with predefined experimental protocols [104,105]. The secure interrogation and data acquisition of hazardous substances (e.g., toxic compounds and radioactive materials) is even enabled.

The emergence of SDL signifies a paradigm shift in the role position and functional domains of labora-

tory personnel. Rather than serving as direct participants throughout the traditional experimental workflows, researchers take the role of strategic planners and process supervisors of experimental schemes within the novel human-machine collaboration framework [83,106]. Such transformation, through systematically optimized human-machine task reallocation, fosters synergistic optimization of both experimental efficiency and scientific innovation. Besides, it permits researchers to observe material behavior patterns from a third perspective while enabling them to redirect cognitive resources towards resolving complex structure-property relationships in materials science [84]. Furthermore, the integration of heuristic search algorithms with intelligent decision-making models effectively supplants conventional brute-force search methodologies, enabling rapid identification of optimal experimental domains and carries out closed-loop experiments autonomously. Such practice remarkably shortens the materials design and development cycles (~70% or more experimental iterations cycles were wiped out compared to manual experiments), speeding up the discovery of globally performance-optimized materials with significantly reduced required experimental resources. A typical example is the A-Lab [97], an autonomous laboratory for the solid-state synthesis of inorganic powders. In 17 days, this laboratory independently conducted 355 experiments and successfully completed the synthesis and characterization of 41 target compounds out of 58 (74% success rate), which greatly facilitates the practical validation and discovery of innovative materials.

2.2.4 List of current automated and autonomous experimental laboratories

Table 1 list of existing automated and autonomous experimental laboratories

Name	Applicable Materials	Ref
MAOS	Inorganic perovskite quantum dots, nanomaterials	[107]
AMANDA Line One	Organic photovoltaic materials	[103]
BEAR	3D printing of polylactic acid	[108]
CAMEO	Shape memory materials	[109]
Ada	Thin film materials	[81]
RAPID	Metal halide perovskite single crystals	[78]
MFML	Metal nanoparticles	[99]
AI-Chemist	Metal Oxide Photocatalysts, Oxygen Evolution Catalysts	[98]
A robotic platform for the synthesis of colloidal nanocrystals	colloidal nanocrystals	[110]

AR-Chemist	Crystal Growth and Characterization	[111]
Synbot	Organic molecule	[112]
A-Lab	Inorganic compound materials	[97]
RoboChem	Photochemical synthesis	[113]
Smart Dope	Doped Perovskite Quantum Dots	[114]
AlphaFlow	Semiconductor Nanomaterials	[115]
Artificial Chemist	Inorganic perovskite quantum dots	[116]
Coscientist	Chemical reaction	[117]
AMASE	Thin-film materials	[118]

2.2.5 Prospects for future development

The automated and autonomous experimental laboratories represent a transformative advancement for materials research infrastructures, offering significant advantages including elimination of experimental artifacts from potential changes in variable experimental conditions, significant improvements in data quality and throughput, and enhanced reproducibility compared to manual experimentation. However, the widespread deployment of these still confronts various technical barriers [75,84,119].

Firstly, in terms of hardware, the extreme heterogeneity and highly customized nature of experimental equipment in current SDLs lead to significant structural complexity, posing challenges for flexible scalability. The proprietary architectures adopted by instrument manufactures, coupled with the absence of standardized control directives and unified interfacial communication protocols, frequently gives rise to compatibility deficiencies and even conflicts when integrating various vendor-specific experimental devices. Besides, the integration expenditures and maintenance costs could scale exponentially as the number of automated devices increases. Research institutions or teams are forced to invest millions of dollars to retrofit and upgrade existing materials laboratories for SDLs, even greater expenditure for de novo construction, which heavily impeding the global implementation and deployment of SDLs. One promising approach for addressing these fundamental hardware limitations is the systematic decoupling and reconfiguration of experimental components through modular design principles. Lo *et al*. [120] proposed a “frugal twin” strategy whereby the construction of SDLs can be as easy as building with LEGO blocks by developing modular automated hardware with universal interfaces and standardized user interface. This approach allows the experimental components to be

flexibly replaced or added according to practical needs, such as integrating a conductivity meter with probe for conductivity measurement, thereby significantly reducing the reconfiguration time of SDLs targeting new materials system and dramatically compressing the system construction costs to a few thousands of dollars. Additionally, flexibly programmable [121,122] and general-purpose laboratory equipment components, such as multi-channel pipetting robots and mobile robots [101] for flexible operation and parallel experiments, may be also useful building or expanding a versatile, self-contained laboratory.

At the software level, multiple constituting components in a SDL require an effective coordination and management platform to ensure the correct operation of experimental procedures and automated data collection from original devices to a unified database. Paradoxically, existing laboratory experimental job scheduling systems exhibit configurational inflexibility, with most implementations constrained by rigid architectures [123] (e.g., strict computer-instrument pairing). Recognizing this problem, a range of generic software solutions designed for experimental workflow management and hardware integration have been developed, including ChemOS [124,125], NIMS-OS [126], HELAO [127], AresOS [128], ARChemist [111], etc. The standardized communication protocols, such as the SiLA2 standard [129], also facilitates the construction of generic SDL management systems. Furthermore, concerted efforts are devoted to the development of instrument-agnostic experimental data capturing frameworks, such as ESCALATE [130], to enable automated, standardized and complete data documentation of both operational sequences and results information.

Regarding experimental operations, the materials synthesis methods compatible with SDLs remain relatively limited. Existing SDLs are predominantly confined to performing elementary and highly repeatable experiments with clearly predefined operational parameters, such as solution-phase chemical synthesis for targeted materials or application, while struggling to effectively carry out complex sample preparation processes, such as physical deposition (involving magnetron sputtering, molecular beam epitaxy). Furthermore, critical aspects such as cross-modal data integration, real-time parameter adjustment, and nonlinear optimization feedbacks still require varying degrees of human intervention. The materials community needs to systematically optimize and re-engineer the experimental workflows through the development of algorithmically structured operational protocols, decomposing the unstructured experimental procedures into modular procedural components that can be encoded as machine-executable instruction sets. This digital transformation would effectively decouple experimental execution from subjective intervention while concurrently mitigating operational latency inherent in manual process iterations [75,76]. Leroy *et al*. [131–133] developed a general

chemical descriptive language, χDL, which enables the formalization of synthetic operations into standardized and machine-recognizable instructions, and demonstrated its effectiveness by performing several complex organic chemical reactions on their automated experimental platform. In addition, Leong *et al*. [134] proposed a general object-oriented framework for defining well-defined and flexible experimental workflow with self-consistent states and tasks, which could be executed across geographically distributed SDLs collaboratively. There is also a need to develop robust control algorithms to overcome the non-linear characteristics inherent in the material synthesis process.

Finally, the introduction of multi-agent system [135,136]into autonomous materials laboratory should be proactively considered. Most of current autonomous materials laboratories are established based on monolithic architectures, in which a centralized AI agent orchestrates all the task allocation and execution across various instruments. While such systems might be ideal for performing tasks with clearly defined purposes in relatively fixed environments, their scalability constraints become evident as laboratory automation evolves toward more sophisticated and multi-step workflows. Multi-agent systems are the future development direction for SDLs, where diverse agents, with similar or distinct functions, engage together to complete a common goal by cooperating, competing, or being independent of each other. This system transcends the simple aggregation and interconnection of individual agents by placing greater emphasis on the dynamic interaction protocols and collaborative strategies among agents. Additionally, it incorporates mechanisms for self-adjustment and decision-making that are responsive to both the external environment and the conditions of other agents. This may require an iterative process of clearly defining the roles and responsibilities of each intelligent agent in the autonomous materials laboratory to ensure them synergistic collaboration and coherent progression towards predefined experimental objectives. An open agent-to-agent communication protocol [137] , recently introduced by the Google and designed to establish a standardized framework for inter-agent coordination in heterogeneous intelligent agent system, would be helpful for addressing this requirement. Explicitly defined roles for each agent can also be helpful to improve the scalability of the system. When confronted with new requirements or functional adjustments, the system can readily accommodate the changes by introducing new agent roles or adjusting the responsibilities of existing agents, without the need to refactor the entire system.

It is foreseeable that the autonomous materials laboratories will thoroughly revolutionize the experimental infrastructures, transforming material experiment research and development from ‘resource-consuming’

to ’intelligent emergence’. At the same time, the nature of experimental work and the organization structures will also shift towards the mode of multiple intelligent agents’ system. The original human-centered process would be deconstructed and then reconfigured according to the exceptional abilities of the machines. Human researchers would be able to focus on core scientific investigations, while the machines will become an important partner to promote materials innovation. Efficient collaboration between humans and machines will lead to greater influx of reliable experimental data.

2.3 Intelligent system for production management

Undoubtedly, the AI-augmented materials domain-specific computation and experimental infrastructures establish robust enabling conditions for the systematic acquisition of materials data with enhanced efficiency, precision and reproducibility. However, the highly dispersed geographical distribution presents formidable challenges for collaborative advancement, as well as effective transmission of instruction transmission across different platforms, of materials computational and experimental tasks. In this context, establishing a cross-domain collaborative task management system becomes imperative, particularly within the globally integrated smart materials data factory.

2.3.1 Task orchestration, distribution, and result aggregation

Allowing users to flexibly configure and orchestrate different types of tasks based on studies demands, as well as automated task allocation and result aggregation across physical materials laboratories and virtual laboratories constitutes the fundamental function of this system. In conventional paradigms, numerical simulations, experimental synthesis and property characterization tasks that targets specific materials systems are often performed on isolated heterogeneous platforms. The absence of harmonized coordination in parameter systems hinders effective cross-platform data integration and comparative analysis, often necessitating redundant efforts. Differently, this system enables users to architect multi-step sequences with complex logical dependencies in a workflow format. It dynamically allocates task execution instructions to optimal operational nodes by intelligent workflow parsing and scheduling engine.

As the central hub of the globally integrated intelligent material data production infrastructure, an intelligent materials data production control platform holds significant potential for transforming traditional material research working paradigms. Distinct from “producer” role assumed by experimental and computational data

production units, this system enables collaborative governance and task orchestration across both physical materials laboratories and virtual simulation environments through the dynamic optimization of entire research frameworks. Unlike traditional research methods where experiments or numerical simulations are carried out in isolation, such platform could empower researchers to establish cohesive materials discovery pipelines, spanning from theoretical material design, virtually computational screening, practical samples synthesis to performance characterization, while abstracting away laboratory-level operational intricacies and coordination requirements for computational and experimental resources. The deep incorporation of ML models confers the platform with the autonomous decision-making and automated complete data recording throughout the materials data production pipelines, promoting the realization of autonomous batch data production and facilitating nonlinear exponential expansion of materials datasets. The deliberate distribution of localized subtasks to specialized laboratories, as opposed to assigning entire research projects to a single institution, promotes cross-institutional collaborative innovation while ensuring the protection of intellectual property rights for workflow designers. Furthermore, integrated domain-specific large language models (LLMs), utilizing their sophisticated semantic understanding capabilities, facilitate the automatic translation of research objectives expressed by materials scientists in natural language into structured task graphs. These models subsequently compile the task graphs into hierarchically organized workflows according to the logical dependencies and resource constraints among the tasks. And as previously mentioned, the user-designed researcher workflow could be systematically optimized to achieve a globally optimal execution sequences through graph neural network (GNN) models, genetic algorithms and reinforcement learning techniques. The decision-making module within the system subsequently autonomously assigns the tasks to the appropriate execution nodes automatically, according to the matching degree between task attributes and node competencies. For instance, the metallic thin-film synthesis task would be routed to a dedicated facilities possessing certified cleanroom environments and magnetron sputtering instrumentation, thereby ensuring process standardization and experimental reproducibility. With this system, the whole research chain “conceptually design-practically synthesis” for specific materials systems could be carried out smoothly, avoiding research stagnation caused by inadequate laboratory resources or technical constraints, which may trap the forward engineering or reverse design of materials research into local optimization.

Another distinguishing advantage of this system is that, it supports dynamic adjustment of subsequent steps and associated parameters during workflow execution, achieved by harvesting heterogeneous infor-

mation (including operational logs and intermediate results) in real-time from remote distributed computing clusters and materials laboratories. When abnormal operations or parameter deviations beyond preset thresholds are identified in intermediate execution stages, optimization suggestions will be generated via pattern recognition in libraries of successful cases in history. And the system would autonomously perform error correction and process redirection upon receiving authorization from researchers. Furthermore, as the continuous accumulation of execution workflows and associated information (including execution parameters and results), just like human scientists, the intelligent models equipped with active learning algorithms would gradually acquire experimental task prediction capability. The system can prognosticate experimental outcome distributions before experimental trial initiation, and preemptively warn about and autonomously correct potential operation errors, thereby enhancing experimental efficiency and reliability.

By nature, the system, built on a distributed network architecture, effectively integrates various material simulation platforms, and achieves precise digital mirroring of physical laboratory in cyberspace by digital twin technology, thereby providing a fully programmable framework for interdisciplinary research. On top of this system, achieving the standardized registration and preservation of research workflows is of great significance for promoting knowledge accumulation, sharing and reuse within the material domain. Instead of merely modularizing commonly used computational software, experimental instruments, simulation programs and experimental operation sequences, it further necessitates the encapsulation of workflows for solving materials-specific problems into independent components, or decomposition into multiple functionally discrete yet versatile modules across heterogeneous materials innovation pipelines. These functional units, regarded as precious academic assets, should be utilized under the explicit authorization and approval of the authors. Two evident advantages manifest when implementing such practice. On the one hand, it will transform the scientific methodologies, traditionally confined to academic publication and required researchers to reconstruct the procedural code based on textual description and individual understanding, into directly executable algorithm modules. This enables lossless methodological migration and exact reproduction within the scientific community. Subsequent researchers can thereby validate both the effectiveness of the method functionalities and the authenticity and reliability of resultant datasets through rerunning the established workflows. On the other hand, this mechanism constructs an inheritable methodological framework, where researchers could acquire invaluable expertise from predecessors' workflows. Furthermore, these 'Lego' components can be strategically reconfigured through parametric optimization and architectural reconfiguration, allowing scientists to engi-

neer sophisticated frameworks for addressing advanced materials challenges. Both these two aspects will guide the globally integrated smart materials data factories to produce large-scale datasets with enhanced multi-dimensional characteristics. Currently, similar workflow-hosted repositories include WorkflowHub [138] and KNIME Community Hub [139], both of which are mainly for registering and curating computational workflows.

2.3.2 Complete Data Recording and Archiving

Promptly collecting, organizing, and archiving heterogeneous data from distributed task execution nodes constitutes another crucial function of this system. In the data-driven research paradigm, comprehensive and complete data documentation serves as an indispensable cornerstone for upholding the credibility and reliability of scientific findings. This process is of paramount importance as it allows for the rigorous verification and validation of research outcomes. By meticulously recording and describing the data used in a study, researchers enable others to understand the context and nature of the data, thereby facilitating the reproduction and replication of research processes. This transparency not only strengthens the scientific community's confidence in the findings but also ensures that the research can be subjected to thorough scrutiny and evaluation. Consequently, comprehensive data documentation is not merely a procedural formality but a fundamental practice that underpins the integrity of data-driven scientific inquiry.

The pervasive application of digital information technologies within the materials fields has rendered traditional paper-based data recording methods almost obsolete in contemporary research and industrial practices. However, this technological transformation does not imply that valuable data are being recorded completely. Objectively speaking, the scope and content of data collection and documentation remain contingent upon researchers' subjective perceptions and academic interests, which inevitably raises concerns regarding the integrity of archival datasets. Acknowledging this issue, auxiliary tools emerge to facilitate comprehensive data acquisition within laboratories. These tools are grounded in a design concept that employs automation machines or algorithmic scripts, which are inherently devoid of emotional interference and cognitive biases, to replace manual operations. Such substitution effectively reduces human-induced errors, including but not limited to instrument misreading, data omission, and information transcription distortion, thereby guaranteeing the standardization and completeness of the entire data acquisition process.

Nevertheless, these tools remain predominantly confined to verification in controlled environment and practical application within individual laboratory settings. In the globally integrated smart materials data fac-

tory, the system necessitates the implementation of unified data transmission protocols and standardized interfaces to ensure seamless interoperability across heterogeneous data acquisition systems. It should be particularly emphasized that the scope of comprehensive data documentation should encompass not be limited to critical results and key performance parameters. Comprehensive documentation of all information pertaining to research processes, including inputs and outputs, should be systematically maintained. This entails that the system must not only record detailed outcomes and configured parameters at each stage of the workflow but also capture fundamental information regarding task execution nodes (often required from collaborating entities when integrating new data production workshop into globally integrated material data factory) and operational purposes. Even the entire research workflows may necessitate systematic decomposition into provenance and technical metadata for persistent storage.

In turn, this system could promote the implementation of material domain-specific data standardization initiatives. The well-defined data standard specification, developed collaboratively through community discussion, can be transformed into standardized data population templates. When dispatching task directives to various execution nodes by the workflow task-scheduling engine, these templates are simultaneously transmitted to enforce standard-complaint practices being implemented during the initial phase of data production. It should be noted that this does not imply that the system only handles the dissemination and collection of data templates. The raw data files and associated metadata generated by individual execution nodes, are still fully preserved to support future data re-sampling, supplementation, and reuse.

As the increasing rate of materials data production, standardized data archiving practices are becoming critical for efficient discovering, retrieval, and reusability of data assets in future research endeavors. By integrating an intelligent data classification and tagging agent into the system, multi-dimensional annotations, such as typology, source method, and archiving format, can be automatically assigned to heterogeneous datasets upon completion of the research workflow. Furthermore, the integration of material-aware LLMs enables the automatic summarization and pre-population of critical information, including generation objectives and data comments, for the resultant datasets. The workflow parsing engine is essential as well, as it can transform the visually represented research workflow into portable academic asset files. Benefiting from this, researchers could collaborate and share information with partners securely by configuring secure encryption protocols, keeping their academic innovation and procedural methodologies confidential.

Finally, to enhance usability, integrating such system into an electronic laboratory notebook (ELN) is

recommended. This would not only facilitate convenient data observation and analysis for materials scientists, but also allow them, according to research-specific demands, to appropriately supplement and adjust metadata information under controlled access permission, such as adding data annotations and revising experiment configurational parameters. Existing ELNs include Chemotion ELN [140], eLabFTW [141], Kadi4Mat [142].

S3 Constructing the open and automatic pipeline for materials data collection and extraction

3.1 Methods overview for developing materials data extraction modules

3.1.1 Text data

Text is an essential tool for scientists to record precious research findings and express academic viewpoints. And generally, from the perspective of computer science, the text data to be parsed and extracted can be morphologically divided into two types: semi-structured and unstructured text data.

Taking a typical json file as an example, semi-structured scientific data often possess a clear organizational framework, but is without strict scheme definitions, thus allowing data to exist in a loose structure for adapting to a variety of complex and dynamic data scenarios. This type of data has two most obvious characteristics: data is often organized into key-value pairs and has nesting structure. Sometimes it is also self-describing. As a result, the textual data in semi-structured can be extracted by defining character matching or regular expression matching rules [143]. But this method frequently requires materials researchers to be skilled in specialized regular matching knowledge. To mitigate this issue, He *et al*. develop a template-based extraction framework that effectively lowers the technical threshold for researchers to write complex extraction rules, by constructing scalable data extraction templates. While researchers can use such framework or simply ask general-purpose LLMs for help to define extraction rules, it should be noted that the rule-based methods have poor flexibility to adapt various semi-structure textual documents. Moreover, researchers might need to continuously adjust the extraction logic to correctly extraction data.

Unstructured text data is more often seen in open scientific publications. In the history of materials development, abundant valuable research data (e.g., synthetic procedure of experimental sample [144,94,145] and chemical composition and property data [146]) are buried in every corner of journal articles, conference papers, patents and technical reports. To mine this huge "data treasure" for avoiding redundant research resource

consumption, various methods and tools have been developed. The first approach for this task might be the ruled-based systems, including ChemDataExtractor 1.0 [147], which facilities a number of materials property databases [148–151]. These systems are commonly relied on the domain-specific terminology dictionaries and/or regular expression rules (e.g., the chemical formula can be defined by the combination of chemical elements and numbers) that summarized and curated by materials experts [95,152]. It also does not need any data annotations and training and tuning ML models. But as previously stated, these methods are over-reliance on superficial data features and cannot understand the contextual texts, with a limited scope of application scenarios. The extraction process may end with errors due to changes in content description and organization structures.

The integration of Natural Language Processing (NLP) technique and text mining algorithms offer an alternative pathway. Generally speaking, the data extraction process via NLP technologies is divided into three sub-tasks: Named-Entity Recognition (NER) [153,154], Relation Extraction (RE) [155,156] and Co-referencing. The NER task is to extract and categorize entities mentioned in the unstructured text into pre-defined types, and the dedicated tools include ChemDataExtractor [147,157]. The RE task is to capture the binary relationships within sentences and map it to the relevant entities. And Co-referencing is to recognize named entities that refer to the same object (e.g., polymers and their abbreviations) and aggregate them. Given the inherent operational sequential dependencies among these tasks, numerous systems have consolidated them into an integrated task, thus delivering end-to-end application for researchers [158,159]. Currently in materials science, relevant techniques can be mainly divided into two types: pre-training large models and LLMs-based prompt extraction.

Since the fundamental large models have been trained on extensive datasets encompassing rich and generalized linguistic knowledge and semantic information, they can transfer such knowledge to various downstream tasks. This enables them to handle domain-specific tasks effectively and get excellent performance, even with minimal labeled data, thereby significantly reducing the human resources and time cost required for data annotation. And as revealed by systematic comparative analysis, the domain-specific pre-trained models can outperform more complex general models [160,161]. Among large models, transform network-based architectures display superior capability in parsing contextual professional terms within sentences due to their self-attention mechanisms which could effectively capture syntactic dependencies. In materials fields, the BERT model is often selected as the fundamental model due to its outstanding performance in multiple NLP

benchmarks tasks. At present, the pre-trained large models [162] for materials textual data extraction include MatSciBERT [163], MaterialsBERT [164] and BERT-PSIE [165] which concatenates multiple fine-tuned task-targeted BERT models for automating the entire extraction process.

By encoding materials domain knowledge into prompts, researchers can instruct conversational models to understand implicit associations in specialized contexts [166]. This “rules-as-prompts” approach effectively overcomes the transferability limitations of other methods. When processing text data of new material systems, simply adjusting the domain knowledge description in the prompts can dynamically adapt the extraction rules. Moreover, fine-tuning techniques allow LLMs [167] to be optimized for specific domains, thereby achieving more efficient and precise text extraction result than zero-shot learning techniques [168]. This approach has been widely proven feasible by materials scientists and assisted in constructing materials property databases [166,169–171]. It is important to note that due to the existence of hallucination issues, LLMs may fabricate information that is not present in the original input text during the extraction process, for example, LLMs might incorrectly report the numerical data of melting point for SiO_2. In addition, it also has reproducibility issues. Therefore, the extracted results should be scrutinized by human researchers or other specialized ML models.

In any case, it is imperative for materials researchers to carefully encode domain-specific knowledge into machine-understandable language, namely extraction rules. Additionally, when developing or upgrading advanced tools for materials textual data extraction, it should be noted that the varying format, layout and domain-specific textual style of writing in different scientific documents might also inferences with accuracy data extraction.

3.1.2 Image data

Throughout the entire synthesis-characterization-analysis chain in the materials experimental laboratory, image data, mainly generated by atomically resolved characterization instruments (including scanning transmission electron microscopes (STEMs) [172,173], X-ray diffractometers (XRDs) [174,175] and atomic force microscopes (AFMs) [176]), are indispensable nano- and micro-scale information carriers, serving as both the direct ‘window’ to the microstructure and the bridge to the multi-scale materials behavior [177,178]. While often spatially distributed and high-dimensional, they not only can provide fundamentally quantitative micro-structural and functional attributes, such as atomic positions, defects characteristics, element distribution, but also can be used in the derivation of potential attributes, such as band gap, density of states and thermal diffusion coefficient.

Traditional image information extraction relies heavily on manual visual inspection and experience. Inevitably constrained by cognitive limitations and subjective biases, this method is not only inefficient and prone to high error rates but also fails to capture and quantitatively express subtle structural differences, hindering a comprehensive understanding of materials structural properties. Moreover, inconsistency exists when processing the same image by different experts, and the extracted result lacks universal comparability. Under such circumstances, it becomes imperative to develop standardized, highly accurate and robust algorithms that capable of extracting essential materials information from vast image datasets [179].

Initially, the materials image data extraction employs rule-based algorithms, which implemented by manually design image features (e.g., textual, color, shape, length, etc.) for describing the micro-structures [180,181]. While intuitive and require relatively low computational complexity, it is effective only for high-contrast micrographs with homogeneous compositions, while the extraction process depends on domain knowledge and expertise, which may fail to fully exploit the potential information within image data. Likewise, early ML models [182,183] for materials image data extraction are plagued by these factors.

As the advanced stage of AI technology, the deep learning-based models could automatically learn the high-level and abstract representation of image feature through multi-layer neural networks and automatic feature learning techniques [184]. CNN-based models can classify the images at the pixel level and achieve fine-grained portrayal of the materials micro-structures, including grains, phase boundaries and defects. Theoretically, these methods could be used in various tasks of specific materials image data extraction, as long as providing sufficient training dataset. However, most existing DL-based methods for materials image data extraction cannot realize the general and robust recognition for various microscopy data [179]. One reason is that they need large-scale datasets with clear annotations, which is often uncommon in materials community.

In recent years, large-scale pre-trained models [179] have emerged as a research hotspot in the field of AI. By conducting pre-training on vast amounts of unlabeled data, these models could acquire initial feature representations. Then these pre-trained models could be fine-tuned for adapting target materials fields by providing relatively a small amount of labelled data, improving generalizability while reducing the annotation need for massive images. Stepping further, Visual Large Models (VLMs) provide new method for image analysis and data extraction for enhanced applicable scope and reduced labor cost [185].

Despite these technological advancements, fundamental bottlenecks persist in materials image data extraction. The prohibitive cost of data annotation still poses a significant barrier to the broad application of su-

pervised learning ML models, especially for images as complex as atom probe tomography (APT) data. Most of the time, the image data used for training these models is often in several hundred sizes [182], or even as few as several dozen. Such limited dataset size frequently raises concerns within the materials community regarding the models' accuracy and generalizability. Self-supervised learning [186,187] techniques provide alternative pathway, which enabling ML models to learn general features from large volume of unlabeled data designing for certain material-aware tasks (e.g., crystal lattice point prediction and diffraction pattern reconstruction).

3.1.3 Table data

Tables are the crucial carriers of key materials information in documents, particularly in materials literatures. It is reported that ~85% material compositions and their associated properties (e.g., electrical conductivity, thermal conductivity, refractive index) are presented in tables, rather than in texts [188]. However, the variety and complication of tabular formats make extracting data from image-based tables challenging. Unfavorable factors such as complex cell structures (cells may span multiple rows or columns, forming irregular grids), mixed data types (tables can combine text, numbers, and images within a single structure), and hierarchical information (multi-level headings and subheadings establish complex data relationships) precludes standardized parsing tables and data extraction. And more specific issue about extracting materials data has been discussed in ref [189].

Rule-based methods [190,191] were among the earliest approaches used for table detection and data extraction tasks. Initially, these methods rely on Optical Character Recognition (OCR) technologies to analyze the document layout for visual clues that indicate the presence of tables, such as gridlines or aligned text. Then a set of predefined rules and heuristic algorithms are employed to identify tabular structures, determine the boundary of each cell and then capture the data inside these cells. This method could achieve high accuracy[191] when applied to tables with a fixed and known structure. But it has significant flexibility drawbacks: it cannot generalize the extraction of tables that deviate from the expected format (e.g. broken continuity in cross-page tables) or lack clear visual cues, which limits the applicability across different domains.

For such issues, ML techniques have gained prominence for their ability to enhance the flexibility of automatic table data extraction. This approach still relying on OCR, but building ML models, such as support vector machine (SVM), random forests, conditional random fields (CRF), and neural networks [192], on top of words positions. In contrast to rigid rule-based systems, ML models could learn to recognize a wider range of table formats and effectively process common multi-row merged tables. Moreover, with adequate and appro-

priate training data, these ML models can adapt to new domains more easily than rewriting rules. However, ML methods might be limited by the artificial design of feature representations and are highly sensitive to quantity and quality of training data. In materials science, the requirement for expert annotation makes the creation of large-scale training datasets prohibitively expensive, directly limiting the application of these models in long-tail scenarios.

With the rise of computer vision in the last decade, several deep learning architectures have emerged that attempt to solve complex table extraction problem, such as table-transformer [193], DISCOMAT[188], MuTabNet [194] and TableMaster [195]. These models are typically variants of object detection frameworks, identifying and classifying table components such as the table body, columns, rows, and cells. Compared with traditional ML models, this practice requires no data feature engineering.

In recent years, the advent of LLMs unlocks new possibilities for the general and accurate extraction of table data, including TableMaster [196], TableGPT [197], TableLlama [198]. Initially designed for processing NLP tasks, the powerful context modeling and implicit structural reasoning capabilities confer themselves with unique advantages in comprehending non-standard tables. They can comprehend texts surrounding tables, thus permitting more nuanced understanding of table content and structure. Additionally, LLMs can rapidly adapt to certain specialized domain without losing generalized capabilities by easily fine-tuned by further training on domain-specific data or tabular types [199,200], or through prompt engineering. Despite these advantages, LLMs also have limitations. For example, the probabilistic nature of LLMs means that the extraction output is not of reproducibility and reliability. Unlike rule-based systems, LLMs may produce slightly different results even with the same input, hindering the consistency of applications requiring precise and reproducible table data extraction. Furthermore, the local deployment of LLMs for tabular data extraction necessitates intensive resources and substantial computational power. One promising solution may by integrating Retrieval Augmentation Generation (RAG) [201] techniques into LLMs, allowing real-time retrieval of materials professional terminologies from vector databases, and therefore enabling the accurate parsing of specialized symbols and further enhancing the precision of information extraction.

3.1.4 Statistical charts data

Unlike tabular data with relatively regular two-dimensional structures, statistical charts come in diverse representational formats, including line, bar, pie etc. Most charts contain textual elements (title, axis labels, legend labels) and graphical elements (axis tick marks, lines).

Generally, extraction data from charts rely on visual encoding systems to map numerical information to geometric space, necessitating to overcome multiple technical barriers, such as coordinate system transformation, decoupling of graphical elements and semantic alignment between data markers and textual elements. As such, statistical charts data extraction is a complex task of cross-modal information conversion, requiring construction of a multimodal fusion processing framework technically.

OCR plays a foundational role for capturing textual information and symbols in charts, including key semantic units such as axis labels and legend descriptions. It is worthy to note that traditional OCR systems may suffer from accuracy issues when dealing with complex scenarios such as rotated text, blurred characters, and crossing or occlusions of visual symbols, which motivates researchers to develop advanced OCR engines optimized for chart scenarios [202]. After that, computer vision models structure the semantic content of images. ML models, such as CNN[203] or Vision Transformer-base [204,205] large models, can perform fine-grained classification of chart types, identify the spatial distribution of data markers and lines, and establish a mapping relationship between graphical elements and the coordinate system [206]. In this process, deep learning-based instance segmentation techniques can effectively isolate overlapping data sequences, while geometric reasoning algorithms might address the challenge of inverse parsing of nonlinear coordinates (e.g., logarithmic scales). Finally, NLP techniques are used to disambiguate the extracted textual information and unify numerical measurement units.

Notably, recent efforts in statistical chart data extraction show a growing trend towards end-to-end frameworks [205,207–209]. Specifically in material science, Yukari *et al*. develop an open web system, Starrydata2 [210], which could extract digital experimental data about thermoelectric materials from line plots, heat maps and multiple scatter plots in published papers. These technological advancements enable end users to convert statistical charts to spreadsheet-like format (stored in JSON, CSV formats) in transparent manner, reducing demands on domain experts. It is important to mention that current methods still have generalization limitations when processing 3D charts, composite charts (such as those with dual Y-axes), and highly stylized diagrams. This is primarily due to the deep coupling between visual representation and data semantics.

3.2 Future development direction

Looking to the future, two challenges need to be addressed in the development of advanced automated pipelines for materials data collection and extraction. Foremost among these is the issue of cross-modal data

semantic alignment. In literatures of materials science, researchers express their scientific findings and insights in unstructured natural language, complemented by illustrative images, tables, schematic graphs or statistical charts that highlight key data or results. These data are intrinsically interconnected, yet a joint and comprehensive spatiotemporal extraction framework is unrealized by current technologies to link and extract them. The second is the capability to handle dynamically evolving data. Performance degradation data of many materials under service conditions frequently appear as video streams or real-time sensor signals, posing new requirements for process streaming data in pipelines. Successfully overcoming these challenges call for the deep integration of cutting-edge technologies from materials science and computer science. Regardless, the ultimate objective of these technological breakthroughs remains clear: to build a truly intelligent and automated materials data infrastructure, enabling researchers to acquire extensive annotated and structured data from unstructured documents as conveniently as querying online standardized database.

S4 Constructing materials big data federation

4.1 Detailed process for ingesting heterogenous materials data

The process of ingesting heterogeneous materials data into federated material data storage system can be delineated in three steps: extraction, encapsulation, and transmission, which correspond to the following three sequential operational phases:

(1) Generating data descriptive files. Contingent upon the completeness of metadata attached to the uploaded data , the operational process diverges into two distinct pathways (a and b). The completeness assessment protocol for metadata is primarily predicated on the five dimensional metadata framework proposed in S1, and the assessment mechanism specifically targets core descriptors of generic metadata entries, such as resource categories, archiving formats, and permissible external access privileges, while the content that is strongly related to data generation, like experimental parameter configurations, instrument calibration, is deliberately excluded:

a. For uploaded data containing complete and well-documented metadata, LLMs or domain-optimized extraction engines are invoked to automatically extract discrete critical storage descriptive elements (e.g., data structures, types, descriptions) from these metadata, which are subsequently populated into standardized storage descriptive files.

b. For uploaded data with incomplete or missing metadata documentation, further detection on the

available raw data feature extraction tools is needed: when matched parsing tools are successfully detected, storage descriptive files could be automatically constructed through raw data feature extraction and analysis combined with predefined parsing rules and functional descriptions embedded in parsers; otherwise, manual completion mechanism will be triggered, which requires the data contributor to fill in the necessary storage descriptive information.

(2) After the generation of storage descriptive files, the standardized encapsulation subprocess is automatically initiated, integrating the original uploaded datasets with their corresponding storage descriptive files to construct specification-complaint data storage packages. Just as expedited mailing services necessitate the inclusion of essential details like sender and recipient addresses, all these data packages should be labeled with necessary information. These packages should be registered in FAIR-complaint repositories, and assigned with globally unique persistent identifiers (e.g., DOI, Minids [211]) to ensure long-term data findability and citability. Contributor information (e.g., ORCID [212]) will also automatically linked and added via scholarly identity systems. Moreover, tamper-proof digital fingerprints are also generated via cryptographic hashing algorithms (e.g., MD5 [213]), coupled with version-binding mechanisms to guarantee data traceability and integrity verification throughout the data lifecycle.

(3) According to the identified attributes and classification categories, the encapsulated data packages are distributed and securely transferred to remote optimal storage nodes through dedicated data transfer tools, such as Globus [214,215].

4.2 Existing materials databases

Table 2. List of available materials databases

Name	Country	Type	Description	URL links	Access methods	Fees	Ref
Materials Project	America	Comprehensive	A platform delivering web-based access to computed data on bot known and predicted materials, leveraging supercomputing and advanced electronic structure methodologies.	https://next-gen.materialsproject.org/	Web browser, API	Free	[216]
AFLOWLIB	America	Comprehensive	One of the largest databases for computationally investigated materials, containing >3.5 million material entries and >1,100 crystallographic prototypes for structural classification and input generation.	http://aflowlib.org/	Web browser, API	Free	[217,218]
Open Quantum Materials Database (OQMD)	America	Comprehensive	An open-source quantum database for browsing and researching quantum properties and structural information of various materials.	https://oqmd.org/materials/	Web browser, API	Free	[219,220]

NOvel MAterials Discovery (NOMAD)	Germany	Comprehensive	NOMAD is a Free web service for organizing, analyzing, sharing, and publishing materials science data in a structured, FAIR-compliant format, with features for data exploration, download, and analysis.	https://nomad-lab.eu/prod/v1/gui/search/entries	Web-browser, API, Raw data download	Free	[221]

Table 2. (continued) List of available materials databases

Name	Country	Type	Description	URL links	Access methods	Fees	Ref
Materials Cloud	Swiss	Comprehensive	A platform designed for the sharing and dissemination of resources in computational materials science, offering educational, research, and archiving tools; simulation software and services; and curated and raw data.	https://www.materialscloud.org/	API, Raw data download	Free	[222]
Jo int Automated Repository for Various Integrated Simulations (JARVIS)	America	comprehensive	An infrastructure engineered for automating materials discovery and optimization via classical force-field, density functional theory, machine learning, quantum computation, and experimental calculations.	https://jarvis.nist.gov/	Web browser, API, Raw data download	Free	[223,224]

Materials Commons	America	Comprehensive	A data repository and collaboration platform, allowing researchers to collaborate, tracking the history of published data (provenance tracking).	https://materialscommons.org/	Raw data download	Free	[225,226]

Table 2. (continued) List of available materials databases

Name	Country	Type	Description	URL links	Access methods	Fees	Ref
NIST Materials Data Repository	America	Comprehensive	Developed and maintained by the U.S. National Institute of Standards and Technology (NIST), it serves as a comprehensive, multidisciplinary repository of high-quality scientific and technical data. It encompasses experimental measurements, reference standards, computational models, and analytical tools across diverse fields including physics, chemistry, materials science, biotechnology, and computer science.	https://materialsdata.nist.gov/	Web browser, API, Raw data download	Free	-
Material Data Facility (MDF)	America	Comprehensive	A specialized data platform designed for materials science research, offering scalable data publication and discovery services. It enables researchers to publish, preserve, and share materials datasets of all sizes, fostering collaboration and data accessibility within the materials community.	https://www.materialsdatafacility.org/	Web browser, Raw data download	Free	[227,228]

Table 2. (continued) List of available materials databases

Name	Country	Type	Description	URL links	Access methods	Fees	Ref
Materials Genome Engineering Databases (MGED)	China	Comprehensive	A materials data platform that employs schema-free storage for flexible expansion and mining of complex heterogeneous data. Using dynamic data containers, it integrates multi-source data and combines database and application software into an expandable system. It also incorporates high-throughput computing and data-mining tools.	https://www.mgedata.cn/	Web browser, Raw data download	Free	[229]
MatWeb	America	Comprehensive	A specialized materials property database providing standardized technical datasheets for a wide range of engineering materials, including polymeric systems, metallic alloys, ceramics, and advanced functional materials, with comprehensive coverage of mechanical, thermal, and electrical characteristics to support both academic research and industrial applications.	https://www.matweb.com/	Web browser,	Partially for use	-

Inorganic Crystal Structure Database (ICSD)	Germany	Domain-specific	ICSD is a comprehensive repository of over 210,000 inorganic crystal structures, curated by NIST with coverage spanning from 1913 to present.	https://icsd.products.fiz-karlsruhe.de/	Web browser	Paid for use	[67]

Table 2. (continued) List of available materials databases

Name	Country	Type	Description	URL links	Access methods	Fees	Ref
High Through-put Experi-mental Materials (HTEM)	America	Domain-specific	A repository of inorganic thin-film materials data collected during combinatorial experiments at the National Renewable Energy Laboratory	https://htem.nrel.gov/	Web browser, API	Free	[230]
PubChem	America	Domain-specific	The world's largest collection of freely accessible chemical information, providing search services by various identifiers and offering data on chemical and physical properties, biolog-ical activities, safety and toxicity, patents, literature citations and more.	https://pubchem.ncbi.nlm.nih.gov/	Web browser, API, Raw data download	Free	[231–233]
ChemSpider	England	Domain-specific	A specialized database that allows users to search and shar chemistry from 130 million structures from hundreds of sources	http://www.chemspider.com	Web browser	Free	[234]
Polymer Data-base (PoLyInfo)	Japan	Domain-specific	A polymer database providing comprehensive data for poly-meric material design, covering about 100 property types and various polymer types.	https://polymer.nims.go.jp/	Web browser	Free	[235]

Table 2. (continued) List of available materials databases

Name	Country	Type	Description	URL links	Access methods	Fees	Ref
NanoMine	America	Domain-specific	A FAIR-compliant nanocomposite materials database integrating structured schema and ontology for enhanced data interoperability. The platform combines experimental microstructure characterization with simulation tools (e.g., FEA), while providing resource registration to bridge existing datasets with end-user research needs.	https://materialsmine.org/explorer/chart	Web browser	Free	[236–238]
Materials Design Databases	America	Domain-specific	It provides a specialized repository of experimental and computational materials structure data (e.g., XRD, neutron diffraction), enabling streamlined atomistic modeling for materials design and performance analysis. Its curated datasets support direct integration with simulations, leveraging decades of structural and property data for accelerated research	https://www.materialsdesign.com/databases	Raw data download	Free	-
CatApp	America	Domain-specific	A database of calculated reaction and activation energies for elementary coupling reactions on metal surfaces	https://cmr.fysik.dtu.dk/catapp/catapp.html	Raw data download	Free	[239]

Materials Properties Open Database (MPOD)	France	Domain-specific	An online, open-access repository containing quantitative data on the physical properties of crystalline materials.	http://mpod.cimav.edu.mx/	Web browser, API	Free	[240,241]

Table 2. (continued) List of available materials databases

Name	Country	Type	Description	URL links	Access methods	Fees	Ref
Chemistry Database	China	Domain-specific	A specialized chemical database	https://organchem.csdb.cn/scdb/default.asp	Web browser	Free	-
The Materials Provenance Store (MPS)	America	Domain-specific	A high-throughput experimental database for metal-oxide solid-state materials with a detailed full experimental history for each material, including a metadata sequence description of each experimental procedure	https://data.caltech.edu/records/4kk39-69x76	Raw data download	Free	[242]
Tracking materials science data lineage to manage millions of materials experiments and analyses	America	Domain-specific	Contains raw data and metadata from millions of materials synthesis and characterization experiments, as well as the analysis and distillation of that data into property and performance metrics	https://htejcap.org/	Web browser, Raw data download	Free	[243]

4.3 Entry Requirements for Materials Big Data Federations

Materials big-date federation, as outlined in manuscript, provide a robust foundation for storing and circulating massive heterogeneous data. Automated storage-routing mechanism built atop this federation further ensure materials to the best-matched repositories, thus enhancing domain discoverability. To establish this community-level infrastructure, two requirements should be firstly satisfied for member database:

(1) Providing descriptive and precise database introduction in normative metadata format and registering it into a community-wide database metadata catalog.

(2) Opening data upload and access interfaces.

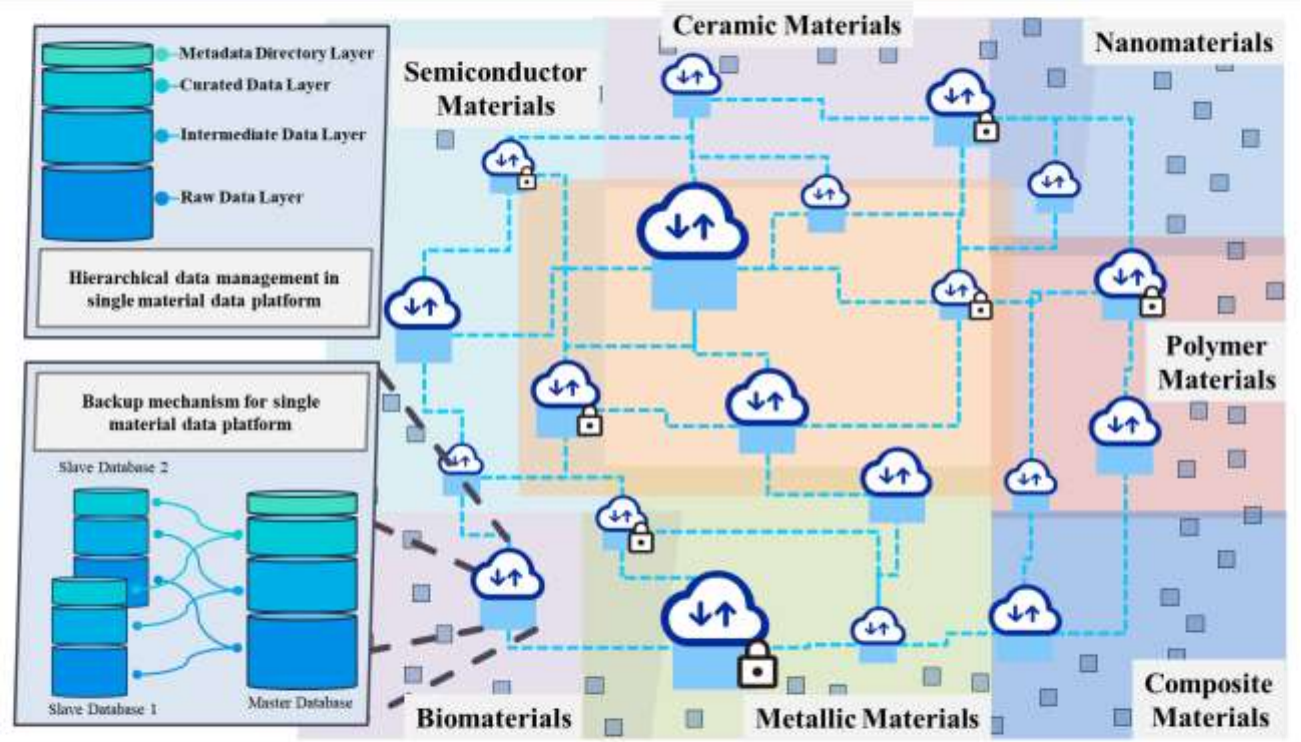

Fig. S1. Architecture of the materials big data federation, which interconnects various sub-domain-specific material data platforms for facilitating the accommodation, management and circulation of escalating heterogeneous materials data. Backup and hierarchical data management mechanisms are mandatory prerequisites for individual platform integration, to ensure long-term data availability and effective governance.

To guarantee the long-term availability, integrity and maintainability of archived data within the materials big data federation, it is recommended that each member storage node implements hierarchical storage management architecture and redundant replica backup system. The former optimizes stored resource configuration through a four-tier strategy (built on data value gradients): platform metadata catalog, curated data, intermediate

data and raw data, facilitating differentiated internal management strategies for different data types and thereby enhancing storage efficiency and cost-effectiveness. The FAIR Data Point [244,245] serves as a reference implementation framework for constructing standardized platform metadata catalog. It enables platforms to expose metadata of their data objects in complained with FAIR principles, supporting data consumers in rapidly discovering, locating and acquiring data resources of interest through metadata interrogation. In parallel, the multi-node backup storage system safeguards against data loss or leakage arising from hardware failures, cyberattacks, or operational errors, strengthening the risk resilience of data assets. Furthermore, trusted data repository certification for member platforms is essential. The primary certification frameworks include CoreTrustSeal [246], Trusted Digital Repositories [247,248], and ISO 1634350 [249]. Among these, CoreTrustSeal is highly recommended due to its rigorous certification criteria, explicit requirements, and comprehensive evaluation protocol for data repositories.

4.4 Blockchain-based Data Security

4.4.1 Secure access

The Attribute-Based Encryption (ABE)-integrated dynamic access control model allows data contributors to flexibly configure multi-dimensional data access policies according to project needs [250–252]. For example, a dataset of alloy compositions can be restricted to institutions with specific qualification certification, and high-temperature testing data remain encrypted until authorized through multi-expert blind view. Such fine-grained data authorization mechanism ensures the controlled sharing of sensitive information.

4.4.2 Data transaction

Smart contract technology in blockchain enables the encoding of data utilization rules into automatically executable digital agreements. For instance, billing mechanism can be established and triggered upon reaching predefined download frequency thresholds, while dynamic pricing models adjust fees based on citation metrics [253], thereby enhancing transaction transparency and execution efficiency. And blockchain ensures data ownership protection by immutably recording data modification histories and ownership transfer trajectories, generating permanent transaction certificates for comprehensive traceability and legal rights preservation [254–256]. The integration of data rating system allows consumers and third-party certification agencies to evaluate datasets based on a range of criteria. This crowd wisdom-driven evaluation mechanism could reflect commu-

nity-defined value assessments and credibility degree for datasets, providing basic value judgment of dataset and facilitating efficient identification of high-quality resources for subsequent data traders. For privacy-sensitive data transaction, the tamper-proof data capsules technique [257] enables controlled sharing under owner authorization with post-usage auto-deletion protocols, which balances data circulations demands with privacy protection and regulatory compliance requirements.

4.4.3 Security protection

The improved Practical Byzantine Fault Tolerance (PBFT) protocol [258] and its derivative algorithms [259] provide the foundational architecture support for building a trustworthy materials data transaction environment. To mitigate potential risks posed by malicious nodes (e.g., profit-driven attacks, transaction tampering, or data forgery) through protocol vulnerabilities (e.g., consensus flaws) or asymmetric technical capabilities (e.g., computing power monopolies), blockchain consensus algorithms (e.g., the CPBFT algorithm, Raft algorithm [260,261], delegate proof of stake [262]) would be beneficial to establish dynamic trust evaluation mechanism. By quantifying the historical performance of individual nodes (e.g., response latency, uploaded data quality scores) into credit ratings, the system enforces tiered penalties against malicious actions, including credit downgrades, transaction restrictions, and network isolation. This strategy significantly strengthens system resilience against network attacks while ensuring long-term data quality stability.

S5 Benefits, barriers and potential solutions for promoting ontology-driven data curation

As presented in the main text, ontology-based data curation is of profound strategic significance for materials science. Firstly, it facilitates the establishment of a semantically interoperable framework across disparate materials data platform. Working as a global data catalog, the materials-specific ontology can enhance the materials scientists' capacity in searching efficiency and scope of interested data, preventing them from being trapped in ever-growing and chaotic data swamp. Based on this, semantic-level association retrieval of arbitrary triples and subgraphs can be realized through query language such as SPARQL. For example, the crystal database of the Materials Project can seamlessly connect with the OQMD database, allowing researchers to execute single queries for candidate materials that "contain three elements, one of which must be oxygen". Secondly, multi-source materials data, after curation, is comparable and readily for use due to the harmonized

format and unified numerical units. More profoundly, the progressive scaling of ontology-structured data will promote the emergence of a globally inter-linked materials data network covering all sub-disciplinary fields. This self-evolving data network can not only clearly define the exploration and cognitive boundaries of materials research, but also directly feed into ML models [263,264], which can augment the feature characterization capability and improve the predictive accuracy and mechanistic interpretability of materials performance prediction results. In turn, this process could be further accelerated with the help of LLMs-based streaming pipelines.

Currently, the ontology has found initial application in materials fields, as prominently demonstrated by domain-specific knowledge graph [265], including MatKG [266], MMKG [267], materials experimental knowledge graph [268], Propnet [269], NanoMine [270]. Nevertheless, significant barriers persist, hindering structured domain-specific knowledge representation.

5.1 Scarcity of domain-specific ontology

While the integration of materials informatics and semantic web technologies has spurred the development of multiple domain-specific ontologies for different materials knowledge systems (as enumerated in **Table 3** and more ontology-related information can be found in MatPortal [271] and review articles [272]), critical domain, such as battery materials and semiconductors, remain devoid of systematic ontology-driven frameworks for structured knowledge representation. This phenomenon can be attributed to various factors, chief among which are the highly intricate and heterogeneous nature of discipline-specific knowledge frameworks and technically, the unfamiliarity of materials specialists with ontological engineering. The persistent absence of disciplinary ontologies thwarts the systematic consolidation of diverse heterogeneous materials datasets into more valuable data assets and even interdisciplinary knowledge networks, thereby compromising data interoperability which is crucial for advancing multidisciplinary materials innovation. Therefore, establishing materials-specific ontologies for structuring and formalizing interconnected materials data network becomes a critical imperative.

Generally, ontologies can be categorized into three hierarchical tiers: top-level ontologies (TLOs), mid-level ontologies (MLOs) and domain-specific ontologies (DLOs), according to their level of conceptual abstraction.

TLOs, serving as facilitators of cross-domain interoperability, define domain-independent core entities, types and universal relationships and thus provide a semantic framework for knowledge integration across

diverse disciplines and application scenarios. The high degree of conceptual abstraction confers these ontologies with broad extensibility and reusability, without being constrained any specific domain. Several widely-adopted TLOs include EMMO [273], BFO [274], SUMO [275], GFO [276], DOLCE [277], Materials Graph Ontology [278].

By extending the generic concepts inherited from TLOs and incorporating widely applicable fundamental concepts within specific domains, MLOs enhance the domain adaptability and application of ontologies. They maintain logical coherence with TLOs and offer standardized interfaces for DLOs development. This dual compatibility allows materials scientists to focus on abstract modeling of core domain knowledge and formal precise expression of conceptual definitions, mitigating underlying technical challenges such as terminological semantic alignment and system interoperability. A notable MLO example in materials science is the PMDco [279].

Essentially, TLOs and MLOs constitute a structured framework for cross-disciplinary knowledge representation. Thus, in ontology engineering practice, these two are commonly developed via a “top-down” strategy. And systematic application of domain-agnostic ontology engineering methodologies and design patterns [280], including Ontology Design Pattern [281], Methontology [282], OTKM [283], Ontology Development 101 [284], DOGMA [285] and UUPON [286], could significantly optimize workflow efficiency and elevated ontology quality. Moreover, to prevent redundant ontology development efforts and enhance the interoperability and connectivity of TLOs and MLOs, the modular integration approach is recommended. By conceptually inheriting and expanding existing standard ontological resources (e.g., QUDT [287], PROV-O [288], CSVW [289]), semantic equivalences can be pre-identified and ontological heterogeneities can be proactively resolved during initial phase, substantially reducing post-hoc alignment costs [290]. Concurrently, it diminishes development complexities and efforts while enhancing ontological reusability and maintainability.

Different from TLOs and MLOs, DLOs primarily focus on modeling granular concepts, attributes, and relationships within specialized knowledge system of research fields. These ontologies can function as specialized extensions of TLOs or fully independent ontological frameworks for specific technical areas, such as additive manufacturing, composite materials. Given the stringent requirement for precise alignment between professional knowledge expression and empirical materials data, its engineering construction predominantly adopts a bottom-up methodological paradigm. It usually maps static data structures from relational databases, including tuples, attributes, integrity constraints, and relationships with other tables, to ontological constructs

such as domain-specific concepts, properties, logical rules, and inter-concept relations [291–294]. Such structured transformation enables the systematic construction of application- or domain-specific ontologies while preserving and extending data semantics and operational constraints inherent in source databases. The methodological implementations could refer to ref [295,296]. Notably, while DLOs developed through this approach effectively address practical demands of specialized communities and establish preliminary knowledge taxonomies, the insufficient conceptual formalization and lack of formal documentation often induce cross-ontology concept alignment challenges and semantic interoperability barriers. Consequently, the construction of DLOs necessitates not only referencing domain-specific controlled vocabularies and relevant upper-level ontologies but also adopting rigorous software engineering methodologies. This is manifested in three key aspects: (1) establishing a comprehensive lifecycle documentation management system; (2) implementing structured version control mechanisms; (3) iterative optimization loops. Such methodological transplantation effectively ensures the traceability, and sustainable evolution of DLOs.

Finally, it is noteworthy that the development of ontologies, whether TLOs, MLOs, or DLOs, are fundamentally contingent upon sustained interdisciplinary collaboration between domain experts and researchers [279], which ensures that ontology modeling aligns with scientific rigor while addressing engineering feasibility. Furthermore, auxiliary tools, such as Protégé [297], OBOWLMorph [298] and Ontopanel [299], coupled with collaborative platform(e.g., Ontology Playground) for ontology development, could facilitate rapid prototyping and engineering implementation by streamlining iterative refinement and consensus-driven standardization.

5.2 Insufficient ontology application for data curation practices

The advancement of ontological application in materials data curation provides robust foundations to mitigate data fragmentation challenges inherent in materials informatics. While several materials ontologies (e.g., Materials Ontology [300], materials design ontology [301,302]) has been introduced to enhance interoperability of heterogeneous data across different databases, the practically broad implementation remains constrained by two fundamental challenges:

(1) the fragmented development landscape of domain-specific ontologies gives rise to paradigmatic misalignment in conceptual frameworks and semantic gaps in terminological definitions across research teams;

(2) the absence of robust maintenance frameworks for ontological systems creates cascading deficiencies such as poorly curated documentation practices and sluggish versioning cycles, severely compromising the

reusability of constructed ontologies.

To navigate these challenges, the materials community must strategically balance horizontal expansion of ontological coverage breadth with advancing deep implementation of existing ontologies in data curation workflows. This necessitates not only enhancing semantic interoperability of existing ontologies but also establishing cross-ontology alignment mechanisms to achieve coherent integration of materials knowledge systems. Only through embedding domain ontologies throughout materials data curation processes and implementing closed-loop feedback mechanisms between ontology refinement and data governance, can we establish interoperable semantic pathways for multi-source heterogeneous data, ultimately establishing a robust foundational knowledge infrastructure for materials innovation.

5.3 Deficient ontological axiom formulation

The principled axiomatization of materials-specific ontologies not only provides semantics for unifying multi-fidelity simulation datasets, but also establishes machine-reasoning-ready schema, enabling logic-driven cross-domain knowledge integration in materials informatics. While existing material-specific ontologies realize the structured representation of conceptual hierarchies and relationships, they remain limited in adequately formalizing domain-specific logical constraints. The paucity of domain-specific axiomatic frameworks obstructs the elucidation of underlying materials principles and impedes data derivation through systematic logical inference. A key factor contributing to this is the limited capacity of formalized logical rules. The construction of domain axioms often requires the transformation of disciplinary regularities into formalized logical systems. But the extremely complexity of materials knowledge systems often exceeds the expressive capacity of first-order logic to completely capture the multi-level correlation between structure and performance. For example, within the “functional primitive” theoretical framework, the material properties are governed by the synergistic interplay of microscopic primitives including crystalline configuration, defect architectures, electronic states. The formalization of the constitutive principles governing primitive sequencing and nonlinear macroscopic property amplification necessitates an axiomatic framework that rigorously incorporates inter-primitive coupling dynamics, critical size effects, and quantum confinement phenomena. Such axiomatic framework encompasses not only the static structural descriptors, but also the temporal evolution principles (e.g., modulated structure formation mechanisms), which thereby imposing stringent requirements on the representational expressiveness of ontological language.

“Hybrid modelling” strategy may be a viable approach to overcome this bottleneck: on the one hand, constructing atomic axioms with description logic, e.g., defining the mutual exclusivity constraints of crystal lattice configurations and defect structures; on the other hand, implementing data-driven ML models to infer latent governing principles for computationally intractable relationships (e.g., equivalence of multi-scale processing history, property, composition). And then feeding these implicit association rules back into ontological axiom systems for further refinement. Furthermore, developing community- or domain-level modular axiom libraries is also recommended. By encoding the intrinsic governing mechanisms of micro-structural interactions as self-contained clusters that enable dynamic combination of cross-category rules via logical operators, it not only enhances the scalability of domain-specific axiomatic systems while establishing formal reasoning foundations for inverse materials design.

S6 Current practices in promoting data standardization in materials science

Table 3. List of existing practices for promoting data standardization in materials science

Full name	Abbreviation	Type	Brief description	Ref
Materials And Molecules Basic Ontology	MAMBO	ontology	A domain-specific ontology for molecular materials, focusing on enhancing structured data retrieval, complex modeling workflow development and integrating data from computational simulations and empirical experiments	[303]
Additive Manufacturing Ontology	AMONTOLOGY	Ontology	A domain-specific ontology extends AMProcessOntology and ModelOntology to formally describe entities that capture knowledge about characteristics of computational models for AM processes	-
Materials Design Ontology	MDO	Ontology	A domain-specific ontology for materials design fields, representing the domain knowledge specifically related to solid-state physics and computational models for AM processes	[301]
Platform MaterialDigital Core Ontology	PMDCO	Ontology	A mid-level ontology bridging materials science application ontologies with domain-neutral top-level ontologies	[279]
Materials Graph Ontology	-	Ontology	A top-level materials ontology for inter-relating heterogeneous data	[278]
Laser Powder Bed Fusion Ontology	LPBFO	Ontology	A domain-specific ontology, build on BFO 3.0 and BWMD_mid, formalizes Laser Powder Bed Fusion (LPBF)/Selective Laser Melting (SLM) process.	-
Materials Data Vocabulary	NMPRVOCAB	Controlled Vocabulary	A version of the Materials Data Vocabulary structured as Simple Knowledge Organization System (SKOS), describing the applicability to materials science of records in the NIST Materials Resource Registry	[304]

Table 3. (continued) List of existing practices for promoting data standardization in materials science

Full name	Abbreviation	Type	Brief description	Ref
Virtual Materials Marketplace Ontologies	VIMMP ONTOLOGIES	Ontology	A market-level ontologies to characterize services, models, and interactions between users. EMMO is used as a top-level ontology	[305]
MaterialsMine	MM	Ontology	A materials ontology to support data publication involving nanomaterials and metamaterials	-
Dental Restorative Materials Ontology	DRMO	Ontology	A domain-specific ontology for dental restoration materials, integrating concepts from clinical dentistry and materials science. It reuses and extends ontologies from the OBO Foundry (e.g., OHD), DEB, and FGM while adapting terminology for clinical consistency.	[306]
Matolab Tensile Test Ontology	MOL_TENSILE	Ontology	An ontology for describing the tensile test process, made in the Materials Open Lab Project	-
Devices, Experimental scaffolds and Biomaterials Ontology	DEB	Ontology	An ontology developed to facilitate information curation in the area of medical devices, experimental scaffolds and biomaterials.	[307]
MatoLab Brinell Test Ontology	MOL_BRINELL	Ontology	An ontology for describing the Brinell hardness testing process, made in the Materials Open Lab Project	-
Building Material Ontology	BUILDMAT	Ontology	A domain-specific ontology that defines the main concepts of building material, types, layers, and properties	-
MatOnto Ontology	MATONTO	Ontology	A domain-specific ontology for materials science	[308]

Table 3. (continued) List of existing practices for promoting data standardization in materials science

Full name	Abbreviation	Type	Brief description	Ref
Material Science and Engineering Ontology	MSEO	Ontology	Built on the Common Core Ontology stack, it allows materials scientists and engineers to represent their experiments and resulting data	-
Mat-O-Lab container ontology	MOCO	Ontology	A lightweight ontology to describe the structure of tabular (series) data stored in hdf5 container	-
The MatWerk ontology	MWO	Ontology	A ontology designed to represent the scientific and infrastructural status of the Materials science and Engineering community	-
Standards-Specific Ontology Standard	SSOS	Ontology	A top-level generic ontology describing the lifecycle of consensus-based standardization projects and published standardization deliverables	-
Mechanical testing ontology	MTO	Ontology	A domain-specific ontology formalizes standardized mechanical testing methods based on ISO 23718 terminology	-
Thin-film solar cell ontology	TFSCO	Ontology	A domain-specific ontology that provides a model of the manufacturing and characterization of perovskite solar cells	-
Materials Mechanics Ontology	MECH	Ontology	Based on PMDCO, it formalizes fatigue-related concepts while incorporating fundamental MSE elements like crystallographic defects and microstructural entities to enhance mechanical property characterization within materials science framework	-
European Materials Modelling Ontology	EMMO	Ontology	A top-level ontology for modeling materials knowledge, covering physical science, analytical philosophy, etc., to and bridging physical reality, materials characterization, and computational modelling.	[273]
NanoParticle Ontology	NPO	Ontology	A domain-specific ontology for cancer nanotechnology research	[309]

Materials Ontology	-	Ontology	An ontology designed to facilitate the exchange of materials information and knowledge among different databases	[300]

Table 3. (continued) List of existing practices for promoting data standardization in materials science

Full name	Abbreviation	Type	Brief description	Ref
eNanoMapper ontology	-	Ontology	A domain - specific ontology for assessing nanomaterial-related risks from an engineering perspective	[310]
Plinius ontology	-	Ontology	A domain-specific ontology for ceramics	[311]
Semantic LAminated Composites Knowledge management System ontology	SLACKS ontology	Ontology	A domain-specific ontology for Laminated composites	[312]
Functionally Graded Materials Ontology	FGM Ontology	Ontology	A domain-specific ontology designed for the integration and management of heterogeneous data related to composite materials, with a particular emphasis on functionally graded materials.	[313,314]
Metallic Materials Ontology	MMOY	Ontology	A domain-specific ontology that built on Yago and designed for metals	[315]
FreeClassOWL ontology	-	Ontology	A domain-specific ontology designed to formalize the data of construction and building materials	[316]
Materials data identifier for materials genome engineering	-	Standardized specification	A persistent unique identifier scheme for digital resource registration and management in data-driven paradigms	[317]

Terminology for materials genome engineering	-	Standardized specification	A specification for promoting the construction of fundamental material terminology system	[318]

Table 3. (continued) List of existing practices for promoting data standardization in materials science

Full name	Abbreviation	Type	Brief description	Ref
General requirements of materials calculations for materials genome engineering	-	Standardized specification	A specification defining requirements for computational materials data storage and multi-node aggregation. It provides normative support for data fields integrity, information reusability, raw data traceability, and inter-data correlations	[319]
General requirements of experiments for materials genome engineering	-	Standardized specification	A specification defining requirements for experimental materials data storage and multi-node aggregation. It provides normative support for data fields integrity, information reusability, raw data traceability, and inter-data correlations	[320]
Persistent IDentification of INStruments	PIDINST	Standardized specification	A general metadata schema for global persistent unique identification and measurement instrument resolution	[321]
Metadata scheme for the description of microstructures	-	Standardized specification	A metadata for geometric description and data exchange of static 3D microstructure	[322]
ISA-TAB-Nano	-	Standardized specification	This standard specifies the format for normalized representation and sharing of nanomaterials, small molecules and biological specimens, along with their essay characterization data	[323]

Table 3. (continued) List of existing practices for promoting data standardization in materials science

Full name	Abbreviation	Type	Brief description	Ref
General requirements of materials calculations for materials genome engineering	-	Standardized specification	A specification defining requirements for computational materials data storage and multi-node aggregation. It provides normative support for data fields integrity, information reusability, raw data traceability, and inter-data correlations	[319]
General requirements of experiments for materials genome engineering	-	Standardized specification	A specification defining requirements for experimental materials data storage and multi-node aggregation. It provides normative support for data fields integrity, information reusability, raw data traceability, and inter-data correlations	[320]
Persistent IDentification of INStruments	PIDINST	Standardized specification	A general metadata schema for global persistent unique identification and measurement instrument resolution	[321]
Metadata scheme of lattice thermal conductivity from first-principles calculations	-	Standardized specification	A kind of metadata scheme for complete documentation of lattice thermal conductivity obtained from first-principles calculations	[324]

Electronic Structure Common Data Format	ESCDF	Standardized specification	It mainly used to standardize electronic structure data format	[325]

References

[1] Weibel S, Kunze J, Lagoze C *et al. Dublin Core Metadata for Resource Discovery*. RFC Editor, 1998: RFC2413.

[2] Herschel M, Diestelkämper R, Ben Lahmar H. A survey on provenance: What for? What form? What from? *The VLDB Journal* 2017; **26**: 881–906.

[3] Deelman E, Berriman B, Chervenak A, Corcho O, Groth P and Moreau L. Metadata and provenance management. *Scientific Data Management*, 2009, 465–98.

[4] H S, S L, M S *et al.* AVOCADO: visualization of workflow-derived data provenance for reproducible biomedical research. *Computer graphics forum* 2016; **35**: 481–490.

[5] Hoekstra R, Groth P. PROV-O-Viz - Understanding the Role of Activities in Provenance. In: Ludäscher B, Plale B (eds.). *Provenance and Annotation of Data and Processes*. Vol 8628. Cham: Springer International Publishing, 2015, 215–20.

[6] Khan FZ, Soiland-Reyes S, Sinnott RO *et al.* Sharing interoperable workflow provenance: A review of best practices and their practical application in CWLProv. *Gigascience* 2019; **8**: giz095.

[7] Moreau L, Freire J, Futrelle J *et al.* The open provenance model: an overview. In: Freire J, Koop D, Moreau L (eds.). *Provenance and Annotation of Data and Processes*. Vol 5272. Berlin, Heidelberg: Springer Berlin Heidelberg, 2008, 323–6.

[8] Missier P, Belhajjame K, Cheney J. The W3C PROV family of specifications for modelling provenance metadata. In: *16th International Conference on Extending Database Technology (EDBT 2013)*, Genoa, Italy. Association for Computing Machinery: New York, NY, 2013, 773–6.

[9] Leipzig J, Nüst D, Hoyt CT *et al.* The role of metadata in reproducible computational research. *Patterns* 2021; **2**: 100322.

[10] The Library of Congress. *PREMIS: Preservation Metadata Maintenance Activity*. https://www.loc.gov/standards/premis (24 April 2025, date last accessed).

[11] Abelson H, Adida B, Linksvayer M *et al.* CC REL: The Creative Commons Rights Expression Language. In: Dulong De Rosnay M, De Martin JC (eds.). *Digital Humanities Series*. Vol 2. 1st ed. Cambridge, UK: Open Book Publishers, 2012.

[12] Hohenberg P, Kohn W. Inhomogeneous Electron Gas. *Phys Rev* 1964; **136**: B864–71.

[13] Kohn W, Sham LJ. Self-Consistent Equations Including Exchange and Correlation Effects. *Phy Rev* 1965; **140**: A1133–8.

[14] Jones RO. Density functional theory: its origins, rise to prominence, and future. *Rev Mod Phys* 2015; **87**: 897–923.

[15] Van Gunsteren WF, Berendsen HJC. Computer simulation of molecular dynamics: methodology, applications, and perspectives in chemistry. *Angew Chem Int Ed Engl* 1990; **29**: 992–1023.

[16] Rapaport DC. *The Art of Molecular Dynamics Simulation*. Cambridge, UK: Cambridge university press, 2004, 11–43.

[17] Metropolis N, Ulam S. The monte carlo method. *J Am stat assoc* 1949; **44**: 335–41.

[18] Rubinstein RY, Kroese DP. *Simulation and the Monte Carlo Method*. Hoboken, NJ: John Wiley & Sons, 2016, 1–47.

[19] Steinbach I. Phase-field models in materials science. *Modell Simul Mater Sci Eng* 2009; **17**: 073001.

[20] Provatas N, Elder K. *Phase-Field Methods in Materials Science and Engineering*. Hoboken, NJ: John Wiley & Sons, 2011, 9–25.
[21] Fumanal M, Capano G, Barthel S *et al.* Energy-based descriptors for photo-catalytically active metal–organic framework discovery. *J Mater Chem A* 2020; **8**: 4473–82.
[22] Zhao W, Yan P, Li B *et al.* Accelerated synthesis and discovery of covalent organic framework photocatalysts for hydrogen peroxide production. *J Am Chem Soc* 2022; **144**: 9902–9.
[23] Back S, Tran K, Ulissi ZW. Discovery of Acid-Stable Oxygen Evolution Catalysts: High-throughput computational screening of equimolar bimetallic oxides. *ACS Appl Mater Interfaces* 2020; **12**: 38256–65.
[24] Wu L, Guo T, Li T. Data‐Driven High‐throughput rational design of double‐atom catalysts for oxygen evolution and reduction. *Adv Funct Mater* 2022; **32**: 2203439.
[25] Nath P, Plata JJ, Santana-Andreo J *et al.* High-throughput screening of the thermoelastic properties of ultrahigh-temperature ceramics. *ACS Appl Mater Interfaces* 2021; **13**: 29843–57.
[26] Liu B, Zhao J, Liu Y *et al.* Application of high-throughput first-principles calculations in ceramic innovation. *J Mate Sci Technol* 2021; **88**: 143–57.
[27] Kulik HJ, Tiwary P. Artificial intelligence in computational materials science. *MRS Bull* 2022; **47**: 927–9.
[28] Huang JS, Liew JX, Ademiloye AS *et al.* Artificial intelligence in materials modeling and design. *Arch Computat Methods Eng* 2021; **28**: 3399–413.
[29] Schleder GR, Padilha ACM, Acosta CM *et al.* From DFT to machine learning: recent approaches to materials science–a review. *J Phys Mater* 2019; **2**: 032001.
[30] Huang B, Von Rudorff GF, Von Lilienfeld OA. The central role of density functional theory in the AI age. *Science* 2023; **381**: 170–5.
[31] Zhang L, Han J, Wang H *et al.* Deep potential molecular dynamics: a scalable model with the accuracy of quantum mechanics. *Phys Rev Lett* 2018; **120**: 143001.
[32] Jia W, Wang H, Chen M *et al.* Pushing the Limit of Molecular Dynamics with Ab Initio Accuracy to 100 Million Atoms with Machine Learning. In: *International Conference for High Performance Computing, Networking, Storage and Analysis (SC 2020*), Atlanta, GA, USA. IEEE Computer Society: Los Alamitos, CA, 2020, 1–14.
[33] Das S, Kanungo B, Subramanian V *et al.* Large-Scale Materials Modeling at Quantum Accuracy: Ab Initio Simulations of Quasicrystals and Interacting Extended Defects in Metallic Alloys. In: I*nternational Conference for High Performance Computing, Networking, Storage and Analysis (SC 2023)*, Denver, CO, USA. Association for Computing Machinery: New York, NY, 2023, 1–12.
[34] Brockherde F, Vogt L, Li L *et al.* Bypassing the Kohn-Sham equations with machine learning. *Nat Commun* 2017; **8**: 872.
[35] Von Lilienfeld OA, Müller K-R, Tkatchenko A. Exploring chemical compound space with quantum-based machine learning. *Nat Rev Chem* 2020; **4**: 347–58.
[36] Bogojeski M, Brockherde F, Vogt-Maranto L *et al.* Efficient prediction of 3D electron densities using machine learning. arXiv:1811.06255.
[37] Tawfik SA, Gupta S, Venkatesh S. Predicting the electron density of charged systems using machine learning. *J Phys Chem A* 2025 ;**129**: 2117–22.
[38] Li Z, Cao B, Jiao R *et al.* Materials generation in the era of artificial intelligence: a comprehensive survey. arXiv: 2505.16379.

[39] Ruthotto L, Haber E. An introduction to deep generative modeling. *GAMM-Mitteilungen* 2021; **44**: e202100008.
[40] Zeni C, Pinsler R, Zügner D *et al.* A generative model for inorganic materials design. *Nature* 2025; **639**: 624–32.
[41] Chen Y, Wang X, Deng X *et al.* MatterGPT: a generative transformer for multi-property inverse design of solid-state materials. arXiv: 2408.07608.
[42] Su T, Cao B, Hu S *et al.* CGWGAN: crystal generative framework based on Wyckoff generative adversarial network. *J Mater Inf* 2024; **4**: 20.
[43] Ghiringhelli LM, Vybiral J, Levchenko SV *et al.* Big data of materials science: critical role of the descriptor. *Phys Rev Lett* 2015; **114**: 105503.
[44] Liu Y, Zhao T, Ju W *et al.* Materials discovery and design using machine learning. *J Materiomics* 2017; **3**: 159–77.
[45] Wang Y, Wagner N, Rondinelli JM. Symbolic regression in materials science. *MRS Commun* 2019; **9**: 793–805.
[46] Makke N, Chawla S. Interpretable scientific discovery with symbolic regression: a review. *Artif Intell Rev* 2024; **57**: 2.
[47] Guo Z, Hu S, Han Z-K *et al.* Improving Symbolic regression for predicting materials properties with iterative variable selection. *J Chem Theory Comput* 2022; **18**: 4945–51.
[48] Oviedo F, Ferres JL, Buonassisi T *et al.* Interpretable and explainable machine learning for materials science and chemistry. *Acc Mater Res* 2022; **3**: 597–607.
[49] Merchant A, Batzner S, Schoenholz SS *et al.* Scaling deep learning for materials discovery. *Nature* 2023;**624**:80–5.
[50] Mathew K, Montoya JH, Faghaninia A *et al.* Atomate: A high-level interface to generate, execute, and analyze computational materials science workflows. *Comput Mater Sci* 2017; **139**: 140–52.
[51] Jain A, Ong SP, Chen W *et al.* FireWorks: a dynamic workflow system designed for high‐throughput applications. *Concurr Comp-Pract E* 2015; **27**: 5037–59.
[52] Pizzi G, Cepellotti A, Sabatini R *et al.* AiiDA: automated interactive infrastructure and database for computational science. *Comput Mater Sci* 2016; **111**: 218–30.
[53] Huber SP, Zoupanos S, Uhrin M *et al.* AiiDA 1.0, a scalable computational infrastructure for automated reproducible workflows and data provenance. *Sci Data* 2020; **7**: 300.
[54] Yang X, Wang Z, Zhao X *et al.* MatCloud, a high-throughput computational materials infrastructure: present, future visions, and challenges. *Chinese Phys B* 2018; **27**: 110301.
[55] Bhatia H, Di Natale F, Moon JY *et al.* Generalizable coordination of large multiscale workflows: challenges and learnings at scale. In: *International Conference for High Performance Computing, Networking, Storage and Analysis (SC 2021)*, St. Louis, MO, USA. Association for Computing Machinery: New York, NY, 2021, 1–16.
[56] Tong Z, Chen H, Deng X *et al.* A scheduling scheme in the cloud computing environment using deep Q-learning. *Inform Sciences* 2020; **512**: 1170–91.
[57] Ong SP, Richards WD, Jain A *et al.* Python materials genomics (pymatgen): a robust, open-source python library for materials analysis. *Comput Mater Sci* 2013; **68**: 314–9.
[58] Ward L, Dunn A, Faghaninia A *et al.* Matminer: an open source toolkit for materials data mining. *Comput Mater Sci* 2018; **152**: 60–9.

[59] Amstutz P, Crusoe MR, Tijanić N *et al.* Common workflow language, v1. 0. https://research.manchester.ac.uk/en/publications/common-workflow-language-v10/ (30 April 2025, date last accessed).
[60] Goble C, Cohen-Boulakia S, Soiland-Reyes S *et al.* FAIR computational workflows. *DI* 2020; **2**: 108–21.
[61] Huber SP, Bosoni E, Bercx M *et al.* Common workflows for computing material properties using different quantum engines. *npj Comput Mater* 2021; **7**: 136.
[62] Coleman T, Casanova H, Pottier L *et al.* WfCommons: a framework for enabling scientific workflow research and development. *Future Gener Comp Sy* 2022; **128**: 16–27.
[63] Versluis L, Mathá R, Talluri S *et al.* The workflow trace archive: open-access data from public and private computing infrastructures. *IEEE Trans Parallel Distrib Syst* 2020; **31**: 2170–84.
[64] Li J, Lim K, Yang H *et al.* AI Applications through the whole life cycle of material discovery. *Matter* 2020; **3**: 393–432.
[65] Belsky A, Hellenbrandt M, Karen VL *et al.* New developments in the inorganic crystal structure database (ICSD): accessibility in support of materials research and design. *Acta Crystallogr B Struct Sci* 2002 ;**58**: 364–9.
[66] Hellenbrandt M. The inorganic crystal structure database (ICSD)—present and future. *Crystallogr Rev* 2004; **10**: 17–22.
[67] Zagorac D, Müller H, Ruehl S *et al.* Recent developments in the inorganic crystal structure database: theoretical crystal structure data and related features. *J Appl Crystallogr* 2019; **52**: 918–25.
[68] Sun W, Dacek ST, Ong SP *et al.* The thermodynamic scale of inorganic crystalline metastability. *Sci Adv* 2016; **2**: e1600225.
[69] Grover V, Mandal BP, Tyagi AK. Solid state synthesis of materials. In: Tyagi AK, Ningthoujam RS (eds.). *Handbook on Synthesis Strategies for Advanced Materials : Volume-I: Techniques and Fundamentals*. Singapore: Springer, 2021, 1–49.
[70] Elias JR, Chard R, Libera JA *et al.* The Manufacturing Data and Machine Learning Platform: Enabling Real-time Monitoring and Control of Scientific Experiments via IoT. In: *6th IEEE World Forum on Internet of Things (WF-IoT 2020),* New Orleans, LA, USA. IEEE Computer Society: Los Alamitos, CA, 2020, 1–2.
[71] Chan EM, Xu C, Mao AW *et al.* Reproducible, High-throughput synthesis of colloidal nanocrystals for optimization in multidimensional parameter space. *Nano Lett* 2010; **10**: 1874–85.
[72] Sanderson K. Automation: Chemistry shoots for the moon. *Nature* 2019; **568**: 577–9.
[73] Whitacre JF, Mitchell J, Dave A *et al.* An autonomous electrochemical test stand for machine learning informed electrolyte optimization. *J Electrochem Soc* 2019; **166**: A4181–7.
[74] Manzano JS, Hou W, Zalesskiy SS *et al.* An autonomous portable platform for universal chemical synthesis. *Nat Chem* 2022; **14**: 1311–8.
[75] Häse F, Roch LM, Aspuru-Guzik A. Next-generation experimentation with self-driving laboratories. *Trends Chem* 2019; **1**: 282–91.
[76] Abolhasani M, Brown KA, Guest Editors. Role of AI in experimental materials science. *MRS Bull* 2023;**4 8**: 134–41.
[77] Wagner J, Berger CG, Du X *et al.* The evolution of materials acceleration platforms: toward the laboratory of the future with AMANDA. *J Mater Sci* 2021; **56**: 16422–46.
[78] Li Z, Najeeb MA, Alves L *et al.* Robot-accelerated perovskite investigation and discovery. *Chem Mater* 2020; **32**: 5650–63.

[79] Bergstra J, Bengio Y. Random search for hyper-parameter optimization. *J Mach Learn Res* 2012; **13**: 281–305.
[80] Bergstra J, Bardenet R, Bengio Y *et al.* Algorithms for hyper-parameter optimization. *Proceedings of the 25th International Conference on Neural Information Processing Systems*. Red Hook, NY, USA: Curran Associates Inc., 2011, 2546–54.
[81] Tom G, Schmid SP, Baird SG *et al.* Self-driving laboratories for chemistry and materials science. *Chem Rev* 2024; **124**: 9633–732.
[82] Aspuru-Guzik A, Persson K. Materials acceleration platform: accelerating advanced energy materials discovery by integrating high-throughput methods and artificial intelligence. *Mission Innovation* 2018.
[83] Peng X, Wang X. Next-generation intelligent laboratories for materials design and manufacturing. *MRS Bull* 2023; **48**: 179–85.
[84] Stach E, DeCost B, Kusne AG *et al.* Autonomous experimentation systems for materials development: a community perspective. *Matter* 2021; **4**: 2702–26.
[85] Abolhasani M, Kumacheva E. The rise of self-driving labs in chemical and materials sciences. *Nat Synth* 2023; **2**: 483–92.
[86] Stein HS, Gregoire JM. Progress and prospects for accelerating materials science with automated and autonomous workflows. *Chem Sci* 2019; **10**: 9640–9.
[87] Coley CW, Eyke NS, Jensen KF. Autonomous discovery in the chemical sciences part i: progress. *Angew Chem Int Ed* 2020; **59**: 22858–93.
[88] Flores-Leonar MM, Mej á-Mendoza LM, Aguilar-Granda A *et al.* Materials acceleration platforms: on the way to autonomous experimentation. *Curr Opin Green Sustainable Chem* 2020; **25**: 100370.
[89] Perera D, Tucker JW, Brahmbhatt S *et al.* A platform for automated nanomole-scale reaction screening and micromole-scale synthesis in flow. *Science* 2018; **359**: 429–34.
[90] Vaucher AC, Schwaller P, Geluykens J *et al.* Inferring experimental procedures from text-based representations of chemical reactions. *Nat Commun* 2021; **12**: 2573.
[91] Kim E, Huang K, Jegelka S *et al.* Virtual screening of inorganic materials synthesis parameters with deep learning. *npj Comput Mater* 2017; **3**: 53.
[92] Coley CW, Thomas DA, Lummiss JAM *et al.* A robotic platform for flow synthesis of organic compounds informed by AI planning. *Science* 2019; **365**: eaax1566.
[93] Mehr SHM, Craven M, Leonov AI *et al.* A universal system for digitization and automatic execution of the chemical synthesis literature. *Science* 2020; **370**: 101–8.
[94] Yang X, Zhuo Y, Zuo J *et al.* PcMSP: A Dataset for Scientific Action Graphs Extraction from Polycrystalline Materials Synthesis Procedure Text. arXiv:2210.12401.
[95] Wang S, Wan Y, Song N *et al.* Automatically Generated Datasets: Present and Potential Self-Cleaning Coating Materials. *Sci Data* 2024; **11**: 146.
[96] MacLeod BP, Parlane FGL, Morrissey TD *et al.* Self-driving laboratory for accelerated discovery of thin-film materials. *Sci Adv* 2020; **6**: eaaz8867.
[97] Szymanski NJ, Rendy B, Fei Y *et al.* An autonomous laboratory for the accelerated synthesis of novel materials. *Nature* 2023; **624**: 86–91.
[98] Zhu Q, Zhang F, Huang Y *et al.* An all-round AI-Chemist with a scientific mind. *Nat Sci Rev* 2022; **9**: nwac190.
[99] Tao H, Wu T, Kheiri S *et al.* Self - driving platform for metal nanoparticle synthesis: combining microfluidics and machine learning. *Adv Funct Mater* 2021; **31**: 2106725.

[100] Li J, Li J, Liu R *et al.* Autonomous discovery of optically active chiral inorganic perovskite nanocrystals through an intelligent cloud lab. *Nat Commun* 2020; **11**: 2046.

[101] Burger B, Maffettone PM, Gusev VV *et al.* A mobile robotic chemist. *Nature* 2020; **583**: 237–41.

[102] Liu Y, Kelley KP, Vasudevan RK *et al.* Experimental discovery of structure–property relationships in ferroelectric materials via active learning. *Nat Mach Intell* 2022; **4**: 341–50.

[103] Du X, Lüer L, Heumueller T *et al.* Elucidating the full potential of OPV materials utilizing a high-throughput robot-based platform and machine learning. *Joule* 2021; **5**: 495–506.

[104] Ahmadi M, Ziatdinov M, Zhou Y *et al.* Machine learning for high-throughput experimental exploration of metal halide perovskites. *Joule* 2021; **5**: 2797–822.

[105] Geng X, Wang F, Wu H *et al.* Data‐driven and artificial intelligence accelerated steel material research and intelligent manufacturing technology. *Materials Genome Engineering Advances* 2023; **1**: e10.

[106] Ball A. Review of Data Management Lifecycle Models. https://purehost.bath.ac.uk/ws/portalfiles/portal/206543/redm1rep120110ab10.pdf (30 April 2025, date last accessed).

[107] Li J, Tu Y, Liu R *et al.* Toward "On‐Demand" materials synthesis and scientific discovery through intelligent robots. *Adv Sci* 2020; **7**: 1901957.

[108] Gongora AE, Snapp KL, Whiting E *et al.* Using simulation to accelerate autonomous experimentation: a case study using mechanics. *iScience* 2021; **24**: 102262.

[109] Kusne AG, Yu H, Wu C *et al.* On-the-fly closed-loop materials discovery via Bayesian active learning. *Nat Commun* 2020; **11**: 5966.

[110] Zhao H, Chen W, Huang H *et al.* A robotic platform for the synthesis of colloidal nanocrystals. *Nat Synth* 2023; **2**: 505–14.

[111] Fakhruldeen H, Pizzuto G, Glowacki J *et al.* ARChemist: autonomous robotic chemistry system architecture. In: *2022 International Conference on Robotics and Automation (ICRA 2022),* Philadelphia, PA, USA. IEEE Computer Society: Los Alamitos, CA, 2022, 6013–9.

[112] Ha T, Lee D, Kwon Y *et al.* AI-driven robotic chemist for autonomous synthesis of organic molecules. *Sci Adv* 2023; **9**: eadj0461.

[113] Slattery A, Wen Z, Tenblad P *et al.* Automated self-optimization, intensification, and scale-up of photocatalysis in flow. *Science* 2024; **383**: eadj1817.

[114] Bateni F, Sadeghi S, Orouji N *et al.* Smart dope: a self‐driving fluidic lab for accelerated development of doped perovskite quantum dots. *Adv Energy Mater* 2024; **14**: 2302303.

[115] Volk AA, Epps RW, Yonemoto DT *et al.* AlphaFlow: autonomous discovery and optimization of multi-step chemistry using a self-driven fluidic lab guided by reinforcement learning. *Nat Commun* 2023; **14**: 1403.

[116] Epps RW, Bowen MS, Volk AA *et al.* Artificial Chemist: An Autonomous Quantum Dot Synthesis Bot. *Adv Mat* 2020; **32**: 2001626.

[117] Boiko DA, MacKnight R, Kline B *et al.* Autonomous chemical research with large language models. *Nature* 2023; **624**: 570–8.

[118] Liang H, Wang C, Yu H *et al.* Real-time experiment-theory closed-loop interaction for autonomous materials science. arXiv: 2410.17430.

[119] Maffettone PM, Friederich P, Baird SG *et al.* What is missing in autonomous discovery: open challenges for the community. *Digit Discov* 2023; **2**: 1644–59.

[120] Lo S, Baird SG, Schrier J *et al.* Review of low-cost self-driving laboratories in chemistry and materials science: the "frugal twin" concept. *Digit Discov* 2024; **3**, 842–68.

[121] Rossano GF, Martinez C, Hedelind M *et al.* Easy robot programming concepts: An industrial perspective. In: *2013 IEEE International Conference on Automation Science and Engineering (CASE 2013)*, Madrid, Spain. IEEE Computer Society: Los Alamitos, CA, 2013, 1119–26.

[122] Bloss R. Collaborative robots are rapidly providing major improvements in productivity, safety, programing ease, portability and cost while addressing many new applications. *IR* 2016;**4 3**: 463–8.

[123] Nikolaev P, Hooper D, Webber F *et al.* Autonomy in materials research: a case study in carbon nanotube growth. *npj Comput Mater* 2016; **2**: 16031.

[124] Roch LM, Häse F, Kreisbeck C *et al.* ChemOS: an orchestration software to democratize autonomous discovery. *PLOS ONE* 2020; **15**: e0229862.

[125] Sim M, Vakili MG, Strieth-Kalthoff F et al. ChemOS 2.0: an orchestration architecture for chemical self-driving laboratories. *Matter* 2024; **7**: 2959–77.

[126] Tamura R, Tsuda K, Matsuda S. NIMS-OS: an automation software to implement a closed loop between artificial intelligence and robotic experiments in materials science. *Sci Technol Adv Material* 2023; **3**: 2232297.

[127] Rahmanian F, Flowers J, Guevarra D *et al.* Enabling modular autonomous feedback‐loops in materials science through hierarchical experimental laboratory automation and orchestration. *Adv Mater Inter* 2022; **9**: 2101987.

[128] Deneault JR, Chang J, Myung J *et al.* Toward autonomous additive manufacturing: Bayesian optimization on a 3D printer. *MRS Bull* 2021; **46**: 566–75.

[129] Bromig L, Leiter D, Mardale A-V *et al.* The SiLA 2 manager for rapid device integration and workflow automation. *SoftwareX* 2022; **17**: 100991.

[130] Pendleton IM, Cattabriga G, Li Z *et al.* Experiment specification, capture and laboratory automation technology (ESCALATE): a software pipeline for automated chemical experimentation and data management. *MRS Commun* 2019; **9**: 846–59.

[131] Steiner S, Wolf J, Glatzel S *et al.* Organic synthesis in a modular robotic system driven by a chemical programming language. *Science* 2019; **363**: eaav2211.

[132] Rauschen R, Guy M, Hein JE *et al.* Universal chemical programming language for robotic synthesis repeatability. *Nat Synth* 2024; **3**: 488–96.

[133] Gromski PS, Granda JM, Cronin L. Universal chemical synthesis and discovery with "The Chemputer. " *Trends Chem* 2020; **2**: 4–12.

[134] Leong CJ, Low KYA, Recatala-Gomez J *et al.* An object-oriented framework to enable workflow evolution across materials acceleration platforms. *Matter* 2022; **5**: 3124–34.

[135] Zhou J, Luo M, Chen L *et al.* A multi-robot–multi-task scheduling system for autonomous chemistry laboratories. *Digit Discov* 2025, **4**: 636–52.

[136] Song T, Luo M, Zhang X *et al.* A multiagent-driven robotic ai chemist enabling autonomous chemical research on demand. *J Am Chem Soc jacs* 2025; **147**: 12534–45.

[137] Google. Agent2Agent (A2A) protocol. https://github.com/a2aproject/A2A (30 April 2025, date last accessed).

[138] Gustafsson OJR, Wilkinson SR, Bacall F *et al.* WorkflowHub: a registry for computational workflows. arXiv: 2410.06941.

[139] KNIME Community Hub. *KNIME Community Hub*. https://hub.knime.com/ (27 May 2025, date last accessed).

[140] Tremouilhac P, Nguyen A, Huang Y-C *et al.* Chemotion ELN: an open source electronic lab notebook for chemists in academia. *J Cheminf* 2017; **9**: 54.

[141] CARPi N, Minges A, Piel M. eLabFTW: an open source laboratory notebook for research labs. *J Open Source Softw* 2017; **2**: 146.

[142] Brandt N, Griem L, Herrmann C *et al.* Kadi4Mat: A Research Data Infrastructure for Materials Science. *Data Science Journal* 2021;**20**, 8-8.

[143] Müller S, Sparka JA, Kuban M *et al.* Grammar-based fuzzing of data integration parsers in computational materials science. *Softw Pract Exper* 2023; **54**: 208–24.

[144] Kononova O, Huo H, He T *et al.* Text-mined dataset of inorganic materials synthesis recipes. *Sci Data* 2019; **6**: 203.

[145] Wang Z, Kononova O, Cruse K *et al.* Dataset of solution-based inorganic materials synthesis procedures extracted from the scientific literature. *Sci Data* 2022; **9**: 231.

[146] Wang W, Jiang X, Tian S *et al.* Automated pipeline for superalloy data by text mining. *npj Comput Mater* 2022; **8**: 9.

[147] Swain MC, Cole JM. ChemDataExtractor: A toolkit for automated extraction of chemical information from the scientific literature. *J Chem Inf Model* 2016; **56**: 1894–904.

[148] Court CJ, Cole JM. Auto-generated materials database of Curie and Néel temperatures via semi-supervised relationship extraction. *Sci Data* 2018; **5**:1 80111.

[149] Huang S, Cole JM. A database of battery materials auto-generated using ChemDataExtractor. *Sci Data* 2020; **7**: 260.

[150] Zhao J, Cole JM. A database of refractive indices and dielectric constants auto-generated using ChemDataExtractor. *Sci Data* 2022; **9**: 192.

[151] Sierepeklis O, Cole JM. A thermoelectric materials database auto-generated from the scientific literature using ChemDataExtractor. *Sci Data* 2022; **9**: 648.

[152] Hettne KM, Stierum RH, Schuemie MJ *et al.* A dictionary to identify small molecules and drugs in free text. *Bioinformatics* 2009; **25**:2 983–91.

[153] Jehangir B, Radhakrishnan S, Agarwal R. A survey on named entity recognition — datasets, tools, and methodologies. *Natural Language Processing Journal* 2023; **3**: 100017.

[154] Keraghel I, Morbieu S, Nadif M. Recent advances in named entity recognition: a comprehensive survey and comparative study. arXiv: 2401.10825.

[155] Pawar S, Palshikar GK, Bhattacharyya P. Relation extraction : a survey. 2017, arXiv: 1712.05191.

[156] Detroja K, Bhensdadia CK, Bhatt BS. A survey on Relation Extraction. *Intelligent Systems with Applications* 2023; **19**: 200244.

[157] Mavračić J, Court CJ, Isazawa T *et al.* ChemDataExtractor 2.0: autopopulated ontologies for materials science. *J Chem Inf Model* 2021; **61**: 4280–9.

[158] Giorgi J, Bader G, Wang B. A sequence-to-sequence approach for document-level relation extraction. In: *21st Workshop on Biomedical Language Processing (BioNLP 2022)*, Dublin, Ireland. Association for Computational Linguistics: Stroudsburg, PA, 2022, 10–25.

[159] Huguet Cabot P-L, Navigli R. REBEL: Relation Extraction By End-to-end Language generation. In: *Findings of the Association for Computational Linguistics: EMNLP 2021*, Punta Cana, Dominican Republic. Association for Computational Linguistics: Stroudsburg, PA, 2021, 2370–81.

[160] Gururangan S, Marasović A, Swayamdipta S *et al.* Don't Stop Pretraining: Adapt Language Models to Domains and Tasks. *Proceedings of the 58th Annual Meeting of the Association for Computational Linguistics*. Online: Association for Computational Linguistics, 2020, 8342–60.

[161] Trewartha A, Walker N, Huo H *et al.* Quantifying the advantage of domain-specific pre-training on named entity recognition tasks in materials science. *Patterns* 2022; **3**: 100488.

[162] Joshi M, Chen D, Liu Y *et al.* SpanBERT: Improving Pre-training by representing and predicting spans. *TACL* 2020; **8**64–77.

[163] Gupta T, Zaki M, Krishnan NMA *et al.* MatSciBERT: a materials domain language model for text mining and information extraction. *Npj Comput Mater* 2022; **8**: 102.

[164] Shetty P, Rajan AC, Kuenneth C *et al.* A general-purpose material property data extraction pipeline from large polymer corpora using natural language processing. *npj Comput Mater* 2023; **9**: 52.

[165] Gilligan LPJ, Cobelli M, Taufour V *et al.* A rule-free workflow for the automated generation of databases from scientific literature. *npj Comput Mater* 2023; **9**: 1–14.

[166] Polak MP, Morgan D. Extracting accurate materials data from research papers with conversational language models and prompt engineering. *Nat Commun* 2024; **15**: 1569.

[167] Qai222. *Extracting Structured Data from Organic Synthesis Procedures Using a Fine-Tuned Large Language Model*. https://github.com/qai222/LLM_organic_synthesis (12 April 2025, last date accessed)

[168] Kojima T, Gu SS, Reid M *et al.* Large language models are zero-shot reasoners. arXiv: 2205.11916.

[169] Dagdelen J, Dunn A, Lee S *et al.* Structured information extraction from scientific text with large language models. *Nat Commun* 2024; **15**: 1418.

[170] Choi J, Lee B. Accelerating materials language processing with large language models. *Commun Mater* 2024; **5**: 13.

[171] Gupta S, Mahmood A, Shetty P *et al.* Data extraction from polymer literature using large language models. *Commun Mater* 2024; **5**: 269.

[172] Crewe AV. Scanning electron microscopes: is high resolution possible?: use of a field-emission electron source may make it possible to overcome existing limitations on resolution. *Science* 1966; **154**: 729–38.

[173] Pennycook SJ, Nellist PD eds. *Scanning Transmission Electron Microscopy*. In: Hawkes PW (ed.). *Science of microscopy*. New York: Springer New York, 2007, 65–132.

[174] Jenkins R, Snyder RL, others. *Introduction to X-Ray Powder Diffractometry*. Hoboken, NJ: John Wiley & Sons, 1996, 121-50.

[175] Bunaciu AA, UdriŞTioiu EG, Aboul-Enein HY. X-ray diffraction: instrumentation and applications. *Crit Rev Ana Chem* 2015; **45**: 289–99.

[176] Gerber C, Lang HP. How the doors to the nanoworld were opened. *Nat Nanotech* 2006; **1**: 3–5.

[177] Kalinin SV, Sumpter BG, Archibald RK. Big–deep–smart data in imaging for guiding materials design. *Nat Mater* 2015; **14**: 973–80.

[178] Mobarak MH, Mimona MA, Islam MdA *et al.* Scope of machine learning in materials research—a review. *Appl Surf Sci Adv* 2023; **18**: 100523.

[179] Stuckner J, Harder B, Smith TM. Microstructure segmentation with deep learning encoders pre-trained on a large microscopy dataset. *npj Comput Mater* 2022; **8**: 200.

[180] Mukaddem KT, Beard EJ, Yildirim B *et al.* ImageDataExtractor: a tool to extract and quantify data from microscopy images. *J Chem Inf Model* 2020; **60**: 2492–509.

[181] Beard EJ, Cole JM. ChemSchematicResolver: A toolkit to decode 2d chemical diagrams with labels and r-groups into annotated chemical named entities. *J Chem Inf Model* 2020; **60**: 2059–72.

[182] Li Y, Liu Y, Luo S *et al.* Neural network model for correlating microstructural features and hardness properties of nickel-based superalloys. *J Mater Res Technol* 2020; **9**:1 4467–77.

[183] Furat O, Finegan DP, Diercks D *et al.* Mapping the architecture of single lithium ion electrode particles in 3D, using electron backscatter diffraction and machine learning segmentation. *J Power Sources* 2021; **483**: 229148.

[184] Stein HS, Guevarra D, Newhouse PF *et al.* Machine learning of optical properties of materials – predicting spectra from images and images from spectra. *Chem Sci* 2019; **10**: 47–55.

[185] Li C, Han X, Yao C *et al.* A novel training-free approach to efficiently extracting material microstructures via visual large model. *Acta Mater* 2025; **290**: 120962.

[186] Balestriero R, Ibrahim M, Sobal V *et al.* A Cookbook of Self-Supervised Learning. arXiv: 2304.12210.

[187] Rani V, Nabi ST, Kumar M *et al.* Self-supervised learning: a succinct review. *Arch Computat Methods Eng* 2023; **30**: 2761–75.

[188] Gupta T, Zaki M, Khatsuriya D *et al.* DiSCoMaT: Distantly Supervised Composition Extraction from Tables in Materials Science Articles. In: *61st Annual Meeting of the Association for Computational Linguistics (ACL 2023),* Toronto, Canada. Association for Computational Linguistics: Stroudsburg, PA, 2023, 13465–83.

[189] Hira K, Zaki M, Sheth D *et al.* Reconstructing the materials tetrahedron: challenges in materials information extraction. *Digit Discov* 2024; **3**: 1021–37.

[190] Limaye G, Sarawagi S, Chakrabarti S. Annotating and searching web tables using entities, types and relationships. *Proc VLDB Endow* 2010; **3**: 1338–47.

[191] Deng J, Liu G, Wang L *et al.* An efficient extraction method of journal-article table data for data-driven applications. *Inform Process Manag* 2025; **62**: 104006.

[192] Nishida K, Sadamitsu K, Higashinaka R *et al.* Understanding the Semantic Structures of Tables with a Hybrid Deep Neural Network Architecture. In: *AAAI Conference on Artificial Intelligence (AAAI 2017),* San Francisco, CA, USA. AAAI Press: Menlo Park, CA, 2017, 31(1).

[193] Microsoft. *Table-transformer (TATR).* https://github.com/microsoft/table-transformer (11 April 2025, date last accessed).

[194] Kawakatsu T. Multi-cell Decoder and Mutual Learning for Table Structure and Character Recognition. In: Barney Smith EH, Liwicki M, Peng L (eds.). *Document Analysis and Recognition - ICDAR 2024*. Vol 14804. Cham: Springer Nature Switzerland, 2024, 389–405.

[195] Ye J, Qi X, He Y *et al.* PingAn-VCGroup's solution for ICDAR 2021 competition on scientific literature parsing task b: table recognition to html. arXiv: 2105.01848.

[196] Cao L. TableMaster: A recipe to advance table understanding with language models. arXiv: 2501.19378.

[197] Zha L, Zhou J, Li L *et al.* TableGPT: Towards Unifying Tables, Nature Language and Commands into One GPT. arXiv: 2307.08674.

[198] Zhang T, Yue X, Li Y *et al.* TableLlama: Towards Open Large Generalist Models for Tables. In: *2024 Conference of the North American Chapter of the Association for Computational Linguistics (NAACL 2024),* Mexico City, Mexico. Association for Computational Linguistics: Stroudsburg, PA, 2024, 6024–44.

[199] Herzig J, Nowak PK, Müller T *et al.* TaPas: Weakly Supervised Table Parsing via Pre-training. In: *58th Annual Meeting of the Association for Computational Linguistics (ACL 2020),* Online. Association for Computational Linguistics: Stroudsburg, PA, 2020, 4320–33.

[200] Gu Z, Fan J, Tang N *et al.* PASTA: Table-Operations Aware Fact Verification via Sentence-Table Cloze Pre-training. *Proceedings of the 2022 Conference on Empirical Methods in Natural Language Processing*. Abu Dhabi, United Arab Emirates: Association for Computational Linguistics, 2022, 4971–83.

[201] Lewis P, Perez E, Piktus A *et al.* Retrieval-augmented generation for knowledge-intensive NLP tasks. In: *34th International Conference on Neural Information Processing Systems (NIPS '20),* Vancouver, BC, Canada. Curran Associates Inc.: Red Hook, NY, 2020, 2020, 9459–74.

[202] Luo J, Li Z, Wang J *et al.* ChartOCR: Data Extraction from Charts Images via a Deep Hybrid Framework. *2021 IEEE Winter Conference on Applications of Computer Vision (WACV)*. Waikoloa, HI, USA: IEEE, 2021, 1916–24.

[203] Kato H, Nakazawa M, Yang H-K *et al.* Parsing line chart images using linear programming. In: *IEEE/CVF Winter Conference on Applications of Computer Vision (WACV)*, Waikoloa, HI, USA. IEEE Computer Society: Los Alamitos, CA, 2022, 2553–62.

[204] Liu Z, Lin Y, Cao Y *et al.* Swin Transformer: hierarchical vision transformer using shifted windows. arXiv: 2103.14030.

[205] P SV, Yusuf Hassan M, Singh M. LineEX: data extraction from scientific line charts. In: *IEEE/CVF Winter Conference on Applications of Computer Vision (WACV)*, Waikoloa, HI, USA. IEEE Computer Society: Los Alamitos, CA, 2023, 6213–6221.

[206] Davila K, Setlur S, Doermann D *et al.* Chart Mining: A Survey of Methods for Automated Chart Analysis. *IEEE Trans Pattern Anal Mach Intell* 2021; **43**: 3799–819.

[207] Lal J, Mitkari A, Bhosale M *et al.* LineFormer: Line Chart Data Extraction Using Instance Segmentation. In: Fink GA, Jain R, Kise K, et al. (eds.). *Document Analysis and Recognition - ICDAR 2023*. Vol 14191. Cham: Springer Nature Switzerland, 2023, 387–400.

[208] Liu F, Eisenschlos JM, Piccinno F *et al.* DePlot: one-shot visual language reasoning by plot-to-table translation. 2022, arXiv: 2212.10505.

[209] Rane C, Subramanya SM, Endluri DS *et al.* ChartReader: Automatic Parsing of Bar-Plots. In: *22nd IEEE International Conference on Information Reuse and Integration for Data Science (IRI)*, Las Vegas, NV, USA. IEEE Computer Society: Los Alamitos, CA, 2021, 318–25.

[210] Katsura Y, Kumagai M, Kodani T *et al.* Data-driven analysis of electron relaxation times in PbTe-type thermoelectric materials. *Sci Technol Adv Mater* 2019; **20**: 511–20.

[211] Chard K, D'Arcy M, Heavner B *et al.* I'll take that to go: Big data bags and minimal identifiers for exchange of large, complex datasets. In: *2016 IEEE International Conference on Big Data (Big Data)*, Washington, DC, USA. IEEE Computer Society: Los Alamitos, CA, 2016, 319–28.

[212] ORCID Inc. Open Researcher and Contributor ID (ORCID). https://orcid.org/ (21 May 2025, date last accessed).

[213] Rivest R. A review paper of message digest 5 (MD5). *International Journal of Modern Engineering & Management Research* 2013, 1.4: 29-35.

[214] Foster I. Globus Online: Accelerating and democratizing science through cloud-based services. *IEEE Internet Comput* 2011; **15**: 70–3.

[215] Allen B, Bresnahan J, Childers L *et al.* Software as a service for data scientists. *Commun ACM* 2012; **55**: 81–8.

[216] Jain A, Ong SP, Hautier G *et al.* Commentary: The materials project: a materials genome approach to accelerating materials innovation. *APL Materials* 2013; **1**: 011002.

[217] Curtarolo S, Setyawan W, Wang S *et al.* AFLOWLIB.ORG: a distributed materials properties repository from high-throughput ab initio calculations. *Comput Mater Sci* 2012; **58**: 227–35.

[218] Esters M, Oses C, Divilov S *et al.* Aflow.org: a web ecosystem of databases, software and tools. *Comput Mater Sci* 2023; **216**: 111808.

[219] Kirklin S, Saal JE, Meredig B *et al.* The open quantum materials database (OQMD): assessing the accuracy of DFT formation energies. *npj Comput Mater* 2015; **1**: 1–15.

[220] Shen J, Griesemer SD, Gopakumar A *et al.* Reflections on one million compounds in the open quantum materials database (OQMD). *J Phys Mater* 2022; **5**: 031001.

[221] Scheidgen M, Himanen L, Ladines AN *et al.* NOMAD: a distributed web-based platform for managingmaterials science research data. *JOSS* 2023; **8**: 5388.

[222] Talirz L, Kumbhar S, Passaro E *et al.* Materials Cloud, a platform for open computational science. *Sci Data* 2020; **7**: 299.

[223] Choudhary K, Garrity KF, Reid ACE *et al.* JARVIS: an integrated infrastructure for data-driven materials design. arXiv: 2007.01831.

[224] Choudhary K, Garrity KF, Reid ACE *et al.* The joint automated repository for various integrated simulations (JARVIS) for data-driven materials design. *npj Comput Mater* 2020; **6**: 173.

[225] Puchala B, Tarcea G, Marquis EmmanuelleA *et al.* The materials commons: a collaboration platform and information repository for the global materials community. *JOM* 2016; **68**: 2035–44.

[226] Tarcea G, Puchala B, Berman T *et al.* The materials commons data repository. In: IE*EE 18th International Conference on E-Scienc*e (e-Science), Salt Lake City, UT, USA. New York: IEEE Press, 2022, 405–6.

[227] Blaiszik B, Chard K, Pruyne J *et al.* The materials data facility: data services to advance materials science research. *JOM* 2016; **68**: 2045–52.

[228] Blaiszik B, Ward L, Schwarting M *et al.* A data ecosystem to support machine learning in materials science. *MRS Commun* 2019; **9**: 1125–33.

[229] Liu S, Su Y, Yin H *et al.* An infrastructure with user-centered presentation data model for integrated management of materials data and services. *npj Comput Mater* 2021;**7**:88.

[230] Zakutayev A, Wunder N, Schwarting M *et al.* An open experimental database for exploring inorganic materials. *Sci Data* 2018; **5**: 180053.

[231] Wang Y, Xiao J, Suzek TO *et al.* PubChem: a public information system for analyzing bioactivities of small molecules. *Nucleic acids res* 2009; **37**: W623–33.

[232] Kim S, Thiessen PA, Bolton EE *et al.* PubChem substance and compound databases. *Nucleic acids res* 2016; **44**: D1202–13.

[233] Kim S, Chen J, Cheng T *et al.* PubChem 2023 update. *Nucleic acids res* 2023; **51**: D1373–80.

[234] Pence HE, Williams A. ChemSpider: an online chemical information resource. *J Chem Educ* 2010; **87**: 1123–4.

[235] Otsuka S, Kuwajima I, Hosoya J *et al.* PoLyInfo: polymer database for polymeric materials design. In: *2011 International Conference on Emerging Intelligent Data and Web Technologies (EIDWT)*, Denpasar, Bali, Indonesia. IEEE Computer Society: Los Alamitos, CA, 2011, 22–29.

[236] Zhao H, Li X, Zhang Y *et al.* Perspective: nanomine: a material genome approach for polymer nanocomposites analysis and design. *APL Mater*2016; **4**: 053204.

[237] Zhao H, Wang Y, Lin A *et al.* NanoMine schema: an extensible data representation for polymer nanocomposites. *APL Mater* 2018; **6**: 111108.

[238] Brinson LC, Deagen M, Chen W *et al.* Polymer nanocomposite data: curation, frameworks, access, and potential for discovery and design. *ACS Macro Lett* 2020; **9**:1 086–94.

[239] Hummelshøj JS, Abild‐Pedersen F, Studt F *et al.* CatApp: a web application for surface chemistry and heterogeneous catalysis. *Angew Chem Int Edit* 2012; **124**: 278–80.

[240] Pepponi G, Gražulis S, Chateigner D. MPOD: a material property open database linked to structural information. *Nucl Instrum Meth B* 2012; **284**: 10–4.

[241] Fuentes-Cobas LE, Chateigner D, Fuentes-Montero ME *et al.* The representation of coupling interactions in the Material Properties Open Database (MPOD). *Adv Appl Ceram* 2017; **116**: 428–33.

[242] Statt MJ, Rohr BA, Guevarra D *et al.* The materials provenance store. *Sci Data* 2023; **10**: 184.

[243] Soedarmadji E, Stein HS, Suram SK *et al.* Tracking materials science data lineage to manage millions of materials experiments and analyses. n*pj Comput Mater* 2019; **5**: 1–9.
[244] Moreira J, Bonino da Silva Santos LO, Ferreira Pires L *et al.* Towards findable, accessible, interoperable and reusable (FAIR) data repositories: improving a data repository to behave as a FAIR data point. *Liinc em Revista* 2019;**15**.
[245] Da Silva Santos LOB, Burger K, Kaliyaperumal R *et al.* FAIR data point: a FAIR-oriented approach for metadata publication. *DI* 2023; **5**: 163–83.
[246] L'Hours H, Stein IV, Huigen F *et al.* CoreTrustSeal plus FAIR overview. Tech Serv Q 2019; **36**: 61–72.
[247] RLG-OCLC. Trusted Digital Repositories: Attributes and Responsibilities. https://www.oclc.org/content/dam/research/activities/trustedrep/repositories.pdf (9 April 2025, date last accessed).
[248] Lin D, Crabtree J, Dillo I *et al.* The TRUST Principles for digital repositories. *Sci Data* 2020; **7**: 144.
[249] Consultative Committee for Space Data Systems (CCSDS). Audit and certification of trustworthy digital repositories: recommended practice. https://ccsds.org/Pubs/652x0m2.pdf (9 April 2025, date last accessed).
[250] Pournaghi SM, Bayat M, Farjami Y. MedSBA: a novel and secure scheme to share medical data based on blockchain technology and attribute-based encryption. *J Ambient Intell Human Comput* 2020; **11**: 4613–41.
[251] Lu X, Fu S, Jiang C *et al.* A fine-grained IoT data access control scheme combining attribute-based encryption and blockchain. *Secur Commun Netw* 2021; **2021**: 1–13.
[252] Liu A, Wang N, Du X *et al.* Big data access control mechanism based on two-layer permission decision structure. *Comput Mater Continua* 2024; **79**: 1705–26.
[253] Yue L, Junqin H, Shengzhi Q *et al.* Big Data Model of Security Sharing Based on Blockchain. *2017 3rd International Conference on Big Data Computing and Communications (BIGCOM)*. Chengdu: IEEE, 2017, 117–21.
[254] Liang X, Shetty S, Tosh D *et al.* ProvChain: A Blockchain-Based Data Provenance Architecture in Cloud Environment with Enhanced Privacy and Availability. In: *17th IEEE/ACM International Symposium on Cluster, Cloud and Grid Computing (CCGRID),* Madrid, Spain. IEEE Computer Society: Los Alamitos, CA, 2017, 468–77.
[255] Ramachandran A, Kantarcioglu M. SmartProvenance: a distributed, blockchain based dataprovenance system. In: *Eighth ACM Conference on Data and Application Security and Privacy (CODASPY '18),* Tempe, AZ, USA. Association for Computing Machinery: New York, NY, 2018, 35–42.
[256] Yang J, Wen J, Jiang B *et al.* Blockchain-based sharing and tamper-proof framework of big data networking. *IEEE Net* 2020; **34**: 62–7.
[257] Lyu Q, Zhou Y, Ren Y *et al.* Toward personal data sharing autonomy: a task-driven data capsule sharing system. *IEEE Trans Inf Forensic Secur* 2024; **19**: 9760–74.
[258] Castro M, Liskov B, others. Practical byzantine fault tolerance and proactive recovery. ACM Trans Comput Syst, 2002; **20**: 398–461.
[259] Wang Y, Zhong M, Cheng T. Research on PBFT consensus algorithm for grouping based on feature trust. *Sci Rep* 2022; **12**: 12515.
[260] Raghav, Andola N, Venkatesan S *et al.* PoEWAL: a lightweight consensus mechanism for blockchain in IoT. *Pervasive Mob Comput* 2020; **69**: 101291.
[261] Fu W, Wei X, Tong S. An Improved blockchain consensus algorithm based on raft. *Arab J Sci Eng* 2021; **46**: 8137–49.

[262] Wei Y, Xu Q, Peng H. An enhanced consensus algorithm for blockchain. *Sci Rep* 2024; **14**: 17701.
[263] Picklum M, Beetz M. MatCALO: knowledge-enabled machine learning in materials science. *Comput Mater Sci* 2019; **163**: 50–62.
[264] Aggour KS, Detor A, Gabaldon A *et al.* Compound knowledge graph-enabled ai assistant for accelerated materials discovery. *Integr Mater Manuf Innov* 2022; **11**: 467–78.
[265] Hogan A, Blomqvist E, Cochez M *et al.* Knowledge graphs. *ACM Comput Surv* 2022; **54**: 1–37.
[266] Venugopal V, Olivetti E. MatKG: an autonomously generated knowledge graph in material science. *Sci Data* 2024; **11**: 217.
[267] Zhang X, Liu X, Li X *et al.* MMKG: An approach to generate metallic materials knowledge graph based on DBpedia and Wikipedia. *Comput Phys Commun* 2017; **211**: 98–112.
[268] Statt MJ, Rohr BA, Guevarra D *et al.* The materials experiment knowledge graph. *Digit Discov* 2023; **2**: 909–14.
[269] Mrdjenovich D, Horton MK, Montoya JH *et al.* Propnet: a knowledge graph for materials science. *Matter* 2020; **2**: 464–80.
[270] McCusker JP, Keshan N, Rashid S *et al.* NanoMine: a knowledge graph for nanocomposite materials science. In: Pan JZ, Tamma V, d'Amato C, et al. (eds.). *The Semantic Web – ISWC 2020*. Vol 12507. Cham: Springer International Publishing, 2020, 144–59.
[271] MatPortal.org. Materials Open Laboratory MatPortal. https://matportal.org/ (1 July 2024, date last accessed).
[272] De Baas A, Nostro PD, Friis J *et al.* Review and alignment of domain-level ontologies for materials science. *IEEE Access* 2023; **11**: 120372–401.
[273] Ghedini E, Hashibon A, Friis J *et al.* EMMO the european materials modelling ontology. https://emmo-repo.github.io/versions/0.9.9/emmo.pdf (20 May 2025,date last accessed).
[274] Arp R, Smith B. Function, role, and disposition in basic formal ontology. *Nat Prec* 2008; 1:1.
[275] Niles I, View Profile, Pease A *et al.* Towards a standard upper ontology. In: *International Conference on Formal Ontology in Information Systems (FOIS '01)*, Ogunquit, Maine, USA. Association for Computing Machinery: New York, NY, 2001, 2–9.
[276] Herre H. General Formal Ontology (GFO): A foundational ontology for conceptual modelling. In: Poli R, Healy M, Kameas A (eds.). *Theory and Applications of Ontology: Computer Applications*. Dordrecht: Springer Netherlands, 2010, 297–345.
[277] Gangemi A, Guarino N, Masolo C *et al.* Sweetening ontologies with DOLCE. In: Gómez-Pérez A, Benjamins VR (eds.). *Knowledge Engineering and Knowledge Management: Ontologies and the Semantic Web*. Berlin, Heidelberg: Springer, 2002, 166–81.
[278] Voigt SP, Kalidindi SR. Materials graph ontology. *Mater Lett* 2021; **295**: 129836.
[279] Bayerlein B, Schilling M, Birkholz H *et al.* PMD core ontology: achieving semantic interoperability in materials science. *Mat Des* 2024; **237**: 112603.
[280] Zhang X, Zhao C, Wang X. A survey on knowledge representation in materials science and engineering: an ontological perspective. *Comput Ind* 2015; **73**: 8–22.
[281] Vardeman CF, Krisnadhi AA, Cheatham M *et al.* An ontology design pattern and its use case for modeling material transformation. De Boer V, Ławrynowicz A (eds.). *Semant Web* 2017; **8**: 719–31.
[282] Fernández-López M, Gómez-Pérez A, Juristo Juzgado N. Methontology: from ontological art towards ontological engineering. In: *AAAI-97 Spring Symposium Series, Stanford University*, USA. AAAI Press: Menlo Park, CA, 1997.

[283] Sure Y, Staab S, Studer R. On-To-Knowledge Methodology (OTKM). In: Staab S, Studer R (eds.). *Handbook on Ontologies*. Berlin, Heidelberg: Springer Berlin Heidelberg, 2004, 117–32.
[284] Noy NF, McGuinness DL. Ontology development 101: A guide to creating your first ontology. https://course.ccs.neu.edu/cs5100f11/resources/noy01.pdf/ (20 May 2025,date last accessed).
[285] Jarrar M, Meersman R. Ontology engineering – the dogma approach. In: Dillon TS, Chang E, Meersman R, et al. (eds.). *Advances in Web Semantics I*. Vol 4891. Berlin, Heidelberg: Springer Berlin Heidelberg, 2008, 7–34.
[286] De Nicola A, Missikoff M, Navigli R. A software engineering approach to ontology building. *Inform Syst* 2009; **34**: 258–75.
[287] FAIRsharing Team. FAIRsharing record for: quantities, units, dimensions and types. https://fairsharing.org/10.25504/FAIRsharing.d3pqw7 (20 May 2025,date last accessed).
[288] W3C groups. PROV-O: The PROV Ontology. https://www.w3.org/TR/prov-o/ (20 May 2025, date last accessed).
[289] CSVW.org. CSVW - CSV on the Web. https://csvw.org/ (20 May 2025,date last accessed).
[290] Stuckenschmidt H, Parent C, Spaccapietra S eds. *Modular Ontologies*. Berlin, Heidelberg: Springer Berlin Heidelberg, 2009, 293–6.
[291] Astrova I. Reverse Engineering of Relational Databases to Ontologies. In: Bussler CJ, Davies J, Fensel D, et al. (eds.). *The Semantic Web: Research and Applications*. Vol 3053. Berlin, Heidelberg: Springer Berlin Heidelberg, 2004, 327–41.
[292] Man Li, Xiao-Yong Du, Shan Wang. Learning ontology from relational database. In: *2005 International Conference on Machine Learning and Cybernetics*, Guangzhou, China. IEEE: New York, 2005, 3410-3415 Vol. 6.
[293] Zhou S, Meng G, Ling H *et al*. Tool for translating relational databases schema into ontology for semantic web. In: *2010 Second International Workshop on Education Technology and Computer Science*, Wuhan, China. IEEE: New York, 2010, 198–201.
[294] Asfand-E-Yar M, Ali R. Semantic Integration of Heterogeneous Databases of Same Domain Using Ontology. *IEEE Access* 2020; **8**: 77903–19.
[295] Suárez-Figueroa MC, Gómez-Pérez A, Fernández-López M. The NeOn methodology for ontology engineering. In: Suárez-Figueroa MC, Gómez-Pérez A, Motta E, et al. (eds.). *Ontology Engineering in a Networked World*. Berlin, Heidelberg: Springer Berlin Heidelberg, 2012, 9–34.
[296] Li H, Armiento R, Lambrix P. A method for extending ontologies with application to the materials science domain. *Data Sci J* 2019; **18**: 50.
[297] Stanford Center for Biomedical Informatics Research. Protégé, v 5.5.0. MA: Stanford Center for Biomedical Informatics Research; 2019
[298] Dudáš M, Hanzal T, Svátek V *et al*. OBOWLMorph: Starting Ontology Development from PURO Background Models. In: I*nternational Experiences and Directions Workshop on OWL (OWLED 2015),* Amsterdam, Netherlands. Springer International Publishing: Cham, 2015, 14–20.
[299] Chen Y, Schilling M, von Hartrott P *et al*. Ontopanel: a tool for domain experts facilitating visual ontology development and mapping for FAIR data sharing in materials testing. *Integr Mater Manuf Innov* 2022; **11**: 545–56.
[300] Ashino T. Materials ontology: an infrastructure for exchanging materials information and knowledge. *Data Sci J* 2010; **9**: 54–61.

[301] Li H, Armiento R, Lambrix P. An ontology for the materials design domain. In: *International Semantic Web Conference (ISWC 2020)*, Athens, Greece. Springer International Publishing: Cham, 2020, 212–227.
[302] Lambrix P, Armiento R, Li H *et al.* The materials design ontology. *Semant Web* 2024; 15: 481–515.
[303] Piane FL, Baldoni M, Gaspari M *et al.* Introducing MAMBO: Materials and molecules basic ontology. arXiv: 2111.02482
[304] Medina-Smith A, Becker CA, Plante RL *et al.* A controlled vocabulary and metadata schema for materials science data discovery. *Data Sci J* 2021; **20**: 18.
[305] Horsch MT, Chiacchiera S, Seaton MA *et al.* Ontologies for the Virtual Materials Marketplace. 2020.
[306] Dutta N, DeBellis M. Dental restorative material ontology (DrMO). In: Aussenac-Gilles N, Hahmann T, Galton A, *et al*. (eds.). *Frontiers in Artificial Intelligence and Applications*. Amsterdam: IOS Press, 2023, 288–301.
[307] Hakimi O, Gelpi JL, Krallinger M *et al.* The devices, experimental scaffolds, and biomaterials ontology (deb): a tool for mapping, annotation, and analysis of biomaterials data. *Adv Funct Mater* 2020; **30**: 1909910.
[308] Cheung K. Towards an ontology for data-driven discovery of new materials. In: *AAAI Spring Symposium: Semantic Scientific Knowledge Integration*, Stanford, California, USA. AAAI Press: Menlo Park, CA, 2008, 9–14.
[309] Thomas DG, Pappu RV, Baker NA. NanoParticle Ontology for cancer nanotechnology research. *J Biomed Inf* 2011; **44**: 59–74.
[310] Hastings J, Jeliazkova N, Owen G *et al.* eNanoMapper: harnessing ontologies to enable data integration for nanomaterial risk assessment. *J Biomed Semant* 2015; **6**: 10.
[311] van der Vet PE, Speel P-H, Mars N. The Plinius ontology of ceramic materials. In: *the 11th European Conference on Artificial Intelligence (ECAI'94)*, Amsterdam, Netherlands. John Wiley & Sons: Chichester, 1994.
[312] Premkumar V, Krishnamurty S, Wileden JC *et al.* A semantic knowledge management system for laminated composites. *Adv Eng Inf* 2014; **28**: 91–101.
[313] Furini F, Rai R, Smith B *et al.* Development of a Manufacturing Ontology for Functionally Graded Materials. In: *36th Computers and Information in Engineering Conference*, Charlotte, North Carolina, USA. American Society of Mechanical Engineers: New York, 2016, V01BT02A030.
[314] Mohd Ali M, Yang R, Zhang B *et al.* Enriching the functionally graded materials (FGM) ontology for digital manufacturing. *Int J Prod Res* 2021; **59**: 5540–57.
[315] Zhang X, Pan D, Zhao C *et al.* MMOY: Towards deriving a metallic materials ontology from Yago. *Adv Eng Inf* 2016; **30**: 687–702.
[316] Radinger A, Rodriguez-Castro B, Stolz A *et al.* BauDataWeb: the Austrian building and construction materials market as linked data. In: *International Conference on Semantic Systems*, Graz, Austria. Association for Computing Machinery: New York, USA, 2013, 25–32.
[317] Materials Genome Engineering. materials genome engineering - terminology. http://www.cstm.com.cn/article/details/a03df707-fc39-4c9c-a80a-2012b705ffa0 (15 May 2025, date last accessed).
[318] Materials Genome Engineering. materials genome engineering - materials data identifier（MID）. http://www.cstm.com.cn/article/details/517131f5-0ea8-45ad-9595-558fd9f9da7a (15 May 2025, date last accessed).

[319] Materials Genome Engineering. materials calculation data - general requirements. http://www.cstm.com.cn/article/details/1ba5a29f-ec80-4986-93a5-0205e2d0838f (15 May 2025, date last accessed).
[320] Materials Genome Engineering. materials experimental data - general requirements. http://www.cstm.com.cn/article/details/4535f536-5283-4bd4-8b3e-7a7117010b11 (15 May 2025, date last accessed).
[321] Krahl R, Darroch L, Huber R *et al.* Persistent identification of instruments. arXiv:2003.12958.
[322] Schmitz GJ, Böttger B, Apel M *et al.* Towards a metadata scheme for the description of materials – the description of microstructures. *Sci Technol Adv Mater* 2016; **17**: 410–30.
[323] Thomas DG, Gaheen S, Harper SL *et al.* ISA-TAB-Nano: A specification for sharing nanomaterial research data in spreadsheet-based format. *BMC Biotechnol* 2013; **13**: 2.
[324] Rao Y, Lu Y, Zhang L *et al.* A metadata schema for lattice thermal conductivity from first-principles calculations. *J Mater Inf* 2022; **2**: 17.
[325] Ghiringhelli LM, Carbogno C, Levchenko S *et al.* Towards efficient data exchange and sharing for big-data driven materials science: metadata and data formats. *npj Comput Mater* 2017; **3**: 46.
